# The iSelect 9 K SNP analysis revealed polyploidization induced revolutionary changes and intense human selection causing strong haplotype blocks in wheat

**DOI:** 10.1038/srep41247

**Published:** 2017-01-30

**Authors:** Chenyang Hao, Yuquan Wang, Shiaoman Chao, Tian Li, Hongxia Liu, Lanfen Wang, Xueyong Zhang

**Affiliations:** 1 Key Laboratory of Crop Gene Resources and Germplasm Enhancment, Ministry of Agriculture/The National Key Facility for Crop Gene Resources and Genetic Improvement/Institute of Crop Science, Chinese Academy of Agricultural Sciences, Beijing 100081, China; 2 US Department of Agriculture–Agricultural Research Service Biosciences Research Laboratory, Fargo, ND 58102, USA

## Abstract

A Chinese wheat mini core collection was genotyped using the wheat 9 K iSelect SNP array. Total 2420 and 2396 polymorphic SNPs were detected on the A and the B genome chromosomes, which formed 878 haplotype blocks. There were more blocks in the B genome, but the average block size was significantly (*P* < 0.05) smaller than those in the A genome. Intense selection (domestication and breeding) had a stronger effect on the A than on the B genome chromosomes. Based on the genetic pedigrees, many blocks can be traced back to a well-known Strampelli cross, which was made one century ago. Furthermore, polyploidization of wheat (both tetraploidization and hexaploidization) induced revolutionary changes in both the A and the B genomes, with a greater increase of gene diversity compared to their diploid ancestors. Modern breeding has dramatically increased diversity in the gene coding regions, though obvious blocks were formed on most of the chromosomes in both tetraploid and hexaploid wheats. Tag-SNP markers identified in this study can be used for marker assisted selection using haplotype blocks as a wheat breeding strategy. This strategy can also be employed to facilitate genome selection in other self-pollinating crop species.

Common wheat (*Triticum aestivum* L.) is one of the most important global staple food. It was originated in the middle east about 8000 years ago and expanded to most parts of the world^1^. Intense human selection, including both domestication and modern breeding contributed to the development of different ecotypes and cultivars. Bottleneck effect is a common case in species domestication^2^,^3^,^4^. In wheat, domestication was further accompained by two steps of polyploidization, tetraploidization and hexaploidization. Domestication occurred in diploid A genome donors, such as *Triticum monococcum*, and continued in tetraploid and hexaploid wheats. Therefore, besides common evolution effects found in other crops such as maize, rice, soybean, etc., polyploidization played additional important role in shaping the wheat evolution. Tracking the footprints of wheat evolution by molecular markers is an interesting but challenging research, because until now, all of the published results concerning wheat domestication was mainly based on single genes^5^,^6^.

Wheat breeding crosses started at the beginning of last century. An Italian breeder, N. Strampelli, made a very famous cross involving three parents, Rieti/Wilhelmina//Akagomughi, from which several excellent cultivars were derived, such as Ardito, Mentana, Villa Gloria, etc., and these cultivars and their improved lines formed the base of global wheat cultivars^7^,^8^. For example, Mentana was used as a parent to create 110 new cultivars during 1949–1961 in China. Its derived Abbondanza was further used to develop 217 new cultivars during 1961–1979 in China. Aimengniu, an improved new cultivar of Abbondanza, won the best national innovation award in 1997, and was widely used as a founder genotype in wheat breeding programs in China. One of Villa Gloria derivatives, St 2422–464 (St), was also used to create 122 new varieties during 1970–1990 in China. Several derivatives of St, in particular Xiaoyan 6 and Yumai 18, made a large impact on national wheat production and breeding in China. A set of 56 new varieties was established from crossing Xiaoyan 6 with other cultivars. From which, Zhengmai 9023, Xinong 979, and Zhengmai 366 are widely grown in China currently. The cultivars 9023 and 366 won the best and the second best grand national awards of scientific progress in 2004 and 2014, respectively. The pedigree relationships among these cultivars were shown in Supplementary Fig. S1^9^,^10^,^11^. This has provided us with very valuable information to reveal the formation and diversification of founder genotypes in historical wheat breeding in China.

In this study, a Chinese wheat mini core collection^12^, diploid collections of *T. boeoticum, T. uratu, T. monococcum*, and tetraploid wheats of *T. dicoccoides, T. dicoccum, T. durum*, etc., were genotyped using the wheat 9 K iSelect SNP array^13^. The SNP marker-based haplotype blocks were developed and evaluated. Association analysis was carried out in common wheat using agronomic trait data collected in four environments and their meta data. We found detection power was much greater when performing association based on haplotypes than on the use of single SNP markers. We also found that the revolutionary differentiation in both the A and the B genomes were induced by both tetraplodization and hexaploidization during wheat evolution. This study also tracked the inheritance of haplotype blocks among common wheat cultivars of shared pedigree, and their formation in tetraploid and hexaploid in the A and the B genomes. We revealed the patterns of haplotype blocks in the founder genotypes and their impacts on the wheat breeding in China at different periods during the last century. Specifically, the critical genomic regions controlling agronomic traits had stronger and larger haplotype blocks likely resulted from the conscious selection in both cultivated tetraploid and hexaploid wheat, but generally these blocks were formed earlier in the A genome than in the B genome. We also found the A genome experienced stronger selection than the B genome in both domestication and breeding.

## Results

### Genome and chromosome distribution of the 9 K SNP markers

Genotyping of the Chinese wheat mini core collections yielded a total of 5756 polymorphic SNPs, of which 5156 were mapped to a single chromosome location (Supplementary Table S1). Among them, 2420 were on the A genome chromosomes, 2396 on the B genome chromosomes, but the D genome has the fewest, only 340 (Supplementary Table S2). Therefore, the analysis of haplotype blocks, haplotype-based association and evolution performed in this study, focused on the SNPs located on the A and the B genomes. Principle component analysis (PCA) and population structure analysis based on the entire set of 5756 markers generally separated the landraces from the modern cultivars into two subpopulations, although some landraces and modern cultivars can not be assigned to either of the subpopulations likely due to admixture (Supplementary Fig. S2). Mean gene diversity values of the A genome were 0.079, 0.318, 0.229 and 0.347 for diploids, tetraploids, landraces, and modern cultivars, respectively, while they were 0.117, 0.326, 0.248, and 0.349, respectively, on the B genome (Supplementary Table S3), indicating that diversity in the gene coding regions was higher in the B genome compared with the A genome, and modern cultivars appeared to be more diverse than landraces.

### In *Triticum* genus, two steps of polyplodization induced revolutionary differentiation in both the A and the B genomes

**Figure 1:**
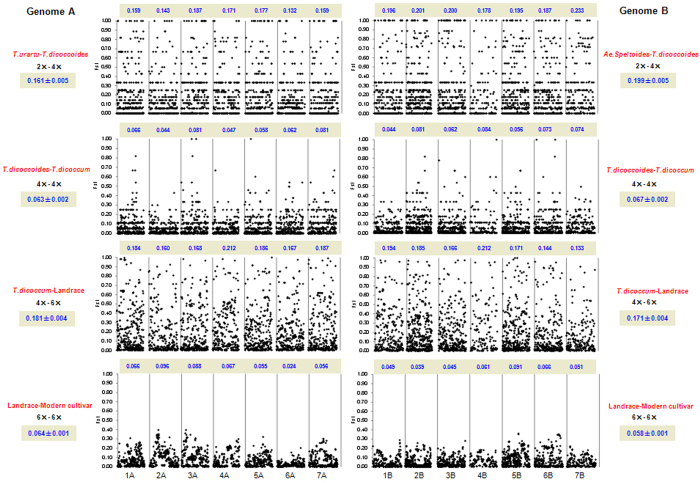
Diversification index (*Fst*) between species, the modern cultivars and landraces of common wheat, which were estimated based on the effective SNPs in the A and the B genome respectively. The black dots mean *F*_*st*_ for each SNP marker. The *F*_*st*_ between species was given at the right and left for the A and B genome respectively. For each chromosome, they were given at the top of each chromosome. It is very clear that tetraploidization and hexaploidization induced much stronger diversification than either domestication of tetraploid and modern breeding of common wheat. But the effect of tetraploid domestication is much stronger than that of breeding for diversification within A or B genome in common wheat.

We employed differentiation index (*Fst*), PCA, and neighbour-joint tree analyses to show the genetic differentiation between species and populations in the same species. The *Fst* values between *T. dicoccum* and *T. dicoccoides* were 0.063 and 0.067 for the A and the B genomes, respectively. However, these values were three times higher when comparing tetraploid wheats *T. dicoccoides* either with *T. uratu* or with *Aegilops speltoides* at 0.161 and 0.199, respectively. Furthermore, three times higher differentiation was found between *T. aestivum* landrace and *T. dicoccum* than between hexaploid modern cultivar and landrace on both the A and the B genomes. The *Fst* values were 0.181 vs. 0.064, and 0.171 vs. 0.058 for the A and the B genomes, respectively (Fig. 1). When comparing genetic differentiation between the domesticated tetraploids and common wheat landraces, higher gene diversity was observed after the occurrance of polyploidization in the gene coding regions (Fig. 1; Supplementary Fig. S3).

**Figure 2:**
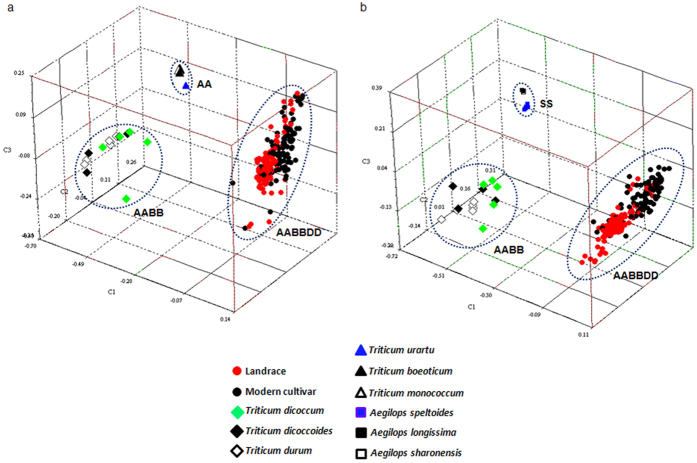
PCA analysis of variant species, landraces and modern cultivars based on the SNPs in the A (**a**) and the B (**b**) genome, respectively. Unlike the expected, the gene diversification in both diploid wheat and *Aegilops Sitopsis* species are very low, but in both tetraploid and hexaploid wheat, the gene diversity is much higher than in diploid species.

Results from PCA showed three distinct subgroups each corresponding to the diploid, tetraploid, and hexaploid wheats (Fig. 2). Higher gene diversity was detected in both the tetraploid wheat and common wheat, in contrast, gene diversity was much lower in the diploid species, *T. boeoticum, T. monococcum* and *T. urartu*, as well as in the *Ae. speltoides, Ae. sharonesis, Ae. longissima* and *Ae. searsii* (Fig. 2). This implied that tetraplodization and hexaplodization may have dramatically increased the gene diversity in both the A and the B genomes. This was further supported by neighbour-joint trees based on the SNPs within the A and the B genomes (Supplementary Fig. S3). In the tree constructed based on the A-genome specific SNPs, the diploids were separated into two sub-groups, i.e. the subgroup containing predominantly *T. urartu* and the subgroup containing *T. boeoticum* and *T. monococcum*, while the tetraploids were clustered together in a separate cluster. When the tree was constructed based on the B-genome specific SNPs, *Ae. speltoides* collections were clustered into a subgroup, and separated from a subgroup containing the collections of other *Sitopsis* species, with tetraploids clustered in a separate cluster from the diploids. The cluster separation was distinct among diploids, tetraploids, and hexaploid.

### The A genome has larger but fewer blocks than the B genome

**Figure 3:**
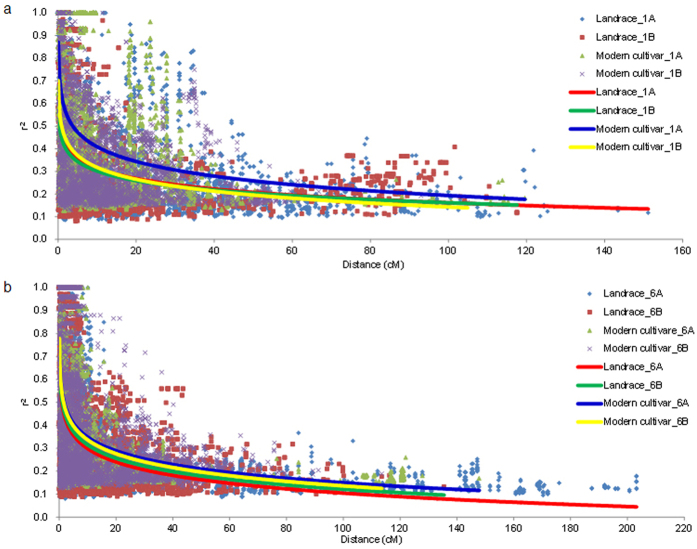
LD analysis for 1A vs 1B (**a**), 6A vs 6B (**b**) in landraces and modern cultivars respectively in MCC. The LD curves clearly indicate that previous selection to 1A and 6A were stronger than to 1B and 6B in modern breeding though there were no obvious difference in the landrace.

**Figure 4:**
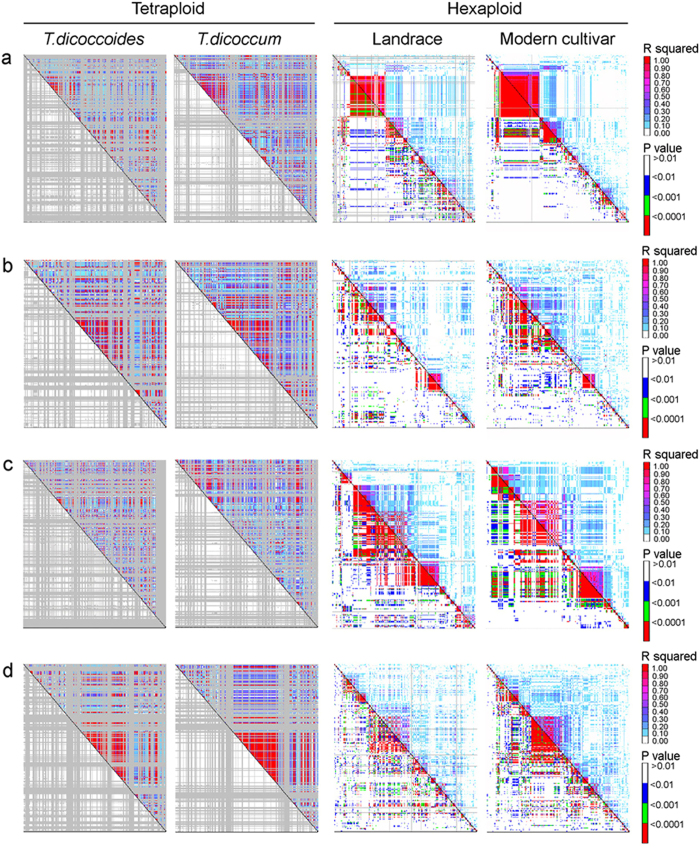
Comparison for haplotype blocks on 1A (**a**) vs 1B (**b**), 6A (**c**) vs 6B (**d**) in *Triticum dicoccoides, T. dicoccucum, T. aestivum* cv. landraces and modern cultivars. The haplotype block formed evolutionary earlier and stronger on 1A and 6A than on 1B and 6B respectively, which was refelcted by *r*^*2*^ and *p* values.

Total 878 haplotype blocks were detected in the A and the B genomes of common wheat, of which 406 were on the A genome, 472 on the B genome (Table 1; Supplementary Table S4; Supplementary Fig. S4). About 92.4% of the SNPs were involved in developing these haplotype blocks, indicating a good block coverage on both the A and the B genome chromosomes (Table 1). The sizes of the blocks were larger on the A genome chromosomes than on the B genome chromosomes, with an average 1.23 and 0.96 cM obeserved, respectively. The block sizes on the B genome tended to be more variable than those on the A genome (Supplementary Table S5). The largest blocks appeared on chromosomes 1A, 2A, 3A, 4A, 4B, 5B and 6A. This was likely attributed to stronger artificial selection occurring on the A genome than on the B genome. The LD value difference further supported this interpretation (Supplementary Fig. S5). In the modern cultivars, the A genome generally had higher LD than the B genome, as indicated by an example showing slower LD decay on chromosome 1A as opposed to chromosome 1B, and similarly for chromosome 6A compared to chromosome 6B (Fig. 3). The sizes and distributions of the blocks on each chromosome, such as 1A vs. 1B, 6A vs. 6B, also supports the notion that selection pressure was stronger on the A genome than on the B genome during the process of domestication and breeding (Fig. 4; Supplementary Fig. S6).

### Intense human selection caused strong haplotype blocks, but artificial crosses in breeding promoted effective recombination of genes

**Figure 5:**
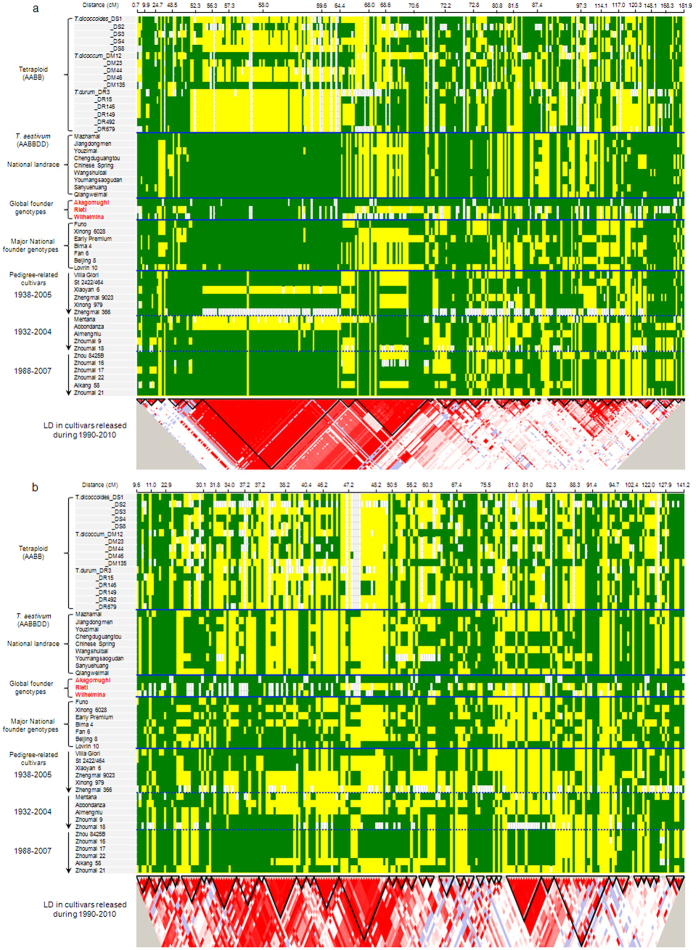
Formation and evolution of haplotype block on chromosomes 1A (**a**) and 1B (**b**) in one century of breeding. All SNP alleles in Akagomughi were assigned as green color. For other cultivars or collections, different alleles from Akagomughi were assigned as yellow, the missing SNP allele by white color. The figure at the bottom was haplotype block map made by Haploview 4.2 software in the newly released cultivars (1990–2010) in China based on SNP markers.

We observed a large haplotype block (about 15.9 cM) on the short arm of chromosome 1A spanning a region between 48.5 and 64.4 cM, that was present in the founder parents and was maintained in most of the newly released Chinese cultivars (Fig. 5a). The well-known Chinese landraces, such as Chinese Spring (Chengdu, Sichuan), Jiangdongmen (Nanjing, Jiangsu), Youzimai (Qingfeng, Henan), and Sanyuehuang (Linying, Henan), etc., all shared the same allele composition present in the block as found in Akagomughi (depicted as green color in Fig. 5a), whereas two blocks with contrasting allele compositions were found in modern cultivars (depicted as green and yellow colors in Fig. 5a), after more than one hundred years of breeding process. Two additional founder parents, Rieti and Wilhelmina, were also found sharing the same allele composition in the block as Akagomughi, referred to as Block A. These three founders have been widely used in wheat breeding worldwide. Therefore, the formation of the block with the alternate allele composition, referred to as Block B, was likely the result of strong breeding pressure. The other founder genotypes introduced from Europe, such as Mentana and Abbondanza, had Block B type, and was different from Vila Glori and St 2422/464 that had Block A type. The more recent founder, Zhou 8425B, and its derived cultivars all had Block A type. In contrast, the other more recent founder, Xiaoyan 6, had Block B with the break point located at 52.3 cM position, but the haplotype block in its derived cultivars, Zhengmai 9023 and Xinong 979, was replaced by Block A type (Fig. 5a).

Results further indicated that these two large blocks on chromosome 1A were also present in the tetraploid species, particularly *T. dicoccum* and *T. durum* (Fig. 5a). The overall haplotype block distribution on chromosome 1A was more variable in tetraploid species than those in hexaploid wheat. This suggested that large haplotype blocks already existed in the tetraploid species, and that strong selection favored the A type block as found in the landrace, founder genotypes and released cultivars of common wheat. Similar selection bias was also obvious on chromosomes 3A, 4A, 6A, 7A (Supplementary Fig. S7). However, for the B genome chromosomes, except for chromosomes 2B and 5B, most of the larger haplotype blocks were detected in *T. aestivum* but not in *T. dicoccum* or *T. durum* (Fig. 5b; Supplementary Fig. S7). This suggested a more recent but weaker selection pressure applied to most of the B-genome chromosomes. On chromosomes 2B and 5B, stronger and larger blocks were detected in tetraploid wheat, and were passed on to common wheat (Supplementary Fig. S7). This implied that chromosomes 2B and 5B may be critical for wheat domestication, which is consistent with the finding from our previous study based on SSR markers in the mini core collections^14^.

Small but obvious haplotype blocks were present on the short arm of chromosome 1B in the landraces, but they were broken and no longer maintained in the national founder genotypes and historical pedigree-related cultivars derived from Villa Glori and St 2422–464 (Fig. 5b). A few noticeable blocks at 11.0–91.4 cM were found in St 2422/464 and its derivative Xiaoyan 6 (released in 1981). But new blocks were created in Zhengmai 9023 and Xinong 979 (both released after 2000) as a result of crossing Xiaoyan 6 with other cultivars. Introduction and the wide use of 1BL/1RS translocation has dramatically fixed the haplotype block into two major types, i.e. Zhou 8425B (such as Zhoumai 9, Zhoumai 18, Zhoumai 16, Zhoumai 17, Zhoumai 22, etc.), and Aimengniu. Blocks found in Zhoumai 21 and Aikang 58 were likely the result of exchanges between the Zhou 8425B type and the Aimengniu type. From comparing the results revealed between chromosomes 1B and 1A, the main conclusion can be drawn was that the selection pressure to the chromosome 1B was likely weaker than that to the chromosome 1A in the early stages of domestication of tetraploid and hexaploid wheats.

On chromosome 6A, a very large haplotype block in a size about 94.3 cM was detected near the centromere region in common wheat (Supplementary Fig. S7). The three widely used founder parents, Rieti, Wilhelmina, and Akagomughi had two blocks with contrasting allele compositions located in the region between 45.7 and 140 cM on chromosome 6A with one type represented by Akagomughi, and the other by Rieti and Wilhelmina (Supplementary Fig. S7). Funo inherited the Akagomughi type, while Villa Glori shared the Wilhelmina type. Multiple recombination events occurred in Mentana at this region. The block in St 2422/464, however, was likely the result of exchange between Akagomughi and Wilhelmina (also Rieti) types at the 83.3 cM region. In Xiaoyan 6, a well-known cultivar derived from St 2422/464 from a cross with Xiaoyan 96, this region between 45.7 and 83.3 cM was reverted back to the Akagomughi type, with four recombination sites at 83.3, 89.7, 117.0, and 135.0 cM, and was fully passed on to Zhengmai 9023, Xinong 979 and Zhengmai 366 (Supplementary Fig. S7).

The block type present in Villa Glori was also found in Early Premium, Lovrin 10, and Aimengniu, the three founder genotypes that were widely used in the 1950 s, 1980 s, and 1990 s in China. Frequent recombination events detected in Zhou 8425B, a more recent founder genotype created at the end of last century, have quickly reverted the block back to a Akagomughi-like type at the region between 45.7 and 89.9 cM. Both of the two block types described were also present in Chinese landraces. However, only small portions of these two block types were detected in *T. durum* and a few *T. dicoccum* accessions, but none in *T. dicoccoides* (Supplementary Fig. S7).

We observed two large haplotype blocks on chromosome 6B in modern cultivars of common wheat (Supplementary Fig. S7, marked by the red square) located in the regions between 63 cM and 82 cM and between 86 cM and 96 cM, with the latter also found in the landraces. Both of the two blocks were maintained in all the newly released cultivars. Portions of the blocks were present in *T. durum*, but they were not visible in *T. dicoccum* and *T. dicocoides*. Similar findings were found in 10 other chromosomes, where large blocks were detected in both *T. durum* and *T. aestivum* cultivars (Supplementary Fig. S7).

Even though the haplotype blocks very conserved from 52.3 to 64.4 cM on chromosome 1A between the modern cultivars and the landraces, we could not ignore that many recombinants occurred in modern breeding from 72.2 to 148.1 cM (Fig. 5). Similar phenomenon were detected on most of chromosomes in the A and B genomes (Supplementary Fig. S7). Therefore, it could conclude that intense human selection caused strong haplotype blocks at the crucial genomic regions, but artificial crosses in breeding promoted effective recombinations of genes in other regions.

### Haplotype-based association had higher detection power than single SNP markers

Using phenotypic data collected for 11 yield related traits evaluated in four environments from the mini core collections, we performed association studies (GWAS) based on single SNP markers and haplotypes separately. From the 5,156 SNPs that mapped on single chromosome location, 4,434 polymorphic SNPs with minor allele frequency (MAF) ≥ 0.05 were chosen for GWAS. The mean value for each trait was calculated using BLUP. SNP-based association analysis identified 234 loci (806 association signals) associated with traits at *P* < 2.3 × 10^−4^, and they were distributed on all chromosomes except 4D and 7D (Supplementary Tables S6,S7,S8; Supplementary Figs S8–S18). Among them, 105 (44.9%) loci were associated with two or more phenotypic traits. The chromosome 3B has the largest number of trait-associated SNPs (17, 77.3%). Additionally, 70 loci associated with the same traits were consistently detected in at least two environments and the remaining were significant in only one environment.

A total of 878 haplotype blocks were detected on the A and the B genome chromosomes, and 847 of them with MAF ≥ 0.05 were selected for GWAS. Total 123 blocks (192 association signals) were found associated with yield-related traits at *P* < 1.2 × 10^−3^, distributing on all the A and the B genome chromosomes (Supplementary Tables S9,S10,S11; Supplementary Figs S19–S29). Favorable haplotypes of 160 associations with specific traits were identified showing large genetic effects. Among the blocks associated with evaluated traits, 21 (17.1%) were associated with at least two traits, and 14 were associated with the same traits expressed in at least two environments.

**Figure 6:**
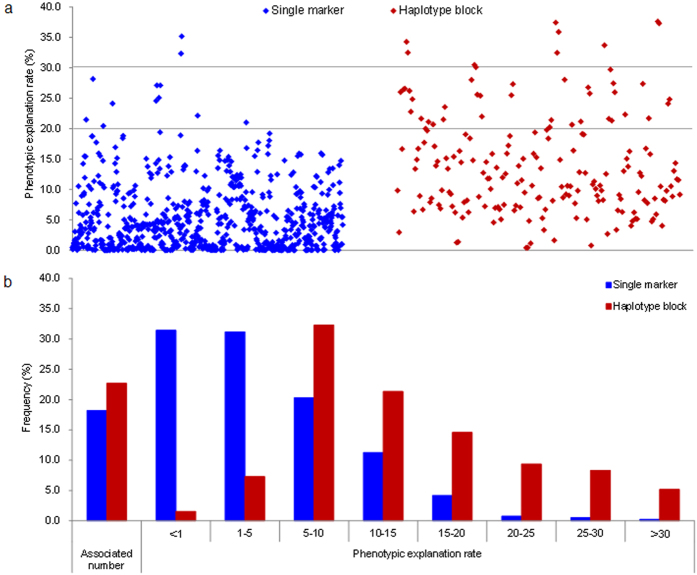
Detection power comparison of associations based on single SNP markers and haplotype blocks. (**a**) Dot distribution of phenotype explanation rate (%) for overall association signals in both SNP-based association and haplotype-based association. (**b**) Frequency distribution of different groups of phenotype explanation rate (%) for overall association signals in both SNP-based association and haplotype-based association.

**Figure 7:**
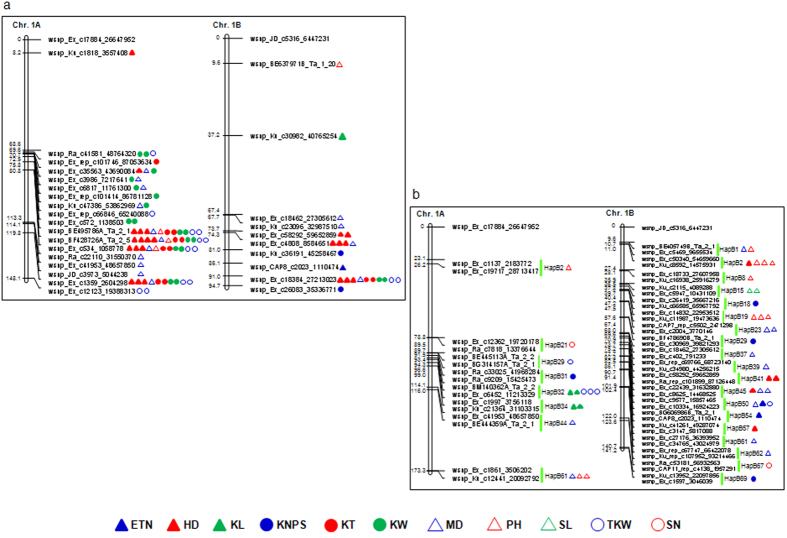
Effectiveness comparison of single SNP-based association (**a**) with haplotype-based association (**b**) in chromosomes 1A and 1B. Haplotype blocks dramatically increased the sensitiveness and power of association, especially on genomic regions experiencd very intensive human selections, such as the short arm of chromosome 1B.

The use of haplotype was able to increase the power of associations and the amount of phenotypic variation explained (Fig. 6), as indicated by the results from chromosome 1B (Fig. 7). However, it was not the case for chromosome 1A, this was probably due to the presence of a large block encompassing the entire short arm and the proximal of the long arm likely resulted from an artificial selection originated from the tetraploid species and persisted in the modern cultivars of common wheat (Figs 5a and 7; Supplementary Fig. S30). Similar findings of large blocks were also observed on the proximal regions of both arms on chromosome 6A, but haplotype-based association improved the detection power in the genomic regions (Supplementary Figs. S7, 30), where major and stable QTLs influencing yield traits were previously detected using DH populations and association mapping approaches based on SSR markers^15^,^16^.

## Discussion

Polyploidy has been important in the evolution of angiosperms and may significantly affect population genetic diversity and structure. The common wheat was formed through two steps of polyploidization, allotetraploidization (approximately 0.5 million years ago) and hexaploidization (approximately 10,000 years ago)^17^. The revolutinary changes included elimination of non-coding sequences from one of the two homoeologous pairs in tetraploids and from two homoeologous pairs in hexaploids, and resulted in augmenting the differentiation of homoeologous chromosomes at the polyploid level, thus providing the physical basis for the diploid-like meiotic behavior of allopolyploid wheat^18^,^19^. In this research we found that both tetraploid and hexaploid polyploidization have significantly increased gene diversity, as demonstrated by the change of gene diversity among populations (Supplementary Table S3) revealed by PCA (Fig. 2), haplotype block, and dendrogram tree analyses using the A or the B-genome specific SNPs (Fig. 5; Supplementary Figs S3 and 7). Generally, bottleneck effect during polyploidization caused significant reduction of diversity on whole genome^20^, which masked the increase of diversity at coding regions caused by genome shock and adaptation between genomes in the neo polyploid species. However, breeding selection usually but not always caused reduction of diversity, such as *Glu-1*^21^ and *PPd1*^22^, breeding dramatically increased the diversity. As a strict self-pollination crop, domestication and farmer cultivation accumulated large amount variations, but these variations mainly concentrated at inter-genic and promoter regions rather than in the coding regions, especially exons. Artificial crosses promoted effective recombination between loci as well as alleles within one locus, which might raise the diversity except the locus (loci) strongly targeted in breeding selection.

In addition, modern breeding also raised the diversity in gene coding regions, which implies that artificial hybridization and selection have an effect to equalize frequency of alleles (SNPs) at the same locus, otherwise, strong selection in breeding will lead to declining of gene diversity at most loci. Though intensive selection increased the LD (*r*^*2*^) on the entire genomes (Figs 3 and 4), we can’t ignore the fact that breeding has significantly raised gene diversity at distal regions through introducing variations and increasing the effective recombinations on most of chromosomes (Fig. 5; Supplementary Fig. S7). This might also be related to the facts that most of genes controlling biotic- and abiotic-stress usually distribute at the distal regions^23^,^24^,^25^, which have more chances to create new alleles through a higher frequency of recombination.

It is widely accepted that domestication and breeding usually caused a dramatic reduction on genetic diversity known as two step genetic bottlenecks^3^,^20^,^26^. From screening 512 SSR markers on the mini core, we found that landraces and modern cultivars were grouped in two relatively separated subpopulations, and the landraces had higher genetic diversity^14^. However, in the current research, sliding window of haplotype block analysis clearly showed that morden cultivars had a higher gene diversity than landraces in the gene coding regions (Supplementary Table S3; Supplementary Fig. S31), i.e. modern breeding globally elevated the gene diversity of coding regions in the A and the B genomes. This was also supported by the neighbour joint trees based on the SNP markers in the A and the B genomes (Supplementary Fig. S3), and was consistent with what was reported by Cavanagh *et al*.^13^. So we wondered what could be the reason for the inconsistency. Generally in crops, about 20% of genes in the genomes have undergone human selection, and 80% were neutral genes^4^,^27^. Selection often leads to a differential loss of genetic diversity in targeted genomic regions, but with great possibility to enhance diversity of neutral genes through promoting gene flow, recombinations and exchanges between populations or ecotypes (Supplementary Table S3; Supplementary Fig. S31)^28^. Of course, we can’t ignore another possibility that all of the 9 K SNPs were developed based on transcriptome sequencing of common wheat^13^, variations in promoter regions and introns were not surveyed, where the major variations for functional genes between collections are present^29^,^30^.

In both A and B genomes, LD was higher in the modern cultivars than in the landraces. In addition, selection pressure to the A genome was stronger than to the B genome (Fig. 3). This might be because agronomic traits tended to be the selection targets, and the A genome generally carries more genes controlling known agronomic traits than the B genome in wheat. The numbers of both trait QTLs and genes affecting domestication in the A genome significantly exceeded those in the B genome^31^. Large blocks formed earlier and stayed intact in the A genome than in the B genome on the two homoeologous chromosomes studied (Fig. 4). Similar asymmetrical selection pressure on the subgenomes A and C in domestication and breeding was also found in *Brassica napus*^32^.

SNP is becoming one of the most popular marker systems employed in association mapping for being highly abundant in the genomes and for its high throughput analysis capabilities. It is not surprising that millions of SNPs can be discovered within a single crop after resequencing hundreds of collections. However, much fewer genomic regions were found associated with agronomic traits than expected. Most of the studies published in the past years mainly showed the SNPs significantly associated with genes previously identified to validate their association results. This might be due to the biallelic characteristic of SNP markers. The use of haplotypes containing a group of SNP markers can improve the levels of polymorphisms and should help overcome the drawback of using single SNP markers by creating much more combinations (haplotypes) (Supplementary Table S4). This can dramatically increase the power and the effectiveness of haplotype-based association^33^,^34^. In the current study, we detected a haplotype (HapB-1A-29-2) positively increased TKW by 6.82 g, and an associated trait for kernel numbers per spike (KN) next to this haplotype further confirmed the result. However, no single SNP markers associated with TKW were identified in this region (Fig. 7; Supplementary Table S6). Additionally, the HapB-7A-75-3 at the distal region of chromosome 7AL was positively associated with KN by increasing 3.20 kernels, but no associations were found in the same trait when using single SNP-based association in the corresponding region (Supplementary Table S6), where genes influencing grain number were detected in 12 NILs involving *Lr19* translocation lines (7Ag/7A) in various genetic backgrounds evaluated in multiple environments^35^. For simple trait, such as plant height (PH), the effectiveness of using haplotype-based association was more prominent. The genomic regions between two blocks HapB-4B-13 and HapB-4B-17 near 68.33 cM and 73.47 cM were repeatedly detected having association with the PH trait in multi-environments, but there was only one single SNP signal detected in one environment (Supplementary Table S6).

Modern plant breeding has promoted gene exchanges and recombination between parents used for crossing. Hence the activities can enhance the gene diversity in modern cultivars. This was strongly supported by comparing genetic diversity using haplotype blocks between homoeologous chromosome pairs on the A and the B genomes (Supplementary Fig. S31). However, we also observed some genomic regions that were not affected by the breeding efforts, such as a 51.1 to 72.5 cM region on chromosome 1A, a 12.4 to 66.2 cM region on chromosome 5A, and a 52.6 to 117.0 cM region on chromosome 6A. These regions contained large haplotype blocks, and were likely subjected to selection during domestication and breeding improvement soon after wheat was domesticated. They could be the crucial regions impacted by both domestication and breeding, and have been fixed in the landrace. These regions may harbor important candidate genes associated with domestication, but may offer little use for breeding improvement, because these regions have already been fixed in all the cultivars (Fig. 5; Supplementary Fig. S7). Further example to corroborate the above conclusion was the high levels of genetic differentiation occurred for genes on chromosomes 1AL and 5AL (Supplementary Fig. S31). Of the 14 chromosomes studied, 3A, 4B and 5B showed the highest block diversity between modern cultivars and landraces.

Many introgression, NAM, and MAGIC populations have been developed in major crops, such as rice, maize, wheat and oilseed, where elite cultivars were usually used as the major genetic background in the crossing design^36^,^37^. These populations were efficiently used in allele minining via association mapping and QTL analysis. In self pollinated crops, even in maize inbed lines, each chromosome can be dissected into major haplotype blocks (Fig. 5; Supplementary Fig. S7; Supplementary Table S4)^38^,^39^, Tag-SNP markers can be selected to track the variants in each block. Based on haplotype association, we can estimate breeding value for the block variants and pyramid the favorable ones through intercrossing lines within a population, assisted by Tag-SNP marker selection in the segregating populations. This may provide a new strategy used for genome selection in breeding in the coming years^37^,^39^. Shifting from using single marker assisted selection to using multiple genomic block selection would be the next breakthrough for molecular breeding to create ideal genotypes.

## Materials and Methods

### Plant materials

A Chinese wheat mini core collection (MCC)^10^,^12^ was used for association analysis of yield traits using both single markers and haplotypes, as well as for the construction of haplotype block maps on the A and the B genome chromosomes. The MCC contains 262 wheat accessions, including 157 Chinese landraces, 88 Chinese modern cultivars, and 17 introduced lines. They represent 1% of the national collections but more than 70% of the entire genetic diversity^12^,^14^. Furthermore, 184 cultivars released from 1990 to 2010 in China were also used for detecting haplotype blocks. A complete information of common wheat materials used in this study were listed in Supplementary Table S12.

To analyze gene differentiation and haplotype block between possible progenitors and common wheat on the A and the B genome chromosomes, additional 27 dipolid wheat collections, including 6 *Triticum urartu*, 4 *T. boeoticum*, 4 *T. monococcum*, 6 *Aegilops speltoides*, 3 *Ae. longissima*, 3 *Ae. sharonensis* and 1 *Ae. searsii*, and 16 tetraploid wheats, including 5 *T. dicoccoides*, 5 *T. dicoccum*, and 6 *T. durum*, were also used in the current study (Supplementary Table S12).

Furthermore, 20 cultivars derived from founder genotypes Zhou 8425B and Akagomughi were employed to investigate the inheritance of haplotype blocks among accessions sharing similar pedigree relationship on individual chromosome of the A, and the B genomes (Supplementary Fig. S1; Supplementary Table S12).

### Phenotypic assessment

During the 2001–2002, 2004–2005, and 2005–2006 wheat-growing seasons, the MCC accessions were planted at the Chinese Academy of Agricultural Sciences (CAAS)-Luoyang Experiment Station in Henan province (111.8°E, 33.4°N), and at the CAAS-Shunyi Experiment Station in Shunyi, Beijing (116.3°E, 40.0°N) for the 2009–2010 growing season. Each accession was planted in a 2 m four-row plot with 30 cm between rows, with 40 seeds planted in each row. The filed management followed local practices.

Data from yield traits of all accessions in the MCC were collected from ten plants selected from the middle of each plot. These traits included heading date (HD, days), maturity date (MD, days), spike length (SL, cm), spikelet number per spike (SN, number), plant height (PH, cm), kernel numbers per spike (KN, number), effective tiller number (ETN, number), 1000-kernel weight (TKW, g), kernel length (KL, mm), kernel width (KW, mm), and kernel thickness (KT, mm). The data were collected in all four environments, i.e. in 2002, 2005, and 2006 at Luoyang, Henan province, and in 2010 at Shunyi, Beijing; they were named as 02LY, 05LY, 06LY, and 10SY, respectively. The detailed phenotyping data are listed in Supplementary Table S13.

### DNA extraction

Genomic DNA was isolated from 100 mg fresh leaves of 10-day-old seedlings of each accession using DNA quick Plant System by Tiangen Biotech (Beijing) Co., Ltd (www.tiangen.com) according to the manufacturer’s instructions (Tiangen). The DNA concentration was measured and diluted to approximately 50 ng/uL for SNP genotyping.

### SNP genotyping

The recently developed high-density wheat iSelect 9 K SNP chip comprised of approximately 8,632 gene-associated SNPs^13^ was used to generate genotyping data for polyploid wheat and to characterize genetic variations among different gene pools. The iSelect SNP genotyping was performed using Infinium assay and iScan instruments according to the manufacturer’s protocols (Illumina). The genotyping work was performed in US Department of Agriculture-Agricultural Research Service genotyping Lab, Fargo, ND. SNP allele clustering and genotype calling for hexaploid wheat selected in this study were performed with GenomeStudio (GS) v2011.1 software (Illumina). The accuracy for SNP clustering was visually checked. The final data set contained 5,156 polymorphic SNPs that were mapped to a single chromosome location based on the consensus map information^13^. Details for chromosomal locations and their genetic positions (cM) of the final polymorphic 5156 SNPs were listed in Supplementary Table S1. As described in Cavanagh *et al*.^13^, the number of SNP markers distributed on the D genome was much fewer than that on the A and the B genomes, and some D genome chromosomes, including 3D, 5D, 6D and 7D, had at least two linkage maps that were not able to make further relative calculations. Therefore, analyses of haplotype blocks in this study were focused on the A and the B genomes. The detailed information of genotyping data for different wheat genetic resources are listed in Supplementary Table S14.

### Data analyses

#### Phenotypic traits assessment

Basic statistics, analysis of variance (One-Way ANOVA) according to Tukey test at the significance level of 5% (*P* ≤ 0.05) between populations, and Pearson correlation analysis between traits within a single environment and between environments for a trait were analyzed using SPSS 16.0 software (http://www.brothersoft.com/downloads/spss-16.html). The mixed mean value of each trait from multiple environments in the MCC was estimated by the best linear unbiased predictor (BLUP) method according to Bernardo^40^,^41^,^42^. Based on the procedures of the TASSEL v3.0 software (http://www.maizegenetics.net/)^43^,^44^, using only phenotypic data and the kinship matrix, the heritability (*h*^*2*^) of each test trait in different environments, defined as the proportion of genetic variance over the total variance, was also calculated according to the formula *h*^*2*^ = σ_a_^2^/(σ_a_^2^ + σ_e_^2^) with the MLM options of no compression and re-estimation for each marker. Here, σ_a_^2^ means genetic variance, and σ_e_^2^ indicates the residual variance.

#### Diversity, genetic differentiation and dendrogram tree analyses

Standard statistics for genetic diversity at each SNP locus, including polymorphism information content (PIC value) and gene diversity, were carried out using PowerMarker v3.25 software^45^. The *F*_*st*_ (coefficient of gene differentiation) was employed to measure genetic differentiation between populations using POPGENE software^46^. And finally, genetic dissimilarities between accessions were calculated using the simple matching coefficient in DARwin v5 software^47^. Cluster analysis and a neighbor joining tree construction were performed based on the Manhattan dissimilarity matrices with the un-weighted pair-group method using arithmetic averages (UPGMA). Principal coordinate analysis was also used to reveal the relationships among different populations based on the above dissimilarity matrices using NTSYS-pc v2.1 software^48^.

#### Genetic structure evaluation in the test genotypes

In order to reduce the false positive or spurious associations, genetic structure in the population level (Q values) was evaluated uisng STRUCTURE v2.2 software^49^ with a burn-in period at 50,000 iterations and a run of 500,000 replications of Markov Chain Monte Carlo (MCMC) after burn in. For each run, 5 independent runs were performed with the number of cluster K varying from 1 to 11, leading to 55 Structure outputs. Then the number of populations was estimated on the basis of the Evanno criterion^50^. Additionally, the degree of genetic covariance between pairs of individuals, the relative kinship matrix (K values) was calculated using SPAGeDi software^51^. Calculation of pairwise kinship coefficients was according to Loiselle *et al*.^52^ with 10,000 permutation tests. Negative values between individual pairs were changed to 0, as this indicated a lower than expected relationship between two random individuals^53^.

#### Haplotype block mapping and diversity analysis

Haplotype structure in wheat A and B genome chromosomes was evaluated using Haploview 4.2 software package (http://www.broadinstitute.org/haploview/haploview)^54^. The package defines haplotype blocks, provides the number of haplotypes and their genetic length (cM) for each block, as well as the number of tag SNPs based on solid spine of linkage disequilibrium (LD) (Extend spine if D′ > 0.8). In brief, this meant that the first and the last marker in a block were in strong LD with the intermediate markers that were not necessary in LD with each other^54^. In the yield traits association studies, LD statistics and the haplotype patterns of the Chinese MCC population were calculated using the entire genotype data on each chromosome in a graphical interface.

Gene diversity and coefficient of gene differentiation (*F*_*st*_) within and between landraces and modern cultivars were further evaluated by treating one haplotype as a single marker on individual chromosome.

To simplify formation and transmission analysis of haplotype blocks in different wheat genotypes, 3660 SNPs with a minor allele frequency more than 0.05 in all populations were selected for this study (Supplementary Table S1). Graphically all the SNP alleles from the founder parent Akagomughi were denoted as green color. For other cultivars or collections, if they carried the same SNP alleles as Akagomughi, they were also shown in green color, otherwise, they were in yellow. The missing SNP alleles were expressed as white color.

#### Sliding window analysis of gene diversity in haplotype blocks

To graphically display population differentiation on the A and the B genomes between Chinese landraces and modern cultivars, the diversity index was calculated based on a three-block sliding window. This was done based on the mean distance between adjacent blocks, haplotype windows including three blocks were shifted from the short arm of each chromosome to the long arm one block at a time using a visual basic language-based program in Excel.

#### Single SNP-based and haplotype-based GWAS on yield-related traits

The markers with the minor allele frequencies less than 0.05 were treated as rare alleles and filtered out. The compressed mixed linear model (MLM)^44^,^53^ including Q + K in the model implemented in the TASSEL v3.0 software package (http://www.maizegenetics.net/)^43^,^44^ was used to identify genome-wide association signals between SNP markers/haplotypes and yield traits in the Chinese MCC. The MLM options of optimum level and population parameters previously determined (P3D) were applied in the study. Based on the numbrer of markers (n) with minor allele frequency (MAF) more than 0.05, significant association (*P* < 1/n) was declared at a *P* value threshold *P* < 2.3 × 10^−4^ (−Log*P* > 3.65) and *P* < 1.2 × 10^−3^ (−Log*P* > 2.93) for SNP markers and haplotypes, respectively.

Additionally, phenotypic variation explaned (*R*^*2*^) by the markers was calculated using ANOVA function in SPSS 16.0 software, based on a formula *R*^*2*^ = SSA/SST where SSA being the sum of squares between groups, and SST being the total sum of squares^44^,^55^. Furthermore, favorable allele at each associated locus was determined by evaluating and comparing the genetic effects of the alleles using the mean value of the population. Finally, genetic maps containing all the associated SNPs and haplotype blocks associated with the yield traits were drawn using MapDraw 2.1 software (a plug-in of Excel program) according to the consensus map information^13^.

## Additional Information

**How to cite this article:** Hao, C. *et al*. The iSelect 9K SNP analysis revealed polyploidization induced revolutionary changes and intense human selection causing strong haplotype blocks in wheat. *Sci. Rep.* **7**, 41247; doi: 10.1038/srep41247 (2017).

**Publisher's note:** Springer Nature remains neutral with regard to jurisdictional claims in published maps and institutional affiliations.

## Supplementary Material

Supplementary Information

Supplementary Table S1

Supplementary Table S4

Supplementary Table S6

Supplementary Table S9

Supplementary Table S12

Supplementary Table S13

Supplementary Table S14

## Figures and Tables

**Table 1:** Number of SNPs, haplotypes and haplotype blocks on each chromosome in the A and B genomes of common wheat.

Chr.	SNP					HapB			Hap		
	Total SNPs	SNPs in HapB (%)	Range	Tag SNPs (%)	Range	Total HapBs	Mean (cM)	Range (cM)	Total Haps	Mean	Range
1A	419	392 (93.6)	2–49	187 (44.6)	1–6	65	1.01	0–9.42	290	4.46	2–9
1B	295	275 (93.2)	2–20	177 (60.0)	1–8	69	0.69	0–5.31	284	4.12	2–11
2A	304	281 (92.4)	2–34	134 (44.1)	1–9	52	1.29	0–10.72	220	4.23	2–13
2B	529	491 (92.8)	2–37	261 (49.3)	1–12	96	1.12	0–8.71	410	4.27	2–25
3A	340	320 (94.1)	2–23	162 (47.6)	1–11	53	1.35	0–12.08	232	4.38	2–12
3B	373	328 (87.9)	2–24	209 (56.0)	0–8	79	0.69	0–5.86	332	4.20	1–10
4A	315	287 (91.1)	2–48	117 (37.1)	1–7	43	1.84	0–11.45	168	3.91	2–9
4B	140	125 (89.3)	2–10	81 (57.9)	1–5	29	1.52	0–13.61	126	4.34	2–8
5A	357	330 (92.4)	2–27	171 (47.9)	1–8	65	1.10	0–6.06	277	4.26	2–14
5B	472	441 (93.4)	2–35	215 (45.6)	1–7	77	1.20	0–10.24	335	4.35	2–8
6A	342	331 (96.8)	2–56	132 (38.6)	1–7	47	1.86	0–10.64	212	4.51	2–11
6B	351	314 (89.5)	2–52	185 (52.7)	1–9	70	0.77	0–6.24	279	3.99	2–13
7A	343	319 (93.0)	2–16	182 (53.1)	1–5	81	0.72	0–7.39	290	3.58	2–7
7B	236	214 (90.7)	2–11	134 (56.8)	1–5	52	1.06	0–8.09	216	4.15	2–9
Total/Mean	4816	4448 (92.4)		2347 (48.7)		878	1.16		3671	4.18	
Range	140–529	125–491	2–56	81–261	0–12	29–96	0.69–1.86	0–13.61	126–410	3.58–4.51	1–25

HapB: haplotype block; Hap: haplotype.
