# Supplementary material for: The iSelect 9 K SNP analysis revealed polyploidization induced revolutionary changes and intense human selection causing strong haplotype blocks in wheat
Source: Sci Rep. 2017 Jan 30;7:41247. doi: 10.1038/srep41247 (PMC5278348; doi:10.1038/srep41247)
Supplement: Supplementary Information [file srep41247-s1.pdf]

The iSelect 9K SNP analysis revealed polyploidization induced revolutionary changes and intense human selection causing strong haplotype blocks in wheat

Chenyang Hao[1], Yuquan Wang[1], Shiaoman Chao[2], Tian Li[1], Hongxia Liu[1], Lanfen Wang[1] & Xueyong Zhang[1]*

[1]Key Laboratory of Crop Gene Resources and Germplasm Enhancment, Ministry of Agriculture/The National Key Facility for Crop Gene Resources and Genetic Improvement/Institute of Crop Science, Chinese Academy of Agricultural Sciences, Beijing 100081, China.

[2]US Department of Agriculture–Agricultural Research Service Biosciences Research Laboratory, Fargo, ND 58102, USA.

*Correspondence and requests for materials should be addressed to X.Z. (email: zhangxueyong@caas.cn).

Supplementary Information

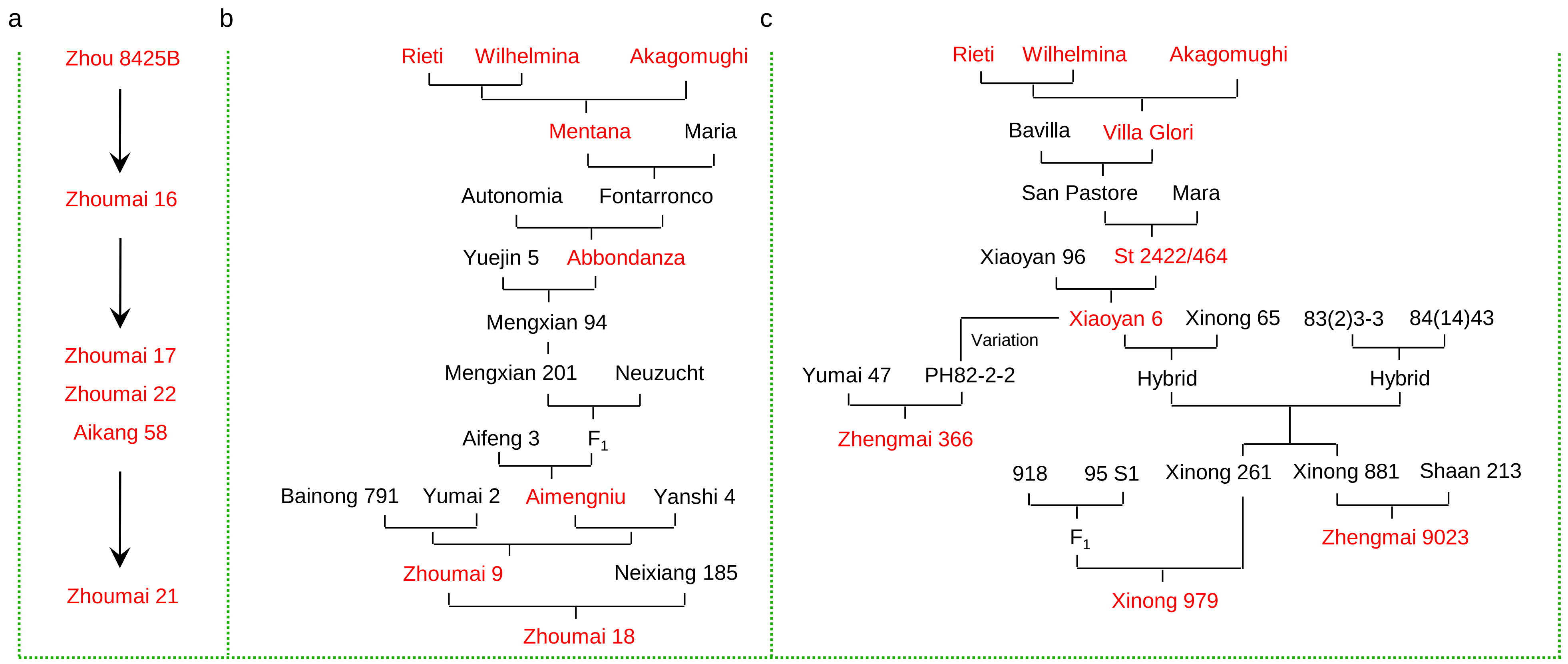

Supplementary Fig. S1 Basic pedigree relationship of related cultivars mainly derived from Zhou 8425B (a) and Akagomughi (b and c). Cultivars used in this study were marked by red color.

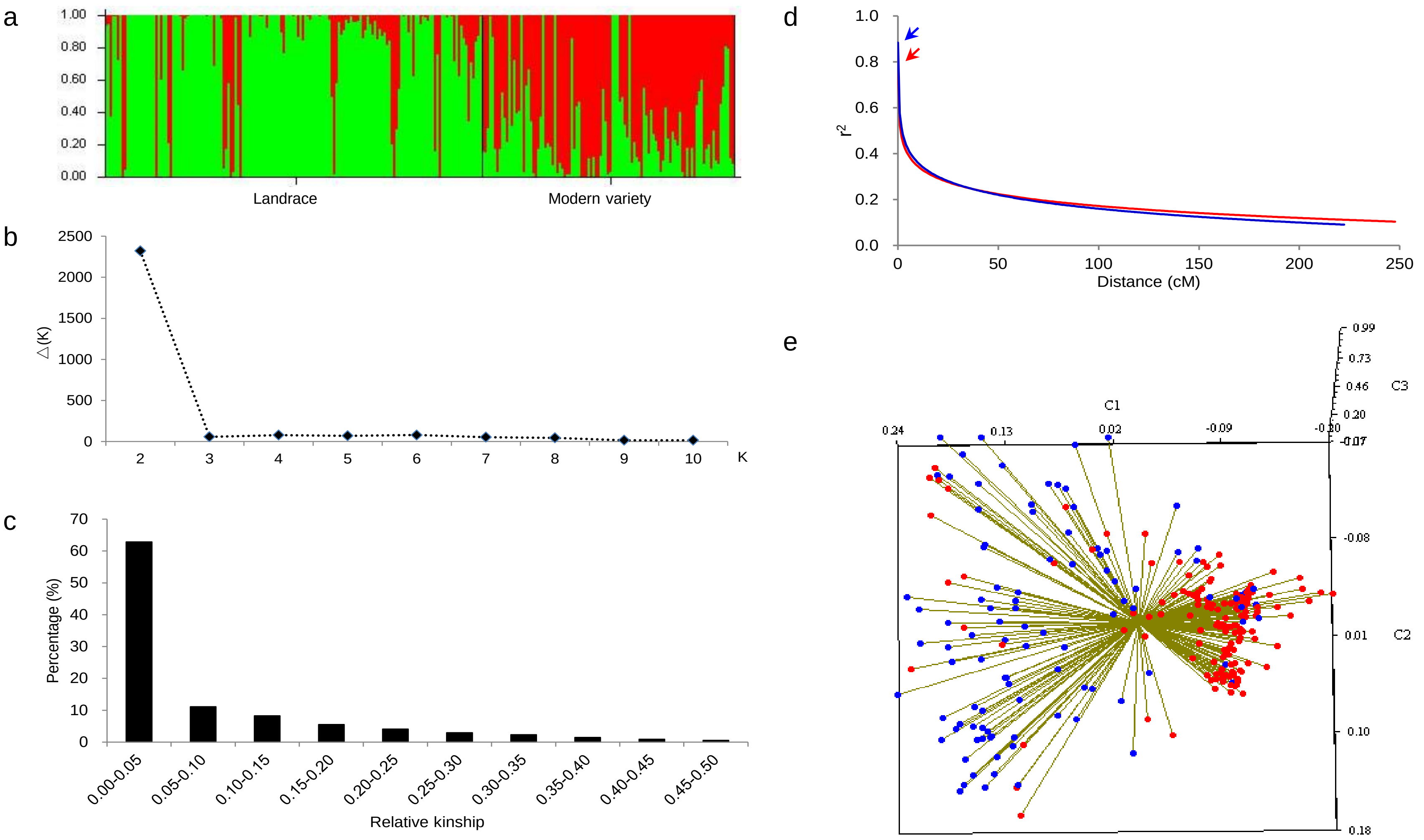

Supplementary Fig. S2 Population structure and linkage disequilibrium analysis of Chinese wheat mini core collections using 5756 polymorphic SNP markers. (a) Bayesian clustering (STRUCTURE, K = 2) of the MCC. (b) Estimation of the number of populations by calculating delta K values. (c) Distribution of pair-wise kinship coefficients among accessions. (d) Average LD decay in landraces (red) and modern cultivars (blue) accessions. (e) Principal coordinate analysis of landraces (red) and modern cultivars (blue) accessions.

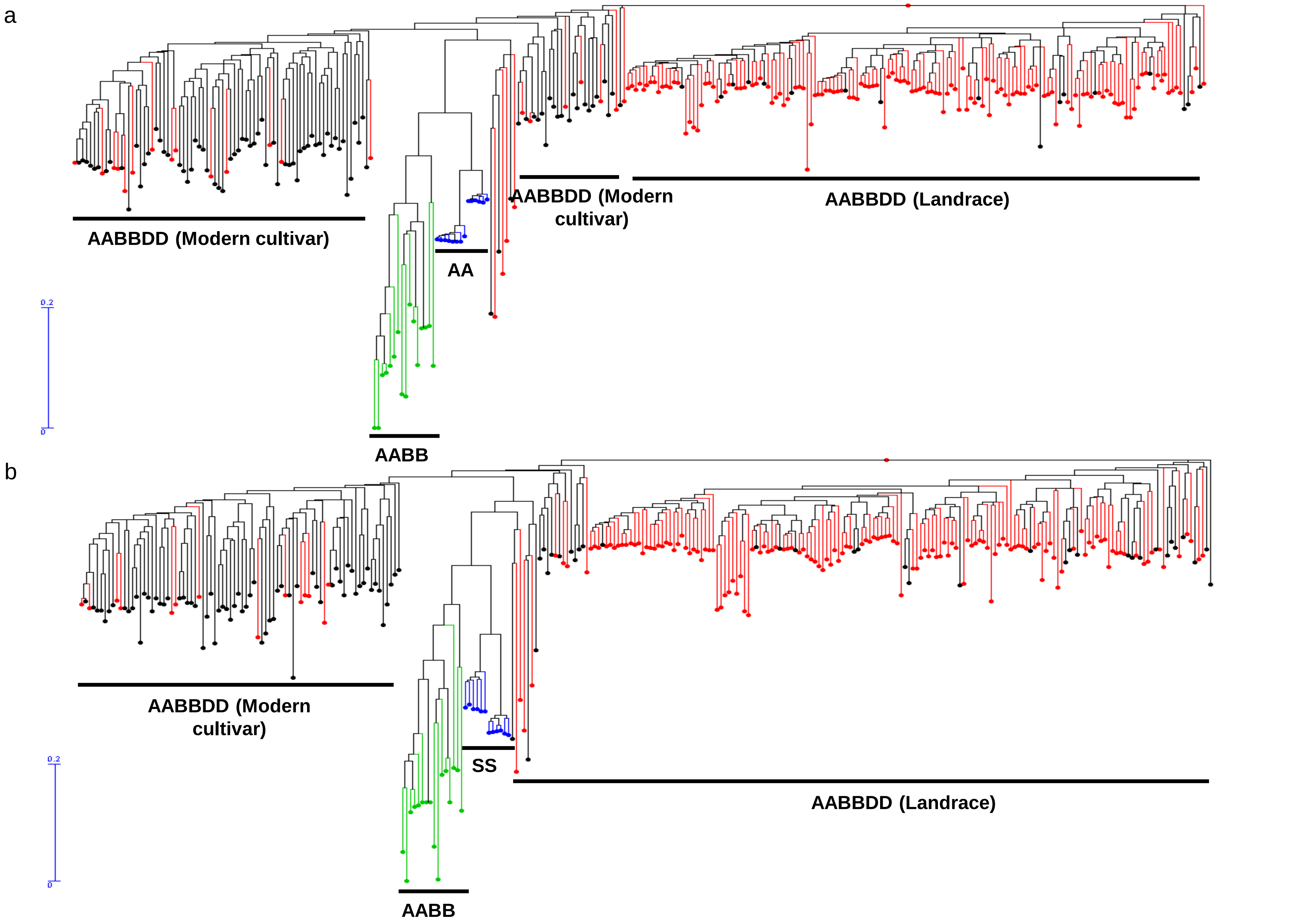

Supplementary Fig. S3 Dendrogram tree of diploid, tetraploid and hexaploid wheats in the A (a) and B (b) genome. The blue, green, red and black mean diploids, tetraploids, landraces and modern cultivar, respectively.

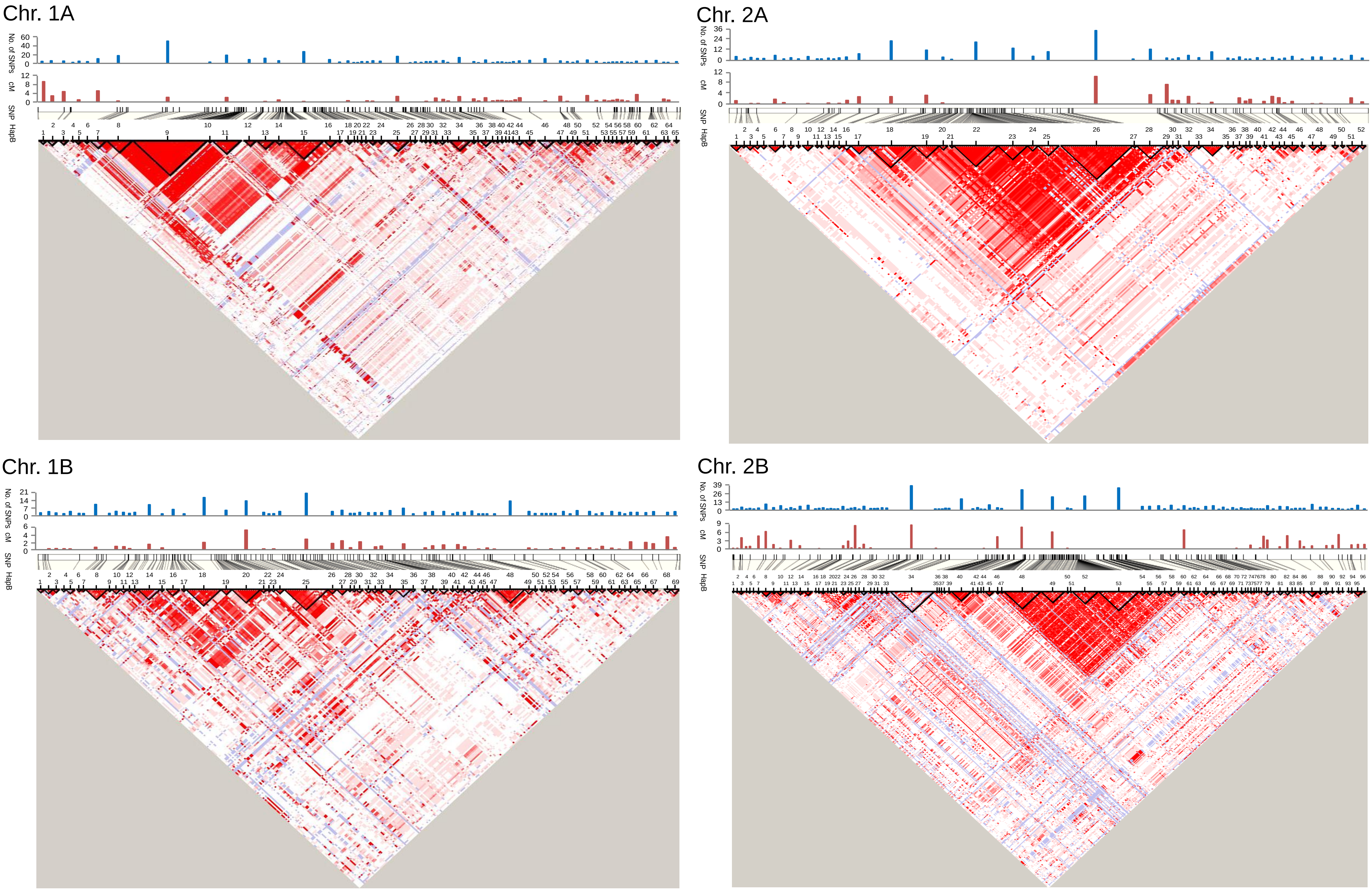

Supplementary Fig. S4 Haplotype block maps and their block length and SNP number in Chinese wheat mini core collections using SNP markers on the A and the B genome chromosomes.

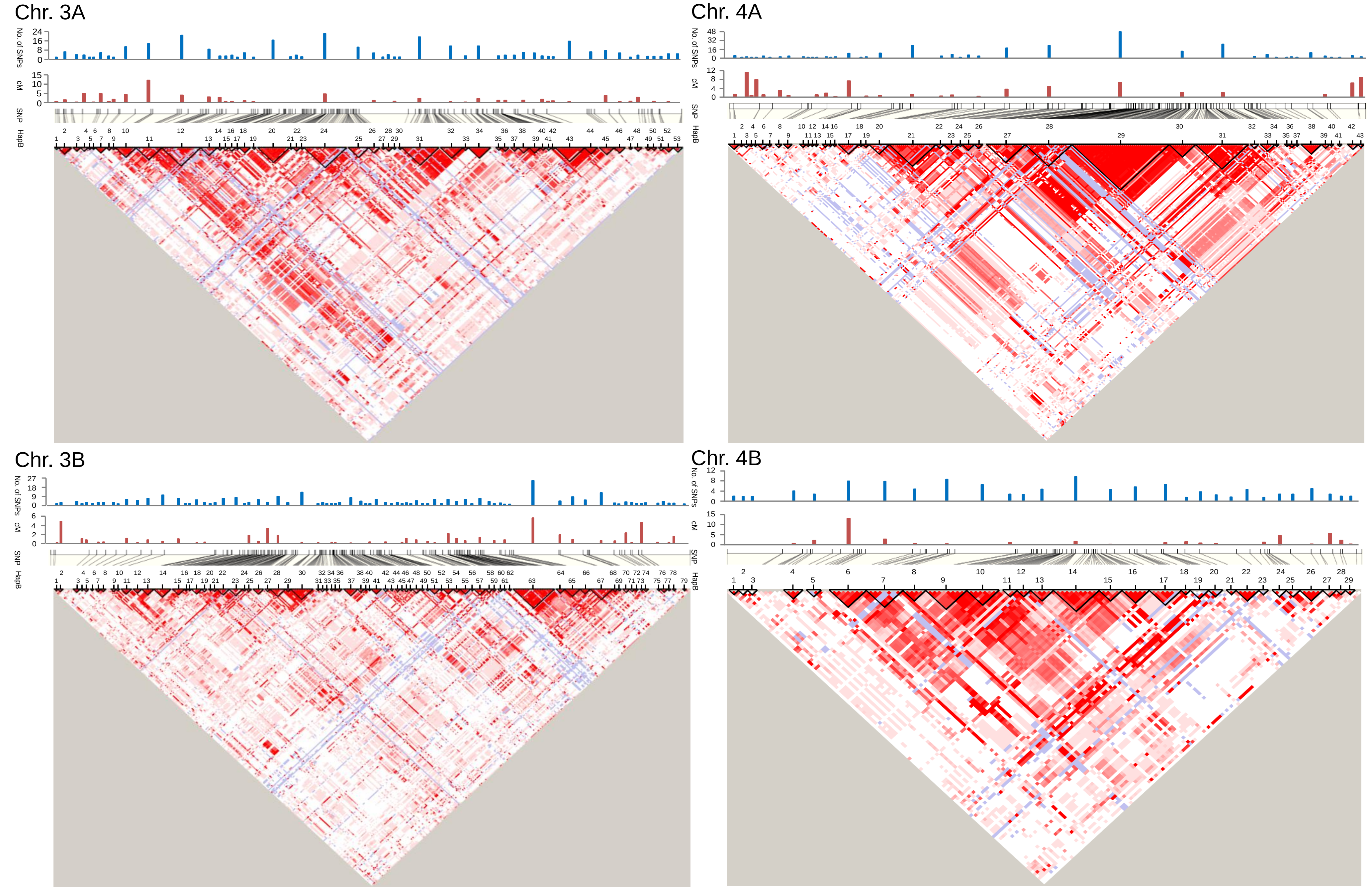

Supplementary Fig. S4_continued Haplotype block maps and their block length and SNP number in Chinese wheat mini core collections using SNP markers on the A and the B genome chromosomes.

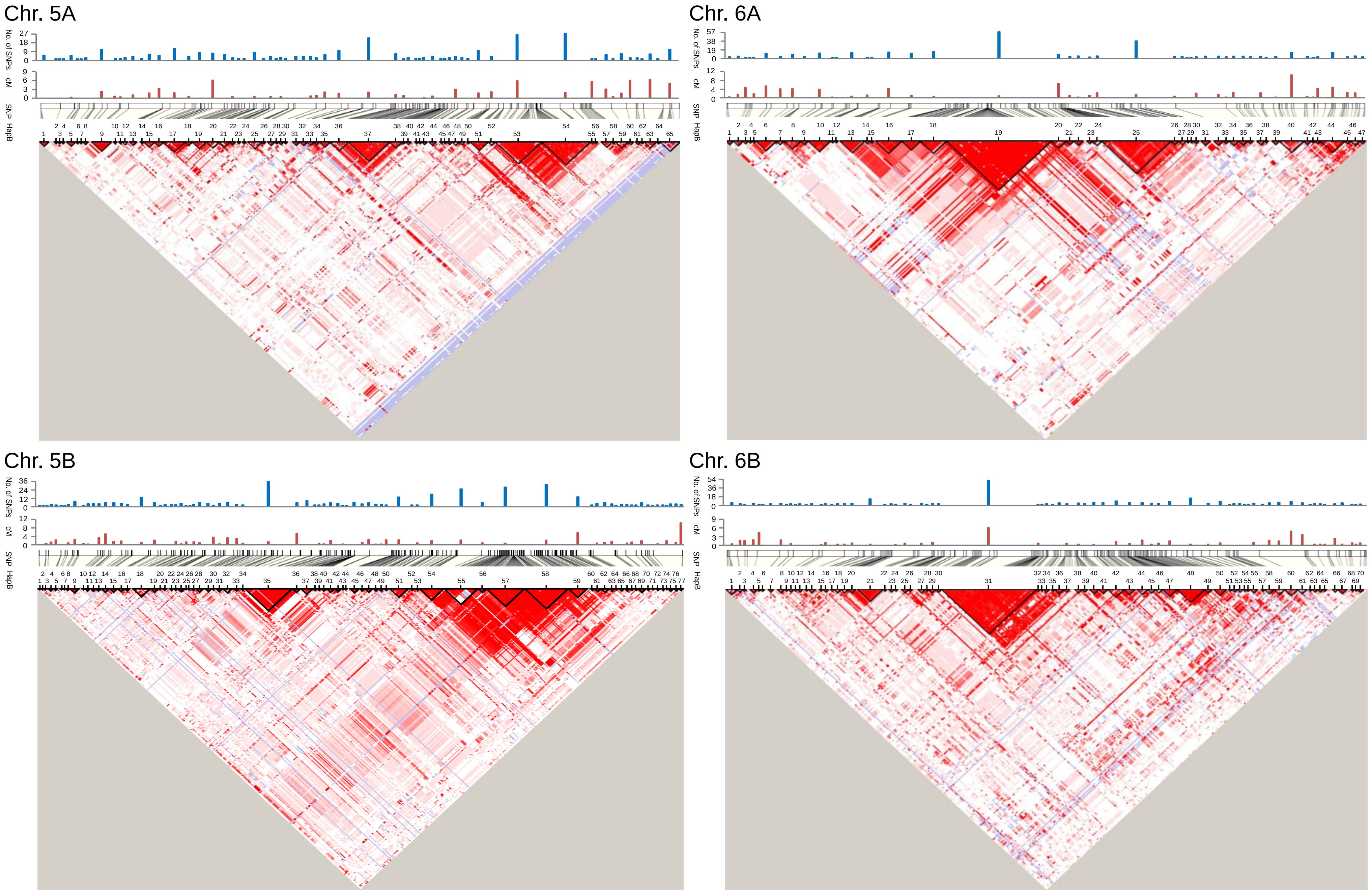

Supplementary Fig. S4_continued Haplotype block maps and their block length and SNP number in Chinese wheat mini core collections using SNP markers on the A and the B genome chromosomes.

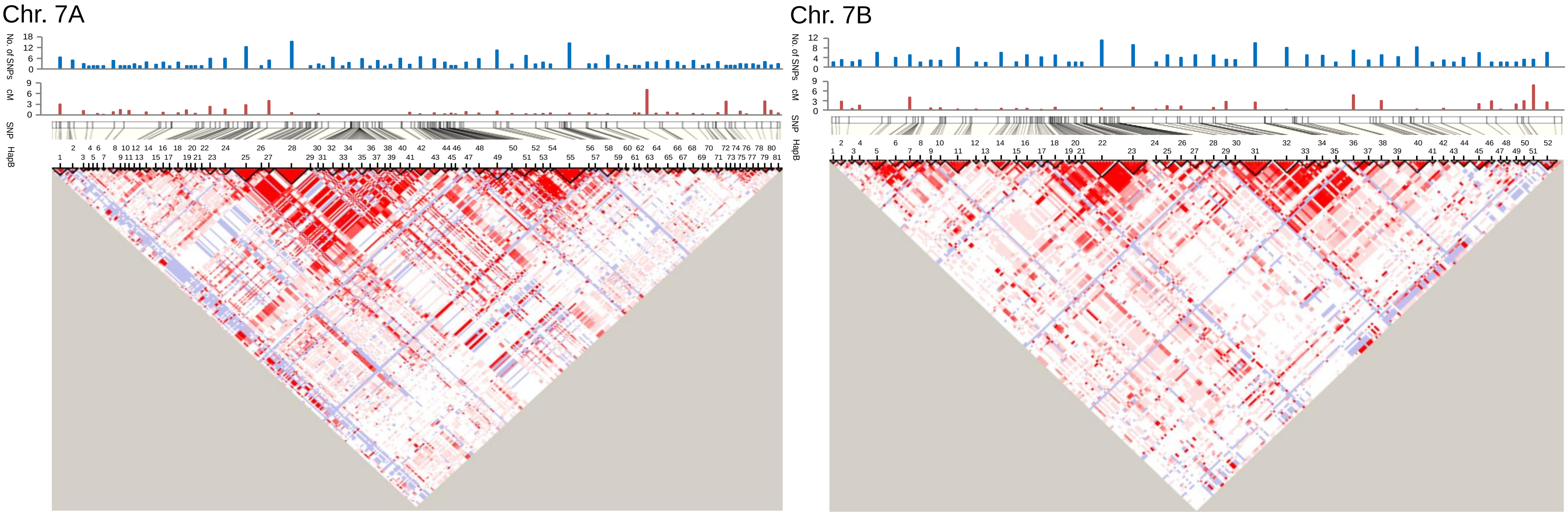

Supplementary Fig. S4_continued Haplotype block maps and their block length and SNP number in Chinese wheat mini core collections using SNP markers on the A and the B genome chromosomes.

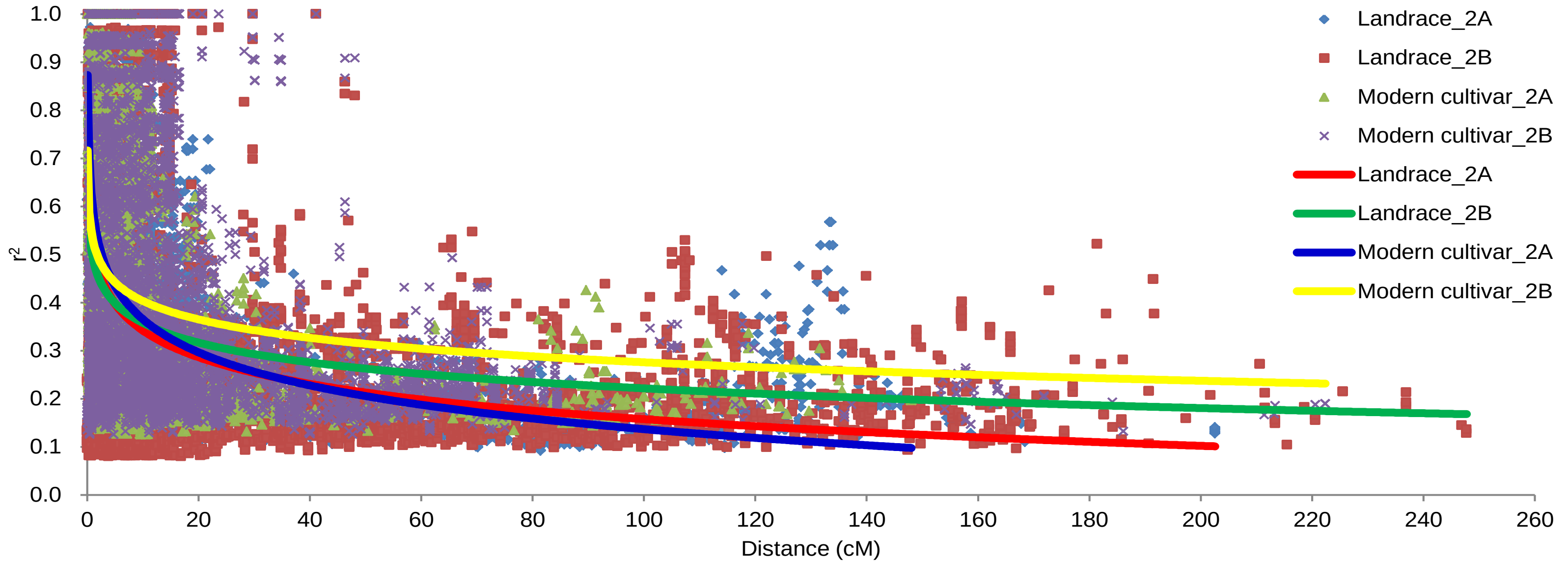

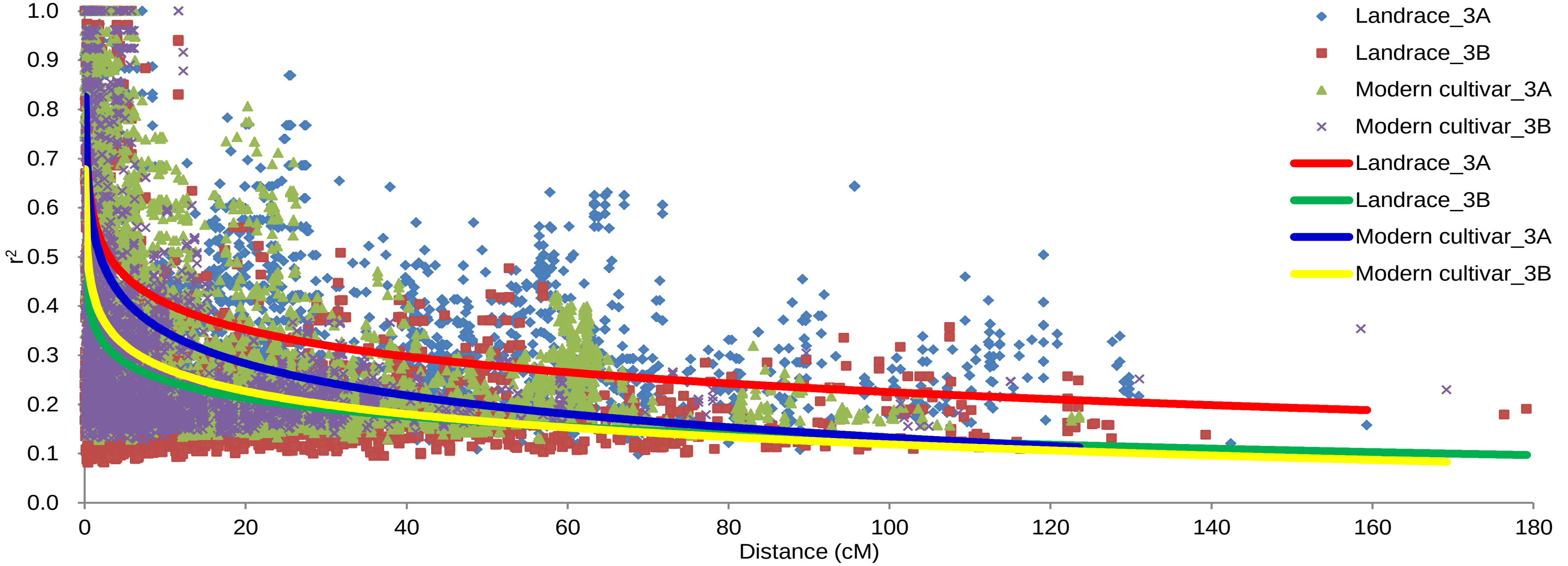

Supplementary Fig. S5 LD analysis for 2A vs 2B, 3A vs 3B, 4A vs 4B, 5A vs 5B, 7A vs 7B in landraces and modern cultivars respectively in MCC.

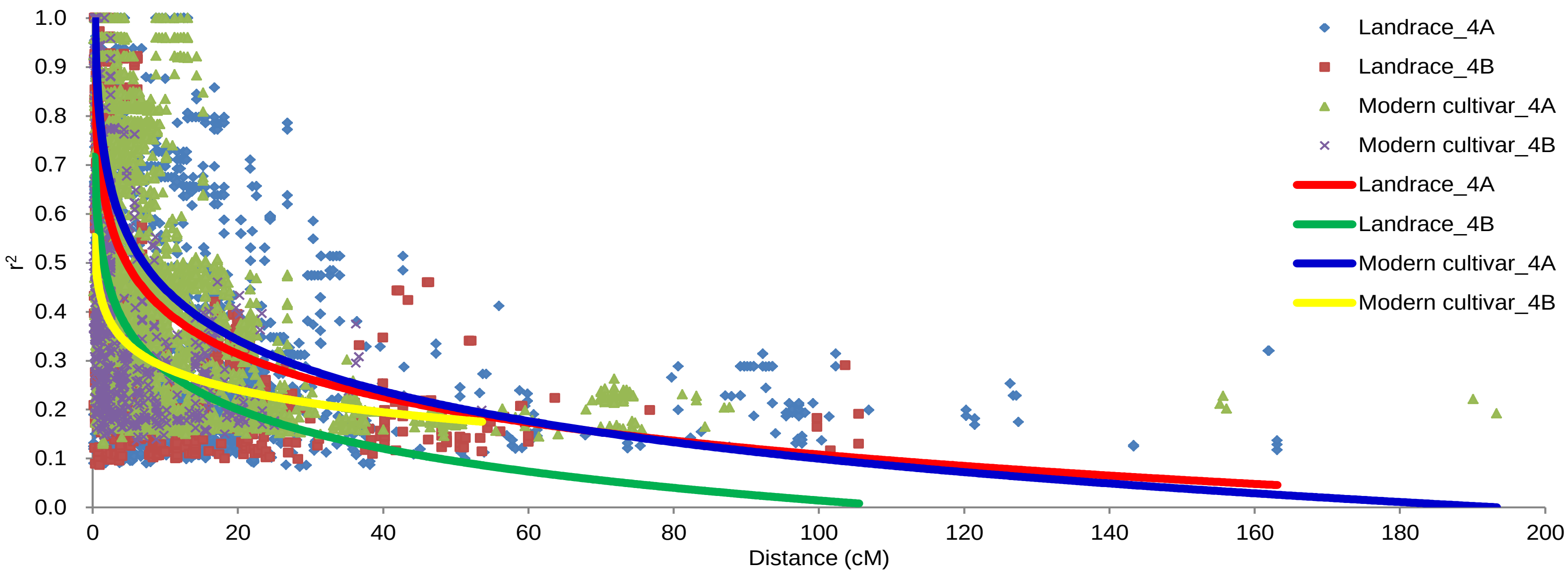

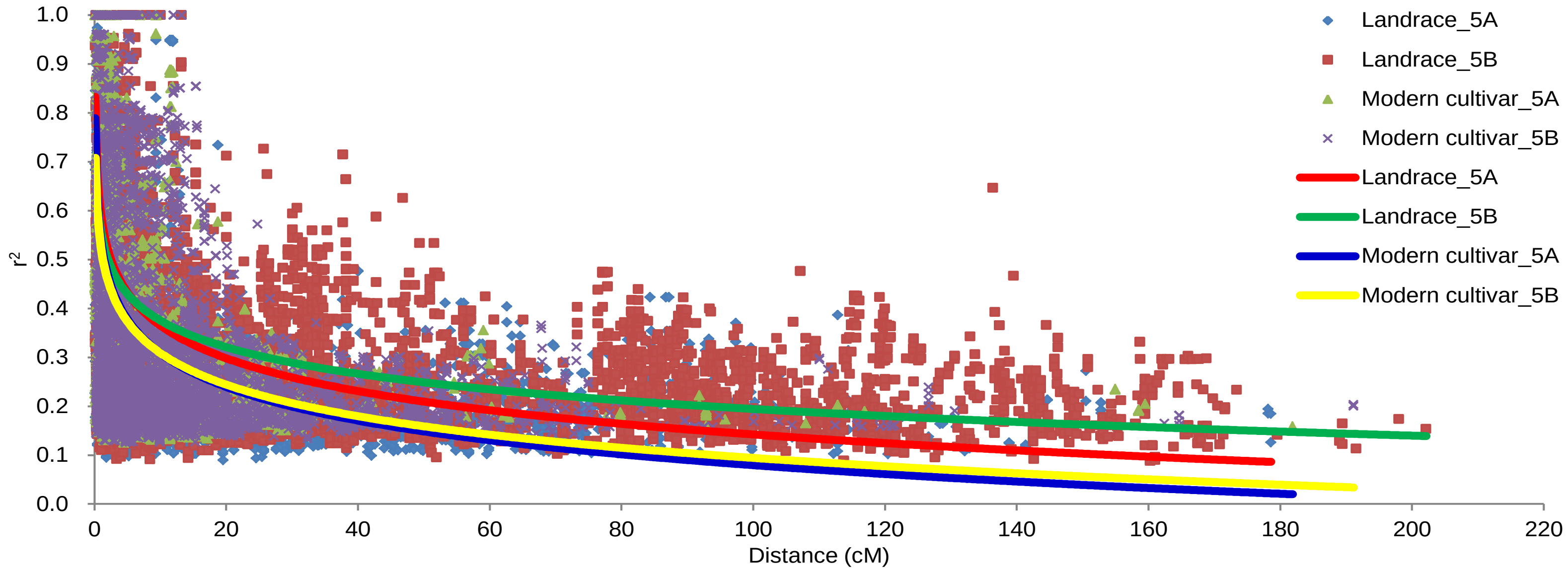

Supplementary Fig. S5_contunued LD analysis for 2A vs 2B, 3A vs 3B, 4A vs 4B, 5A vs 5B, 7A vs 7B in landraces and modern cultivars respectively in MCC.

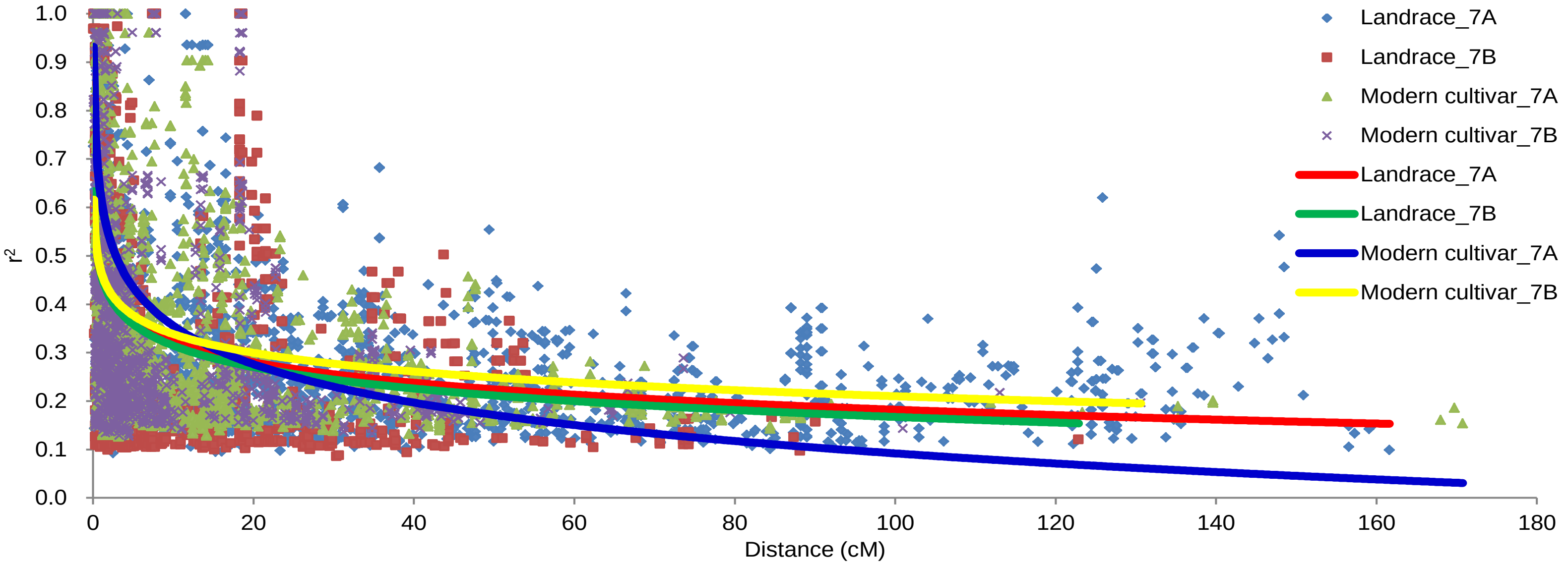

Supplementary Fig. S5_contunued LD analysis for 2A vs 2B, 3A vs 3B, 4A vs 4B, 5A vs 5B, 7A vs 7B in landraces and modern cultivars respectively in MCC.

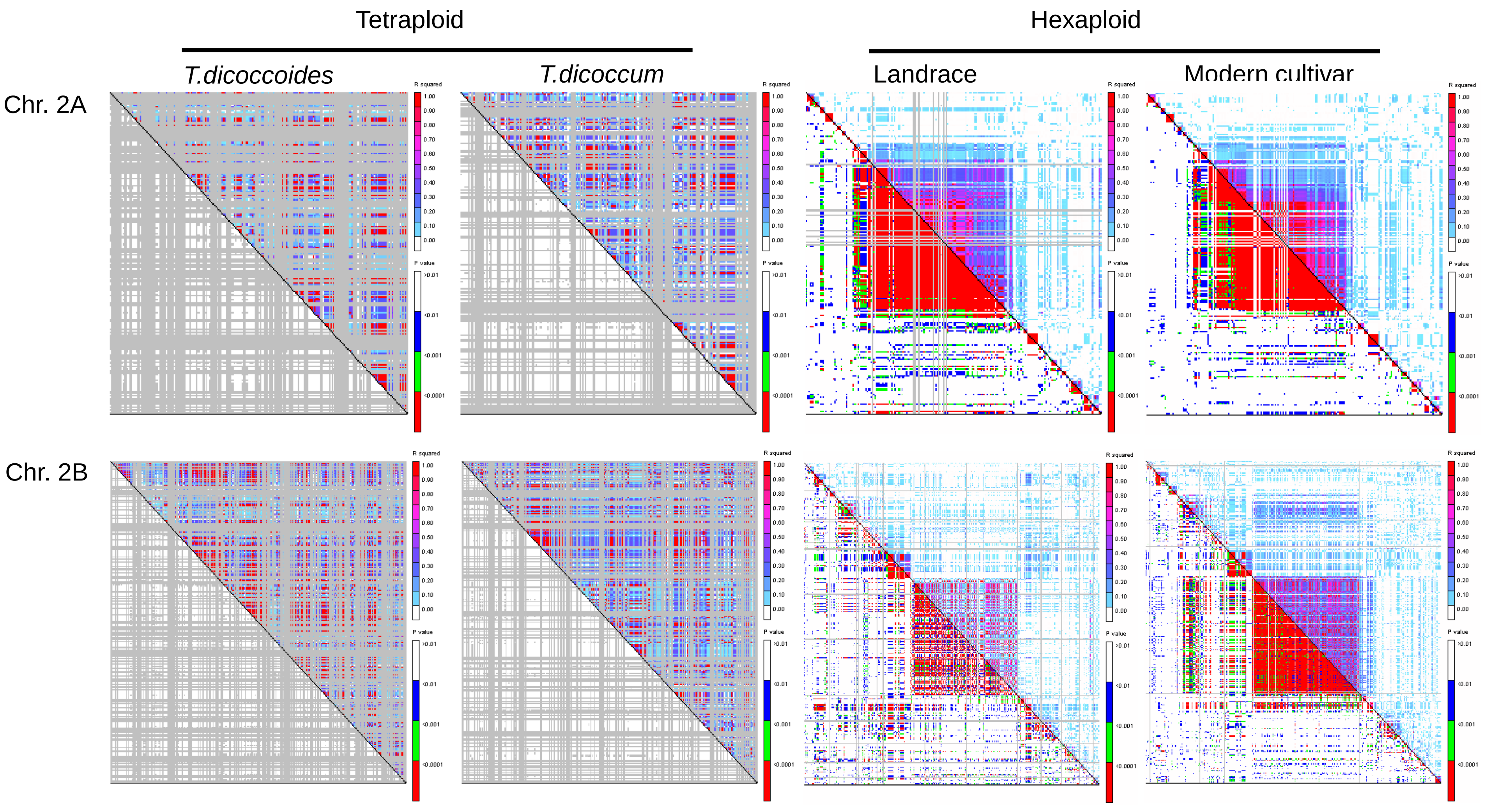

Supplementary Fig. S6 Comparison for haplotype blocks on 2A vs 2B, 3A vs 3B, 4A vs 4B, 5A vs 5B, 7A vs 7B in *Triticum dicoccoides*, *T. dicoccucum*, *T. aestivum* cv. landraces and modern cultivars.

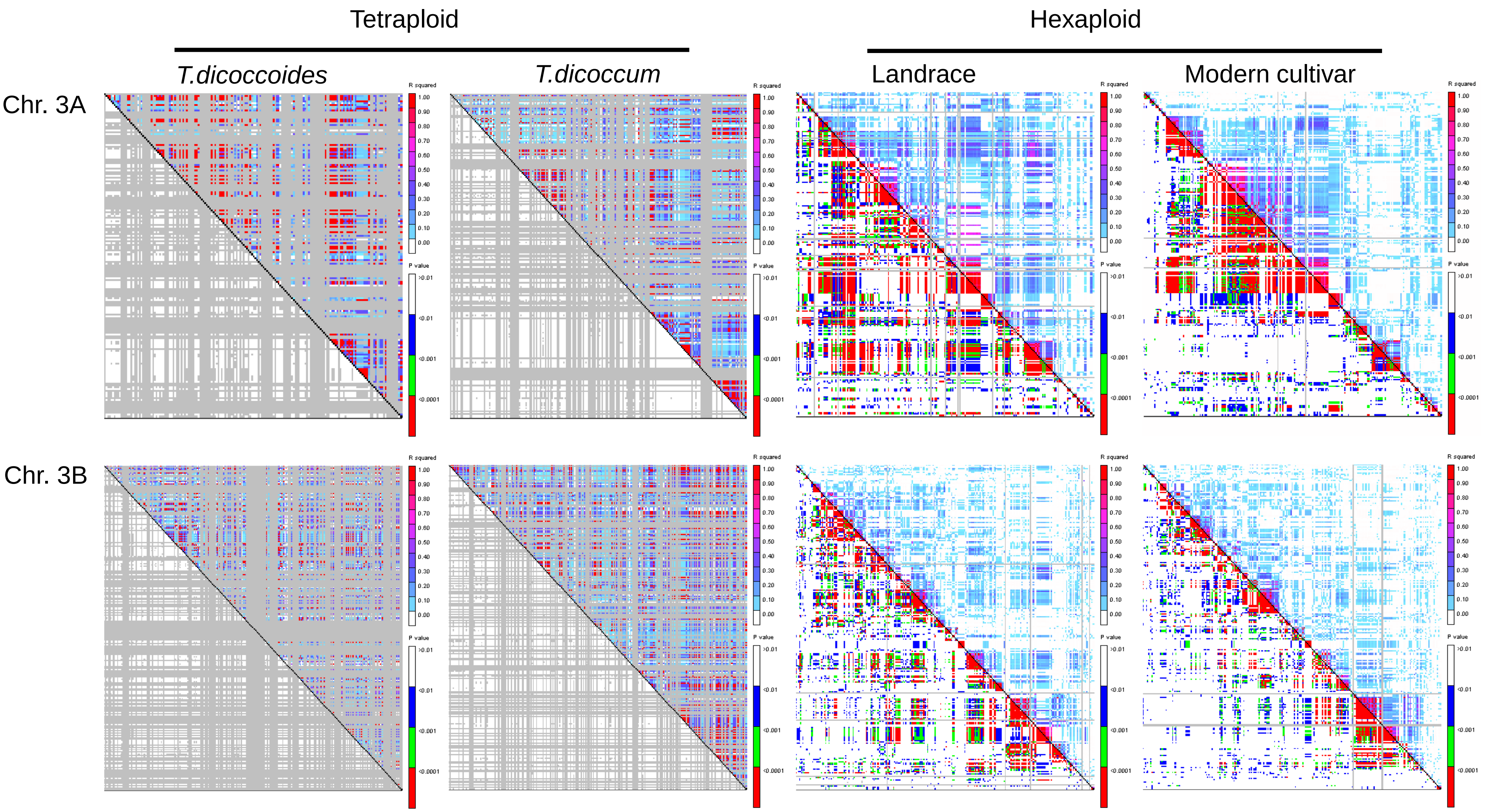

Supplementary Fig. S6_continued Comparison for haplotype blocks on 2A vs 2B, 3A vs 3B, 4A vs 4B, 5A vs 5B, 7A vs 7B in *Triticum dicoccoides*, *T. dicoccucum*, *T. aestivum* cv. landraces and modern cultivars.

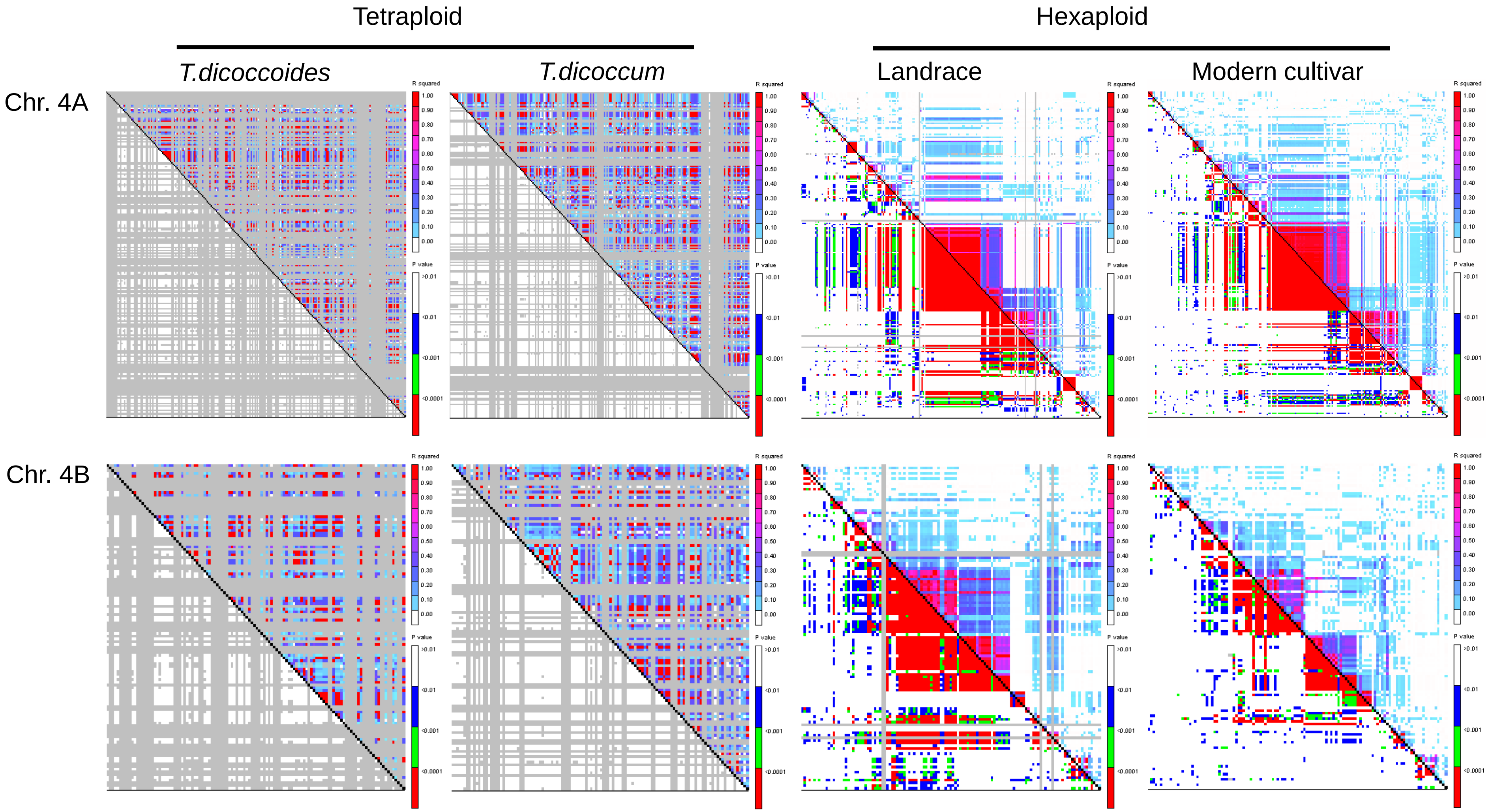

Supplementary Fig. S6_continued Comparison for haplotype blocks on 2A vs 2B, 3A vs 3B, 4A vs 4B, 5A vs 5B, 7A vs 7B in *Triticum dicoccoides*, *T. dicoccucum*, *T. aestivum* cv. landraces and modern cultivars.

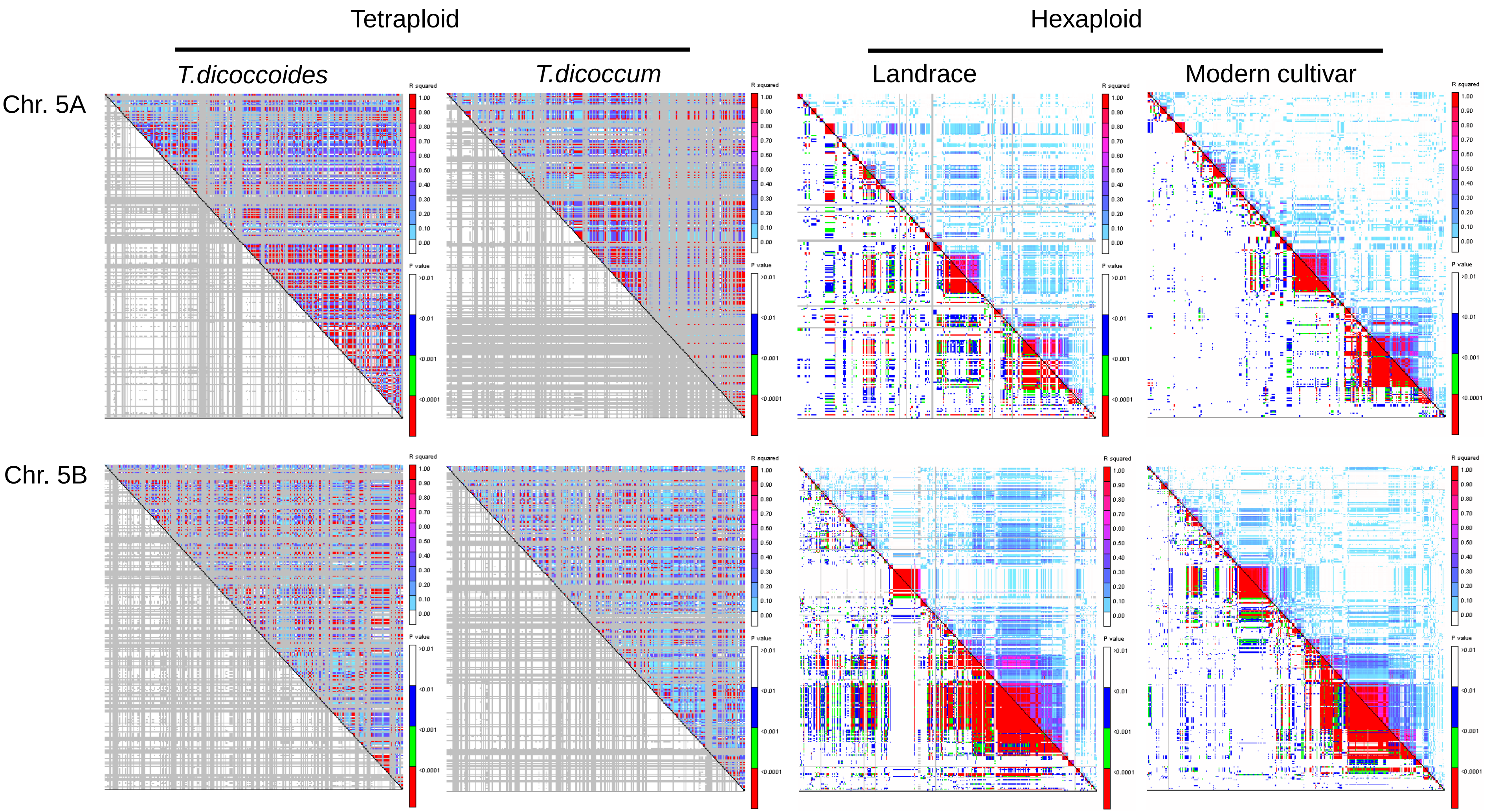

Supplementary Fig. S6_continued Comparison for haplotype blocks on 2A vs 2B, 3A vs 3B, 4A vs 4B, 5A vs 5B, 7A vs 7B in *Triticum dicoccoides*, *T. dicoccucum*, *T. aestivum* cv. landraces and modern cultivars.

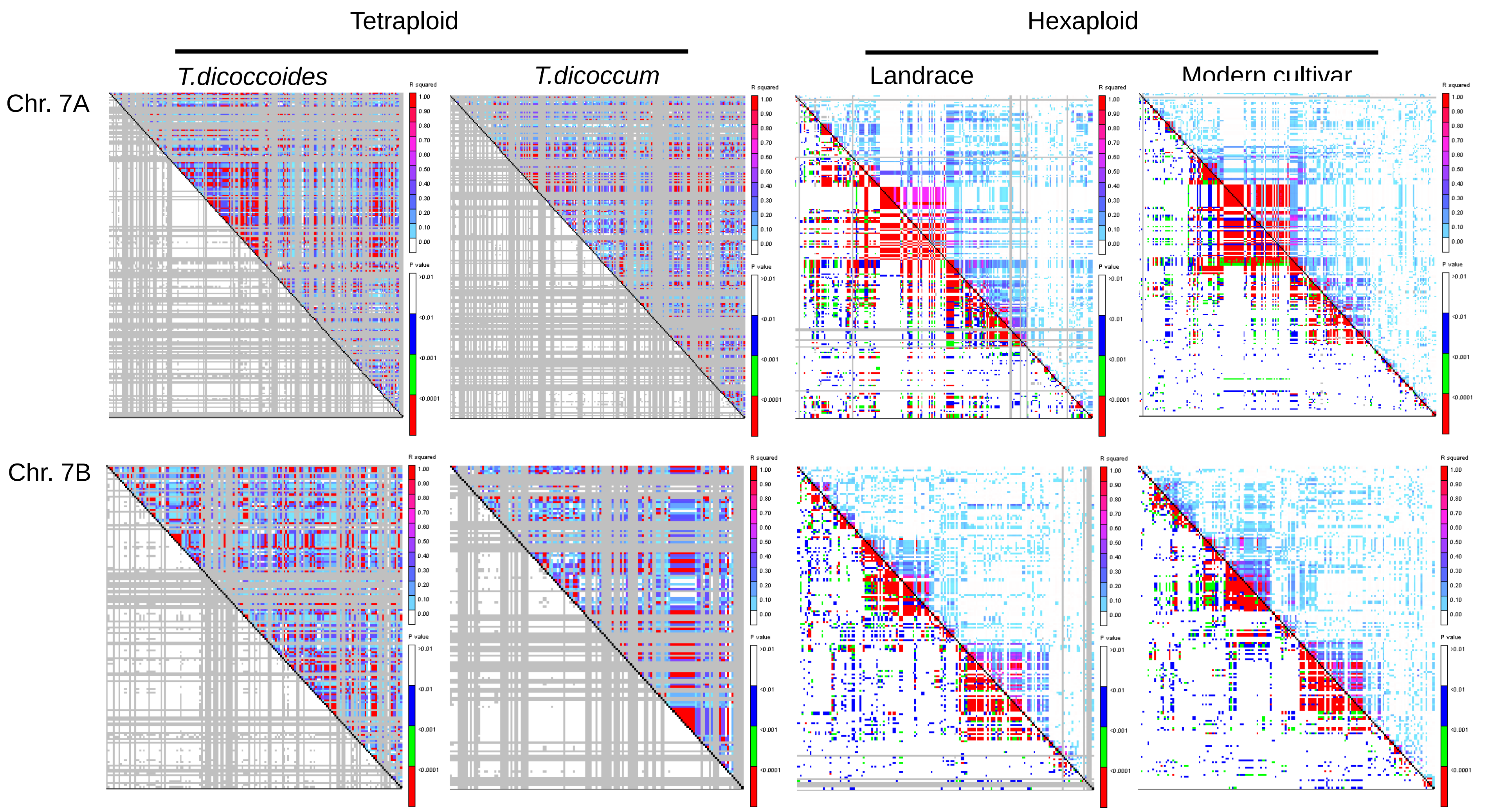

Supplementary Fig. S6_continued Comparison for haplotype blocks on 2A vs 2B, 3A vs 3B, 4A vs 4B, 5A vs 5B, 7A vs 7B in *Triticum dicoccoides*, *T. dicoccucum*, *T. aestivum* cv. landraces and modern cultivars.

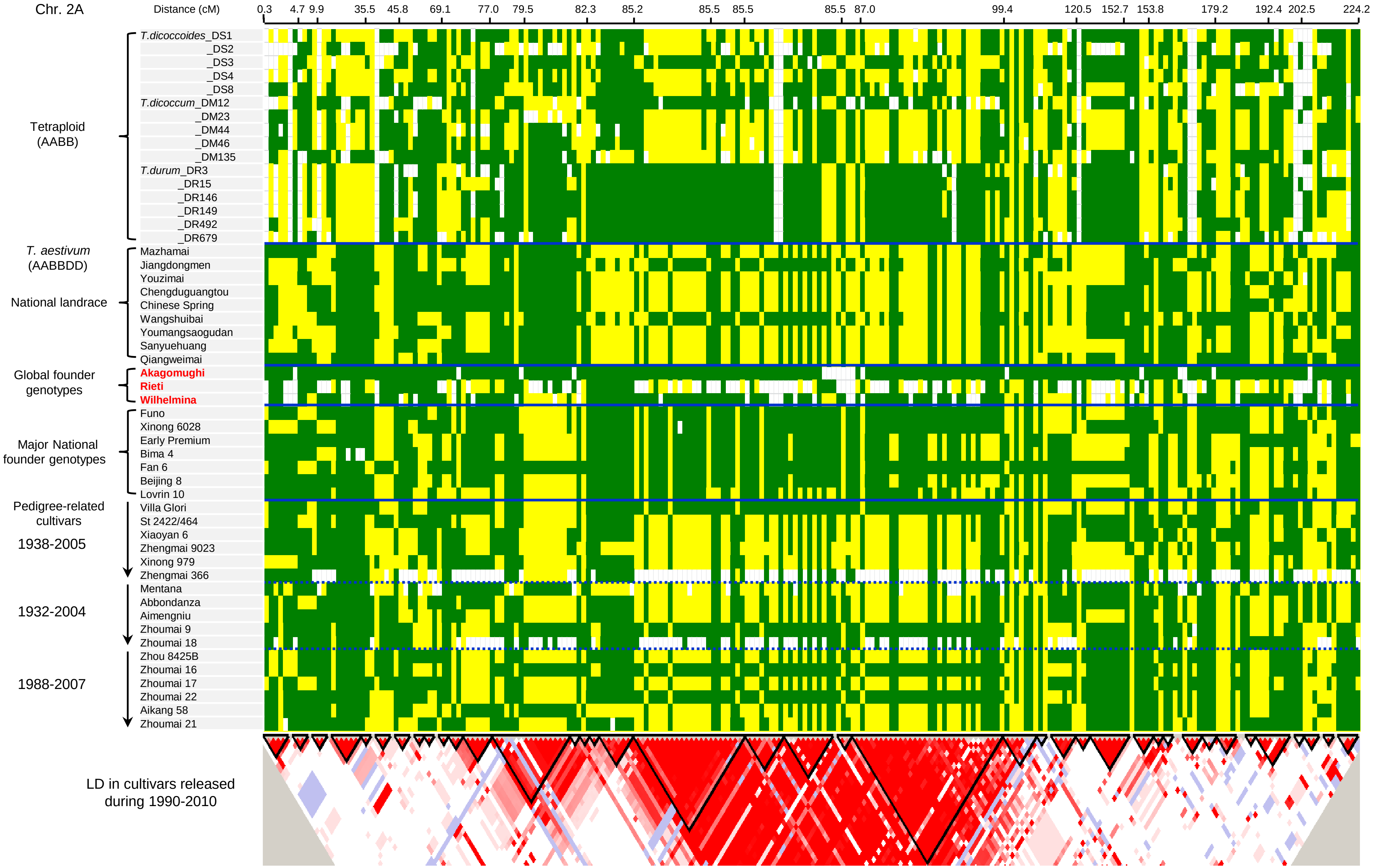

Supplementary Fig. S7 Formation and evolution of haplotype block on chromosomes 2A, 2B, 3A, 3B, 4A, 4B, 5A, 5B, 6A, 6B, 7A and 7B in one century of breeding. All SNP alleles in Akagomughi were assigned as green color. For other cultivars or collections, different alleles from Akagomughi were assigned as yellow, the missing SNP allele by white color. The figure at the bottom was haplotype block map made by Haploview 4.2 software in the newly released cultivars (1990-2010) in China based on SNP markers.

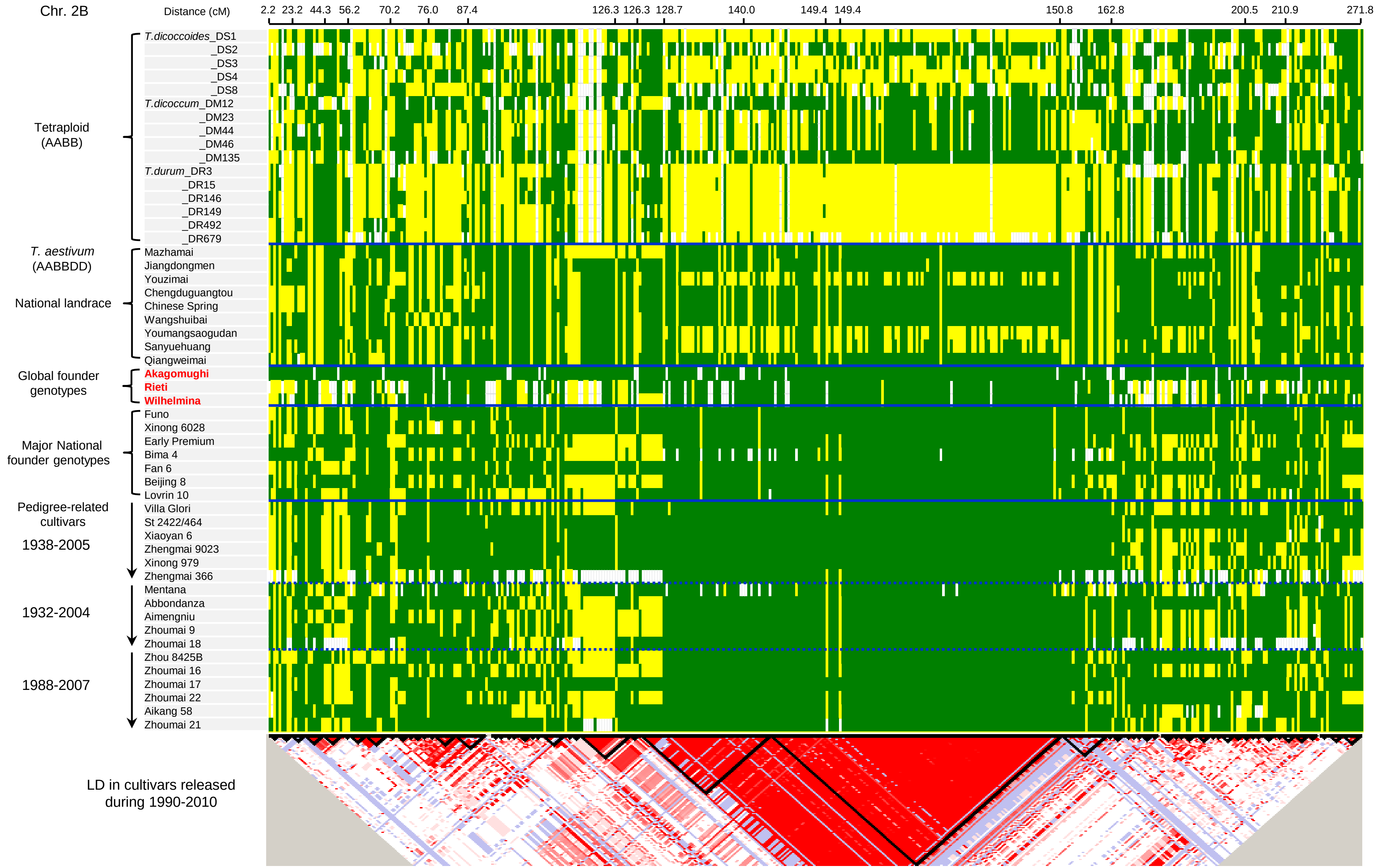

Supplementary Fig. S7_continued Formation and evolution of haplotype block on chromosomes 2A, 2B, 3A, 3B, 4A, 4B, 5A, 5B, 6A, 6B, 7A and 7B in one century of breeding. All SNP alleles in Akagomughi were assigned as green color. For other cultivars or collections, different alleles from Akagomughi were assigned as yellow, the missing SNP allele by white color. The figure at the bottom was haplotype block map made by Haploview 4.2 software in the newly released cultivars (1990-2010) in China based on SNP markers.

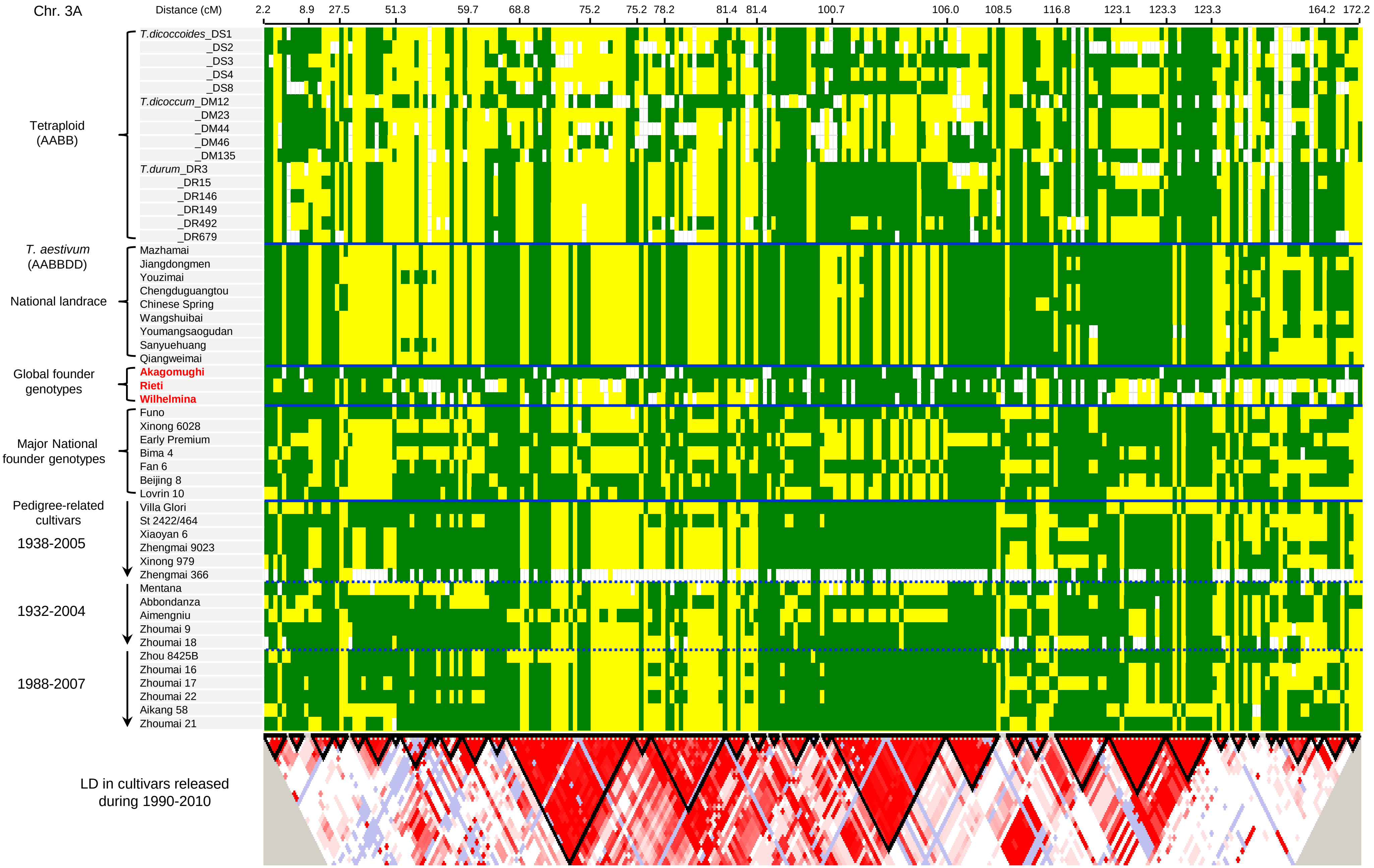

Supplementary Fig. S7_continued Formation and evolution of haplotype block on chromosomes 2A, 2B, 3A, 3B, 4A, 4B, 5A, 5B, 6A, 6B, 7A and 7B in one century of breeding. All SNP alleles in Akagomughi were assigned as green color. For other cultivars or collections, different alleles from Akagomughi were assigned as yellow, the missing SNP allele by white color. The figure at the bottom was haplotype block map made by Haploview 4.2 software in the newly released cultivars (1990-2010) in China based on SNP markers.

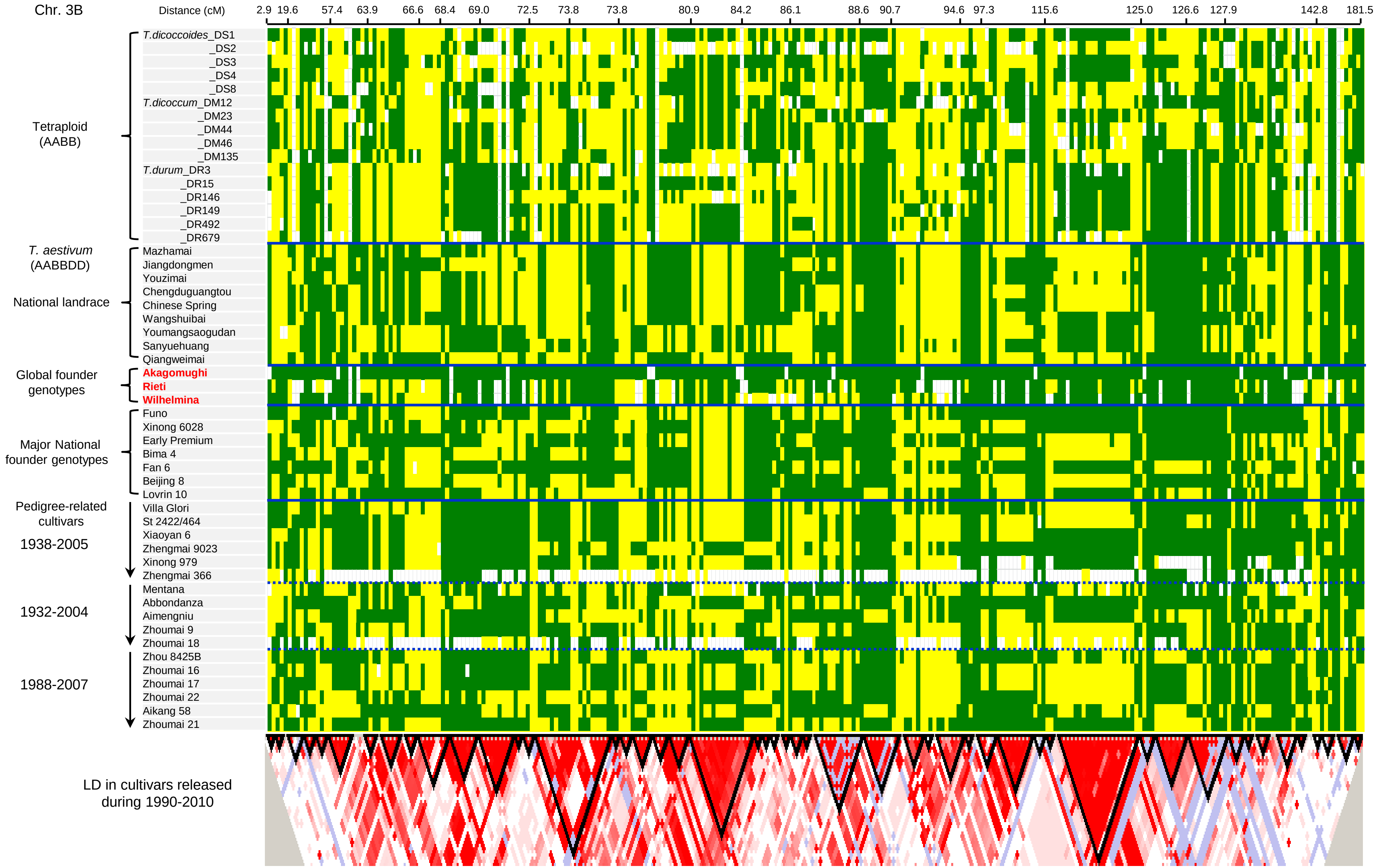

Supplementary Fig. S7_continued Formation and evolution of haplotype block on chromosomes 2A, 2B, 3A, 3B, 4A, 4B, 5A, 5B, 6A, 6B, 7A and 7B in one century of breeding. All SNP alleles in Akagomughi were assigned as green color. For other cultivars or collections, different alleles from Akagomughi were assigned as yellow, the missing SNP allele by white color. The figure at the bottom was haplotype block map made by Haploview 4.2 software in the newly released cultivars (1990-2010) in China based on SNP markers.

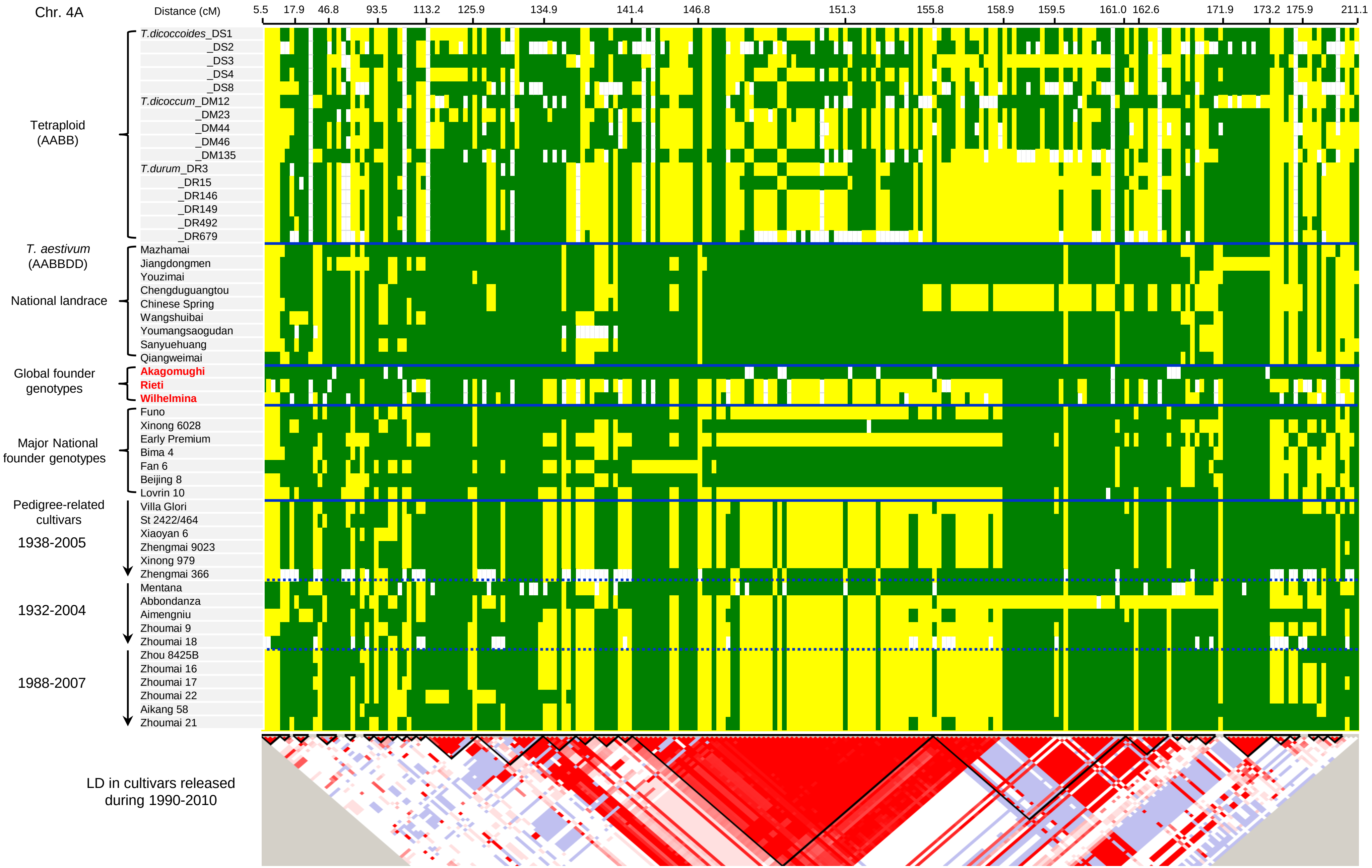

Supplementary Fig. S7_continued Formation and evolution of haplotype block on chromosomes 2A, 2B, 3A, 3B, 4A, 4B, 5A, 5B, 6A, 6B, 7A and 7B in one century of breeding. All SNP alleles in Akagomughi were assigned as green color. For other cultivars or collections, different alleles from Akagomughi were assigned as yellow, the missing SNP allele by white color. The figure at the bottom was haplotype block map made by Haploview 4.2 software in the newly released cultivars (1990-2010) in China based on SNP markers.

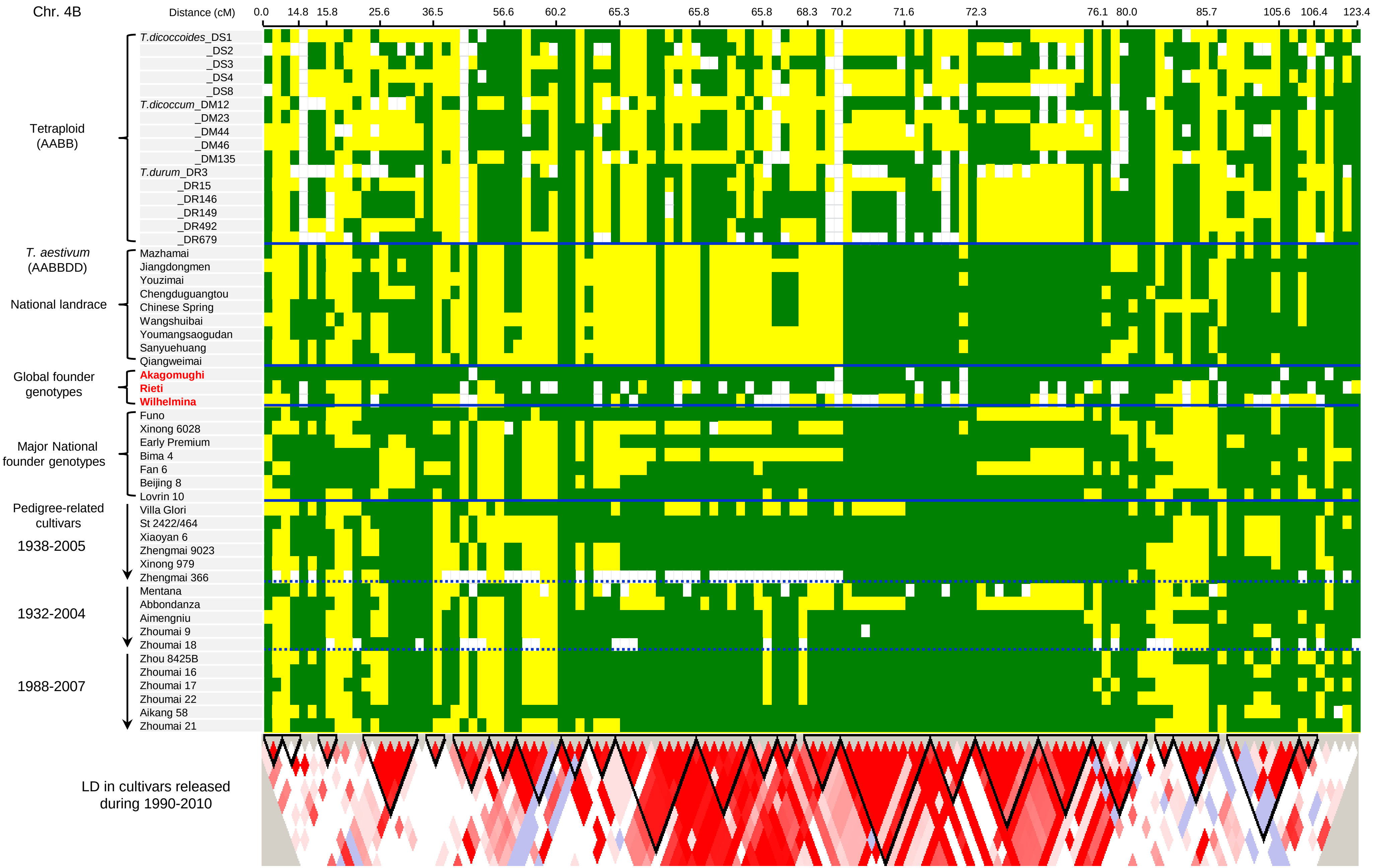

Supplementary Fig. S7_continued Formation and evolution of haplotype block on chromosomes 2A, 2B, 3A, 3B, 4A, 4B, 5A, 5B, 6A, 6B, 7A and 7B in one century of breeding. All SNP alleles in Akagomughi were assigned as green color. For other cultivars or collections, different alleles from Akagomughi were assigned as yellow, the missing SNP allele by white color. The figure at the bottom was haplotype block map made by Haploview 4.2 software in the newly released cultivars (1990-2010) in China based on SNP markers.

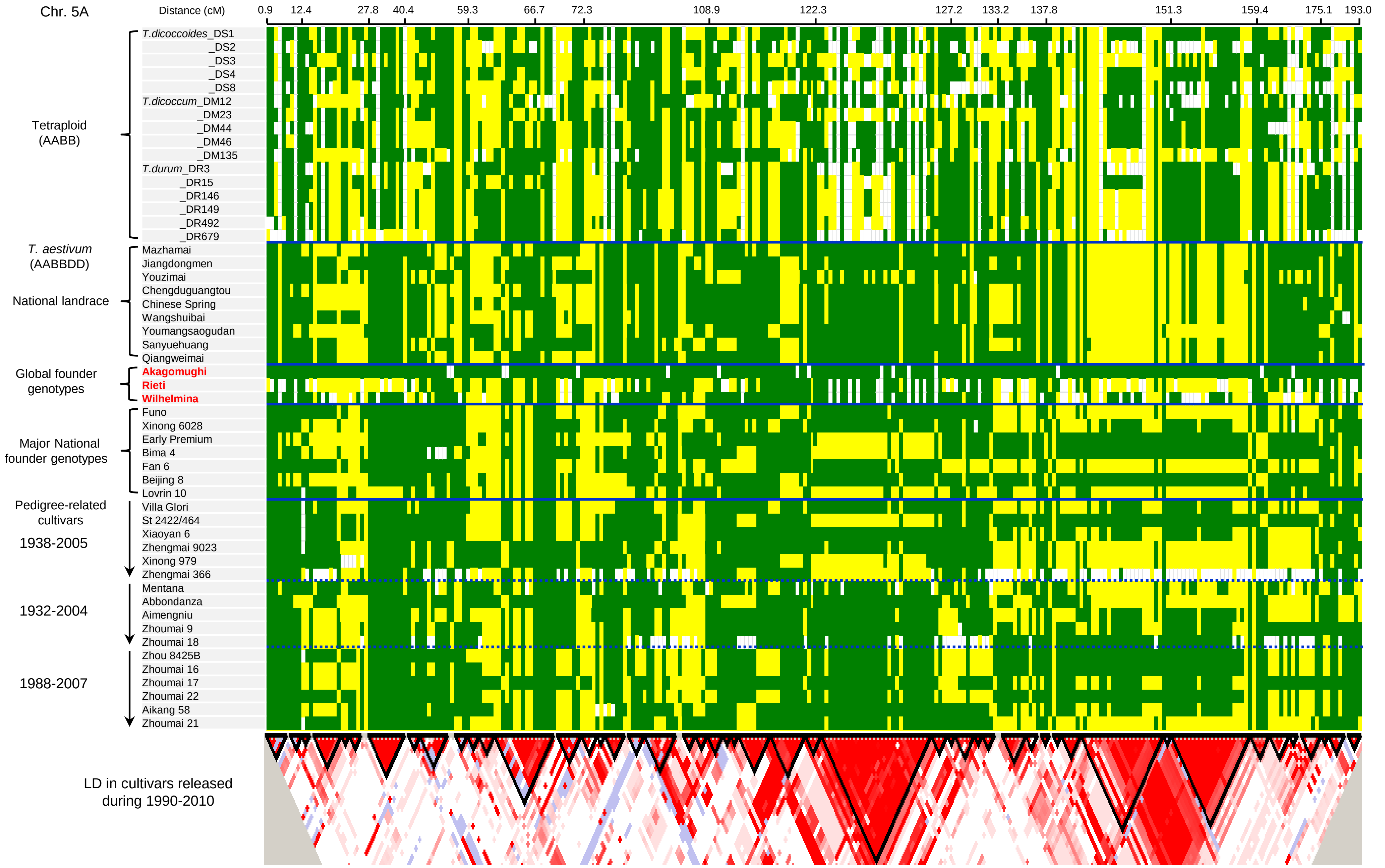

Supplementary Fig. S7_continued Formation and evolution of haplotype block on chromosomes 2A, 2B, 3A, 3B, 4A, 4B, 5A, 5B, 6A, 6B, 7A and 7B in one century of breeding. All SNP alleles in Akagomughi were assigned as green color. For other cultivars or collections, different alleles from Akagomughi were assigned as yellow, the missing SNP allele by white color. The figure at the bottom was haplotype block map made by Haploview 4.2 software in the newly released cultivars (1990-2010) in China based on SNP markers.

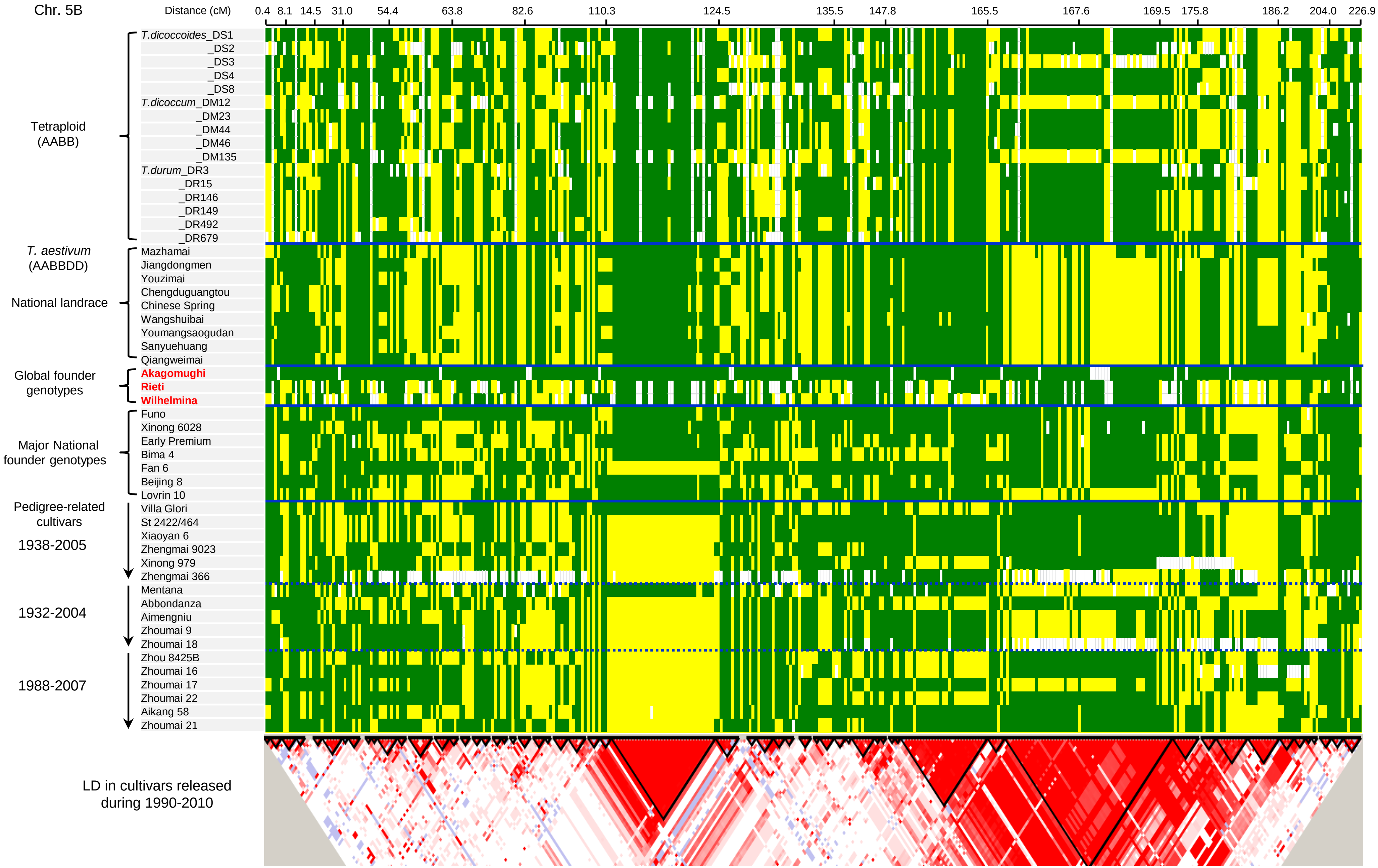

Supplementary Fig. S7_continued Formation and evolution of haplotype block on chromosomes 2A, 2B, 3A, 3B, 4A, 4B, 5A, 5B, 6A, 6B, 7A and 7B in one century of breeding. All SNP alleles in Akagomughi were assigned as green color. For other cultivars or collections, different alleles from Akagomughi were assigned as yellow, the missing SNP allele by white color. The figure at the bottom was haplotype block map made by Haploview 4.2 software in the newly released cultivars (1990-2010) in China based on SNP markers.

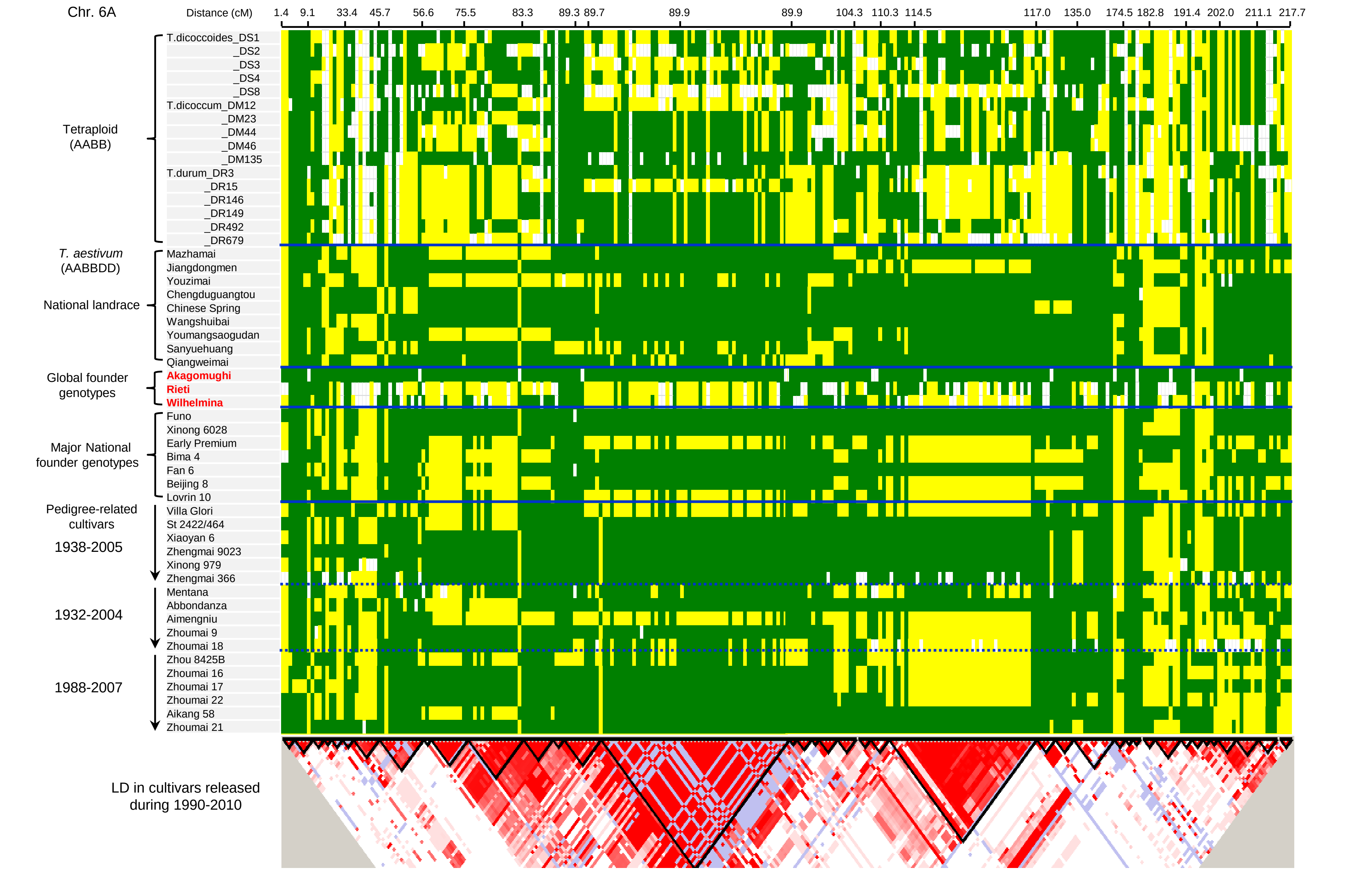

Supplementary Fig. S7_continued Formation and evolution of haplotype block on chromosomes 2A, 2B, 3A, 3B, 4A, 4B, 5A, 5B, 6A, 6B, 7A and 7B in one century of breeding. All SNP alleles in Akagomughi were assigned as green color. For other cultivars or collections, different alleles from Akagomughi were assigned as yellow, the missing SNP allele by white color. The figure at the bottom was haplotype block map made by Haploview 4.2 software in the newly released cultivars (1990-2010) in China based on SNP markers.

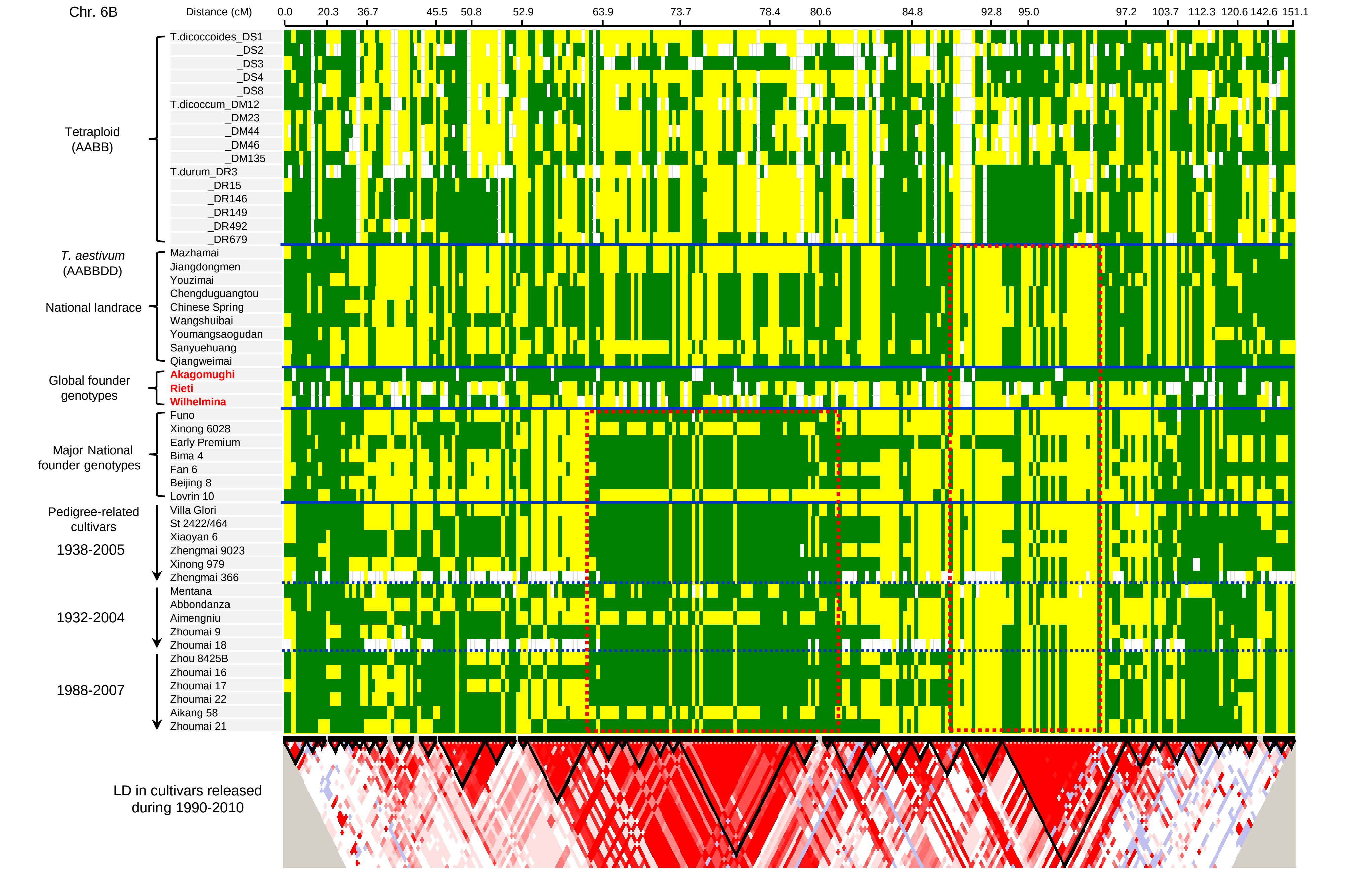

Supplementary Fig. S7_continued Formation and evolution of haplotype block on chromosomes 2A, 2B, 3A, 3B, 4A, 4B, 5A, 5B, 6A, 6B, 7A and 7B in one century of breeding. All SNP alleles in Akagomughi were assigned as green color. For other cultivars or collections, different alleles from Akagomughi were assigned as yellow, the missing SNP allele by white color. The figure at the bottom was haplotype block map made by Haploview 4.2 software in the newly released cultivars (1990-2010) in China based on SNP markers.

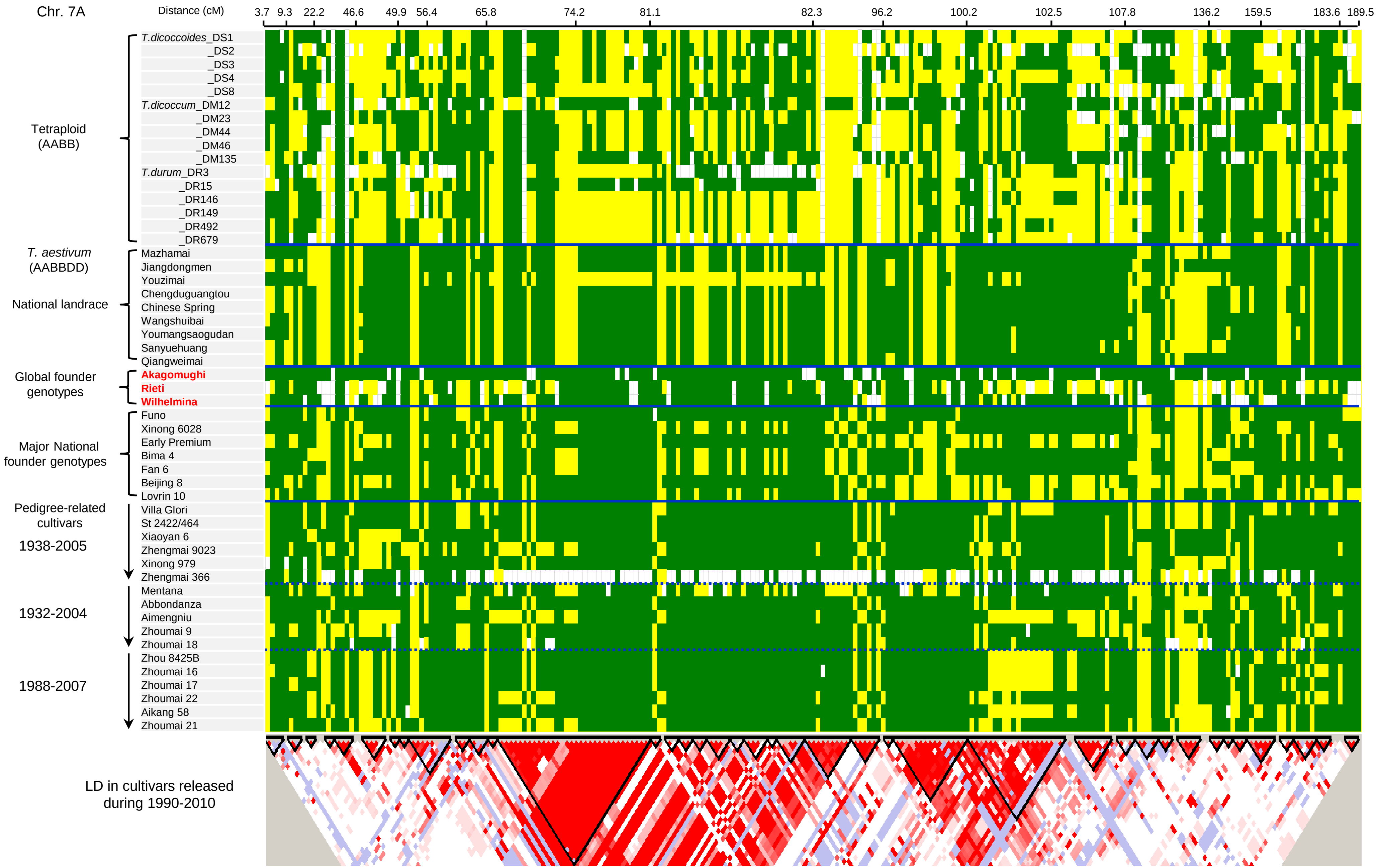

Supplementary Fig. S7_continued Formation and evolution of haplotype block on chromosomes 2A, 2B, 3A, 3B, 4A, 4B, 5A, 5B, 6A, 6B, 7A and 7B in one century of breeding. All SNP alleles in Akagomughi were assigned as green color. For other cultivars or collections, different alleles from Akagomughi were assigned as yellow, the missing SNP allele by white color. The figure at the bottom was haplotype block map made by Haploview 4.2 software in the newly released cultivars (1990-2010) in China based on SNP markers.

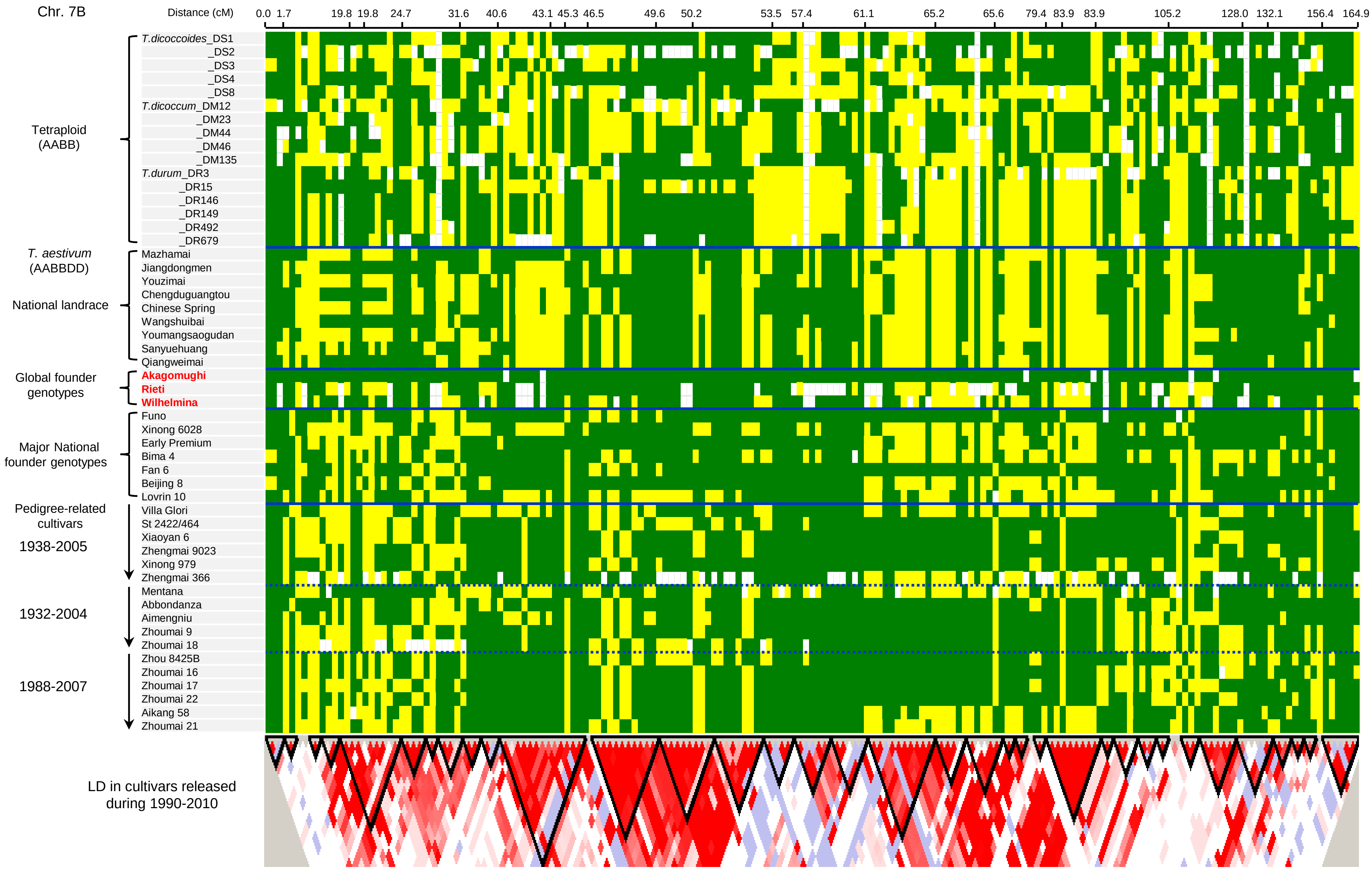

Supplementary Fig. S7_continued Formation and evolution of haplotype block on chromosomes 2A, 2B, 3A, 3B, 4A, 4B, 5A, 5B, 6A, 6B, 7A and 7B in one century of breeding. All SNP alleles in Akagomughi were assigned as green color. For other cultivars or collections, different alleles from Akagomughi were assigned as yellow, the missing SNP allele by white color. The figure at the bottom was haplotype block map made by Haploview 4.2 software in the newly released cultivars (1990-2010) in China based on SNP markers.

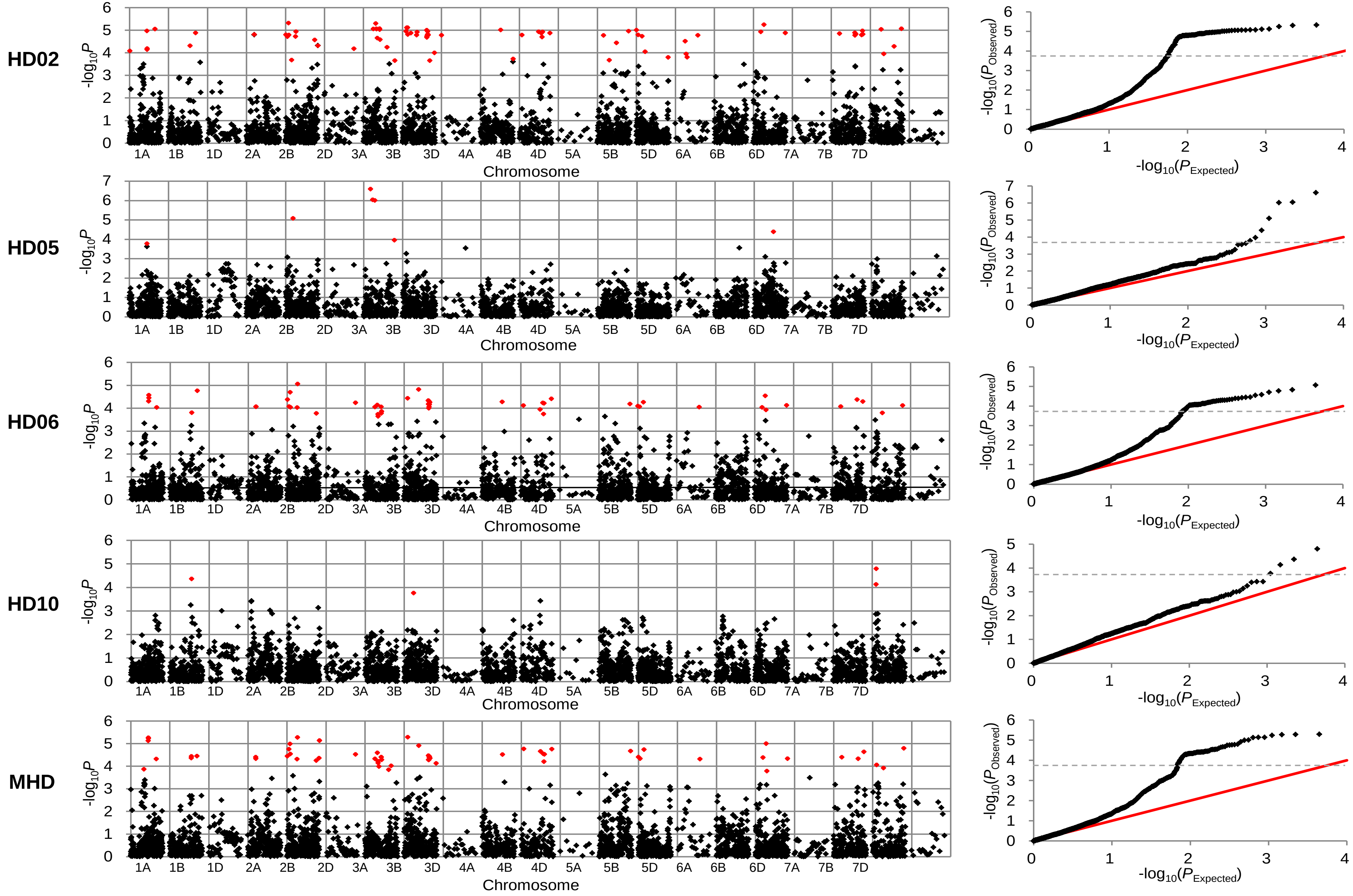

Supplementary Fig. S8 Manhattan and quantail-quantail plots for HD associated with SNPs in different environments on each chromosome, respectively. The red dots mean significant association signals in Manhattan figures.

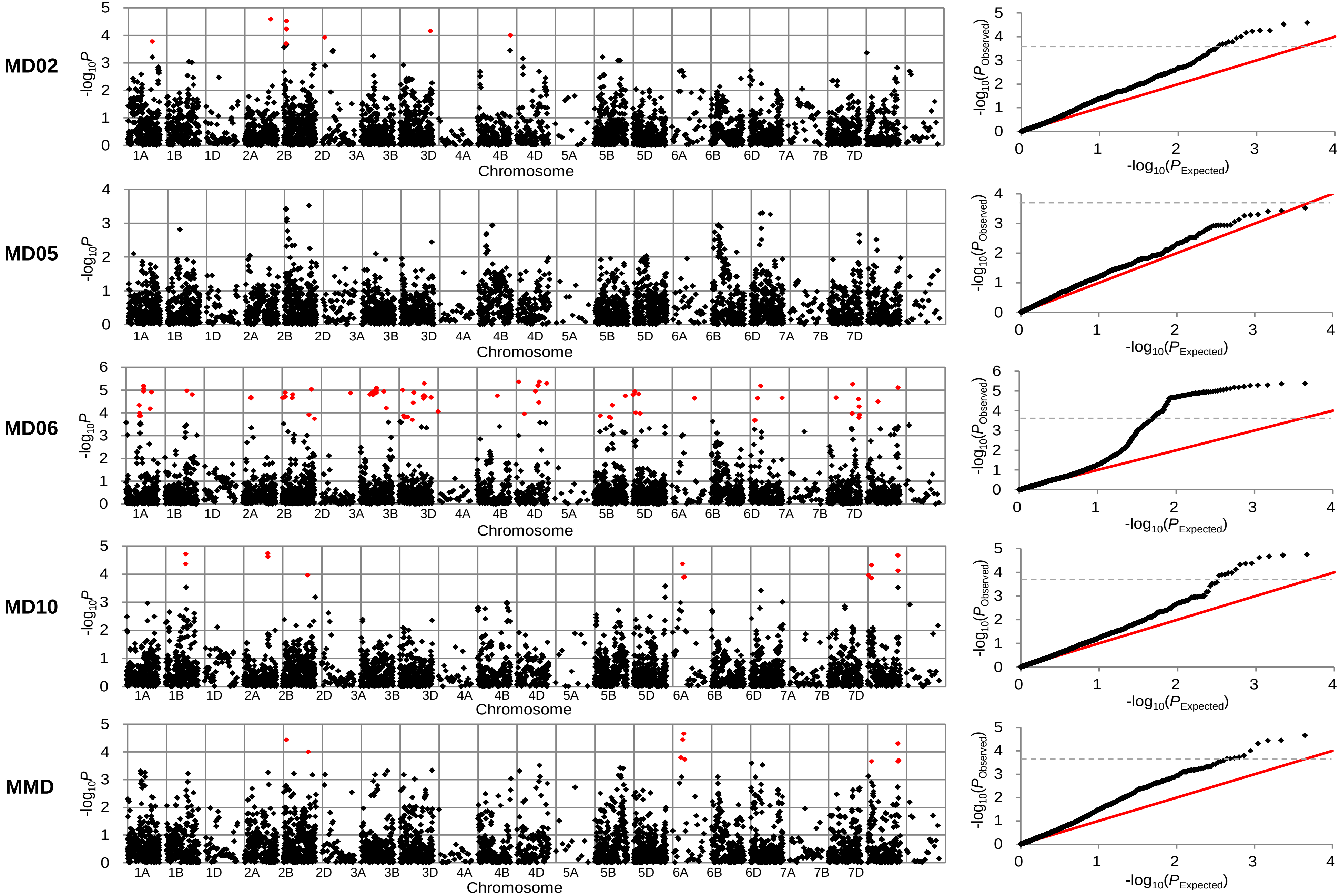

Supplementary Fig. S9 Manhattan and quantail-quantail plots for MD associated with SNPs in different environments on each chromosome, respectively. The red dots mean significant association signals in Manhattan figures.

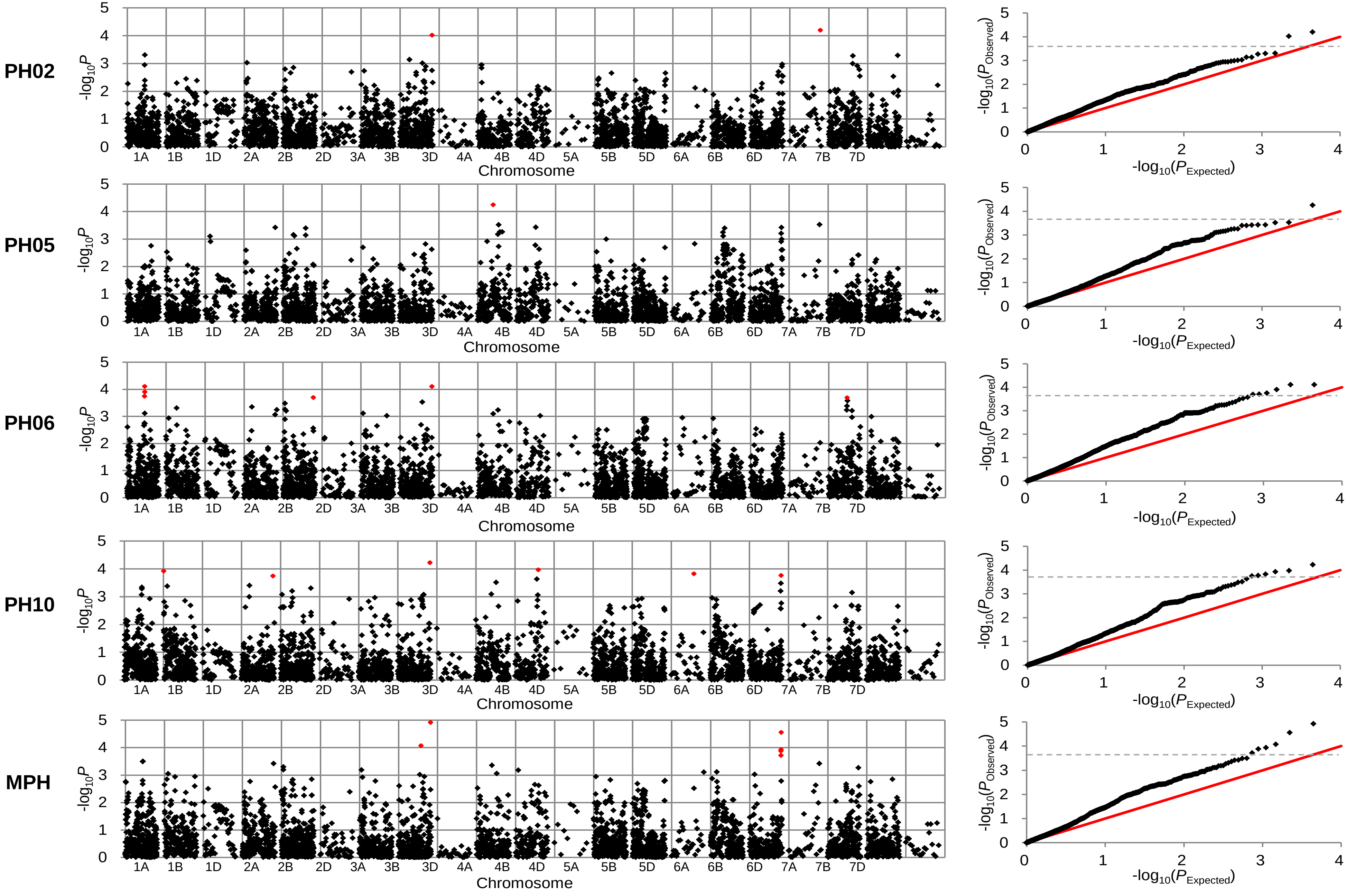

Supplementary Fig. S10 Manhattan and quantail-quantail plots for PH associated with SNPs in different environments on each chromosome, respectively. The red dots mean significant association signals in Manhattan figures.

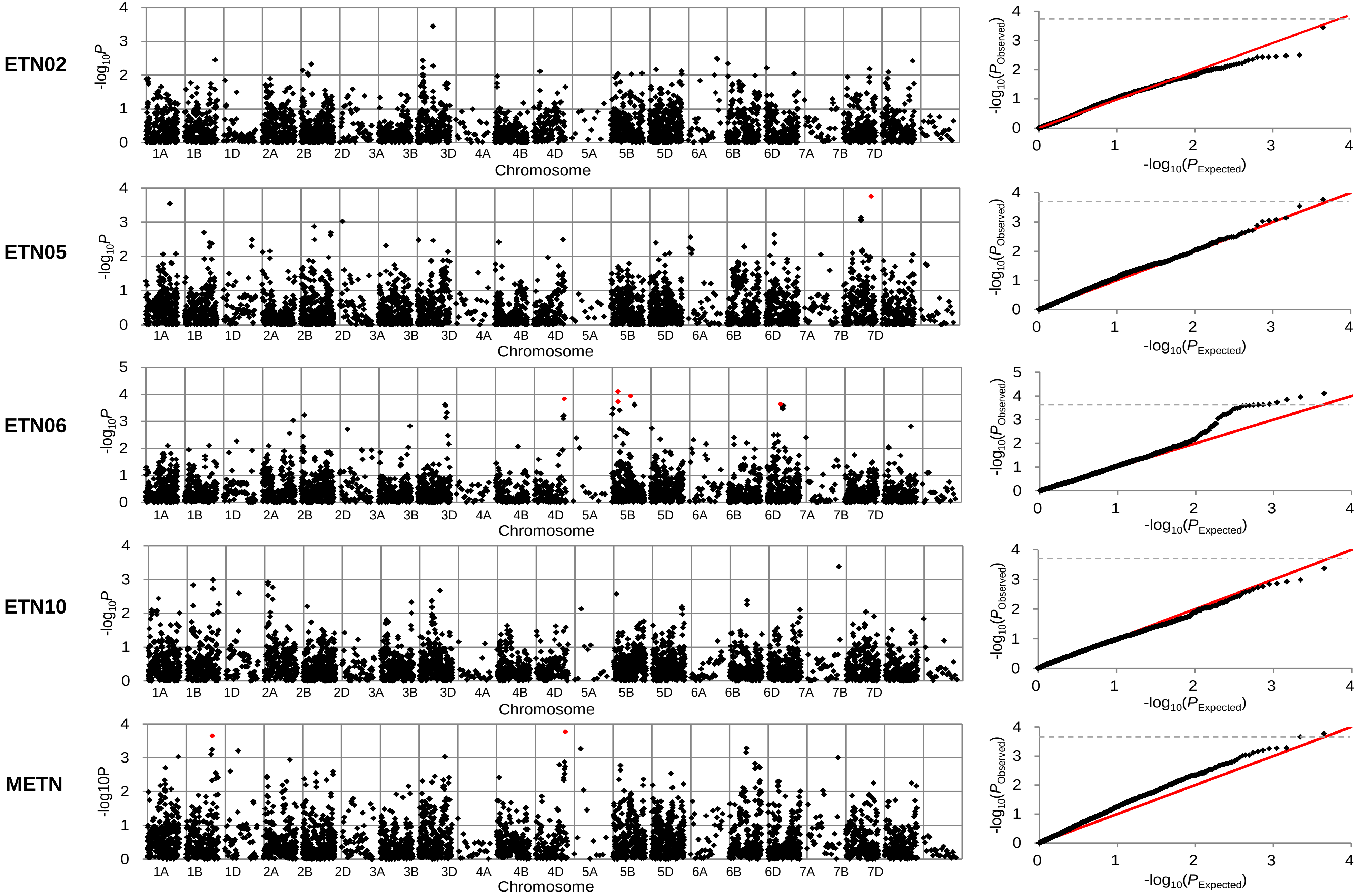

Supplementary Fig. S11 Manhattan and quantail-quantail plots for ETN associated with SNPs in different environments on each chromosome, respectively. The red dots mean significant association signals in Manhattan figures.

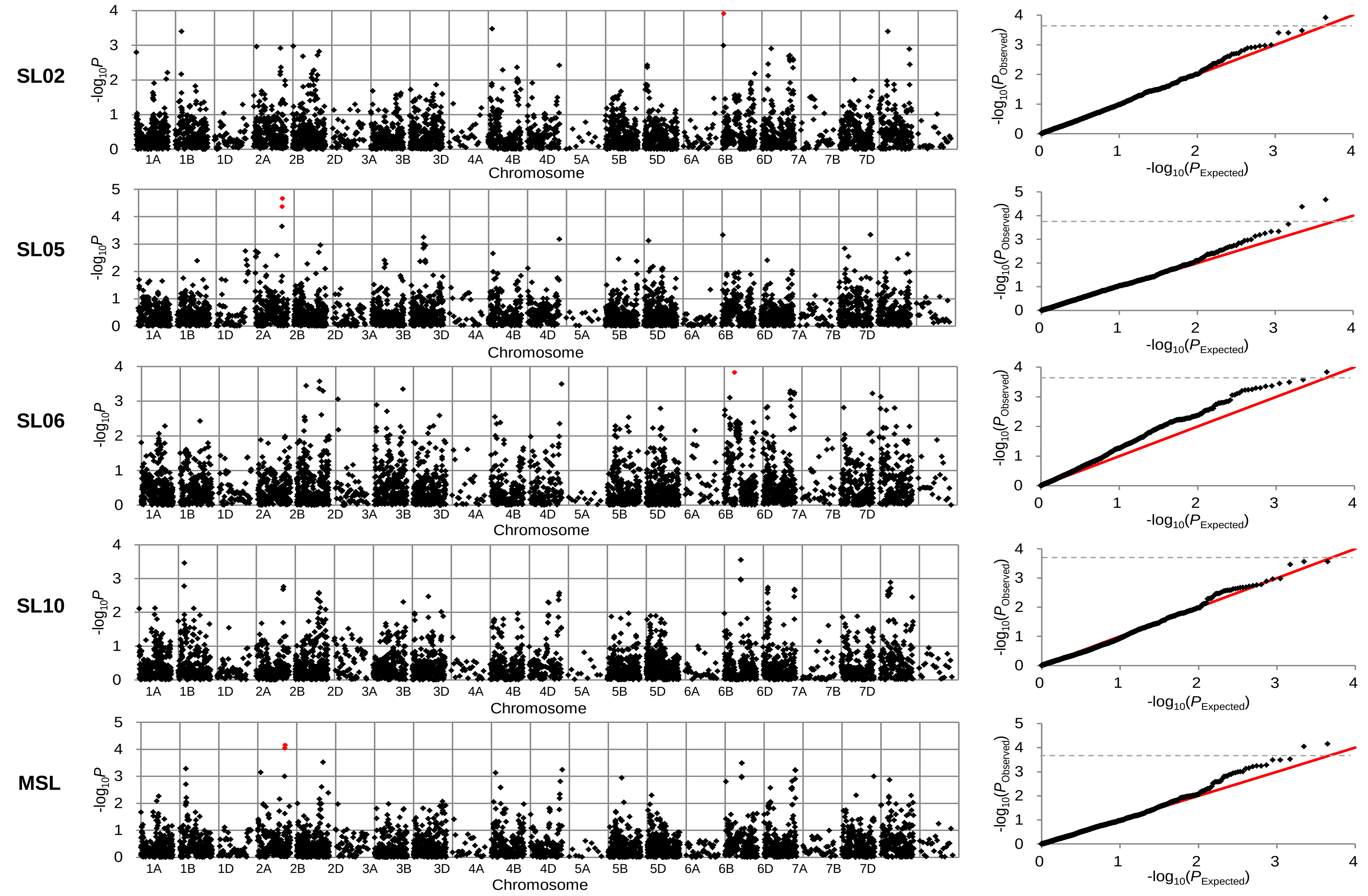

Supplementary Fig. S12 Manhattan and quantail-quantail plots for SL associated with SNPs in different environments on each chromosome, respectively. The red dots mean significant association signals in Manhattan figures.

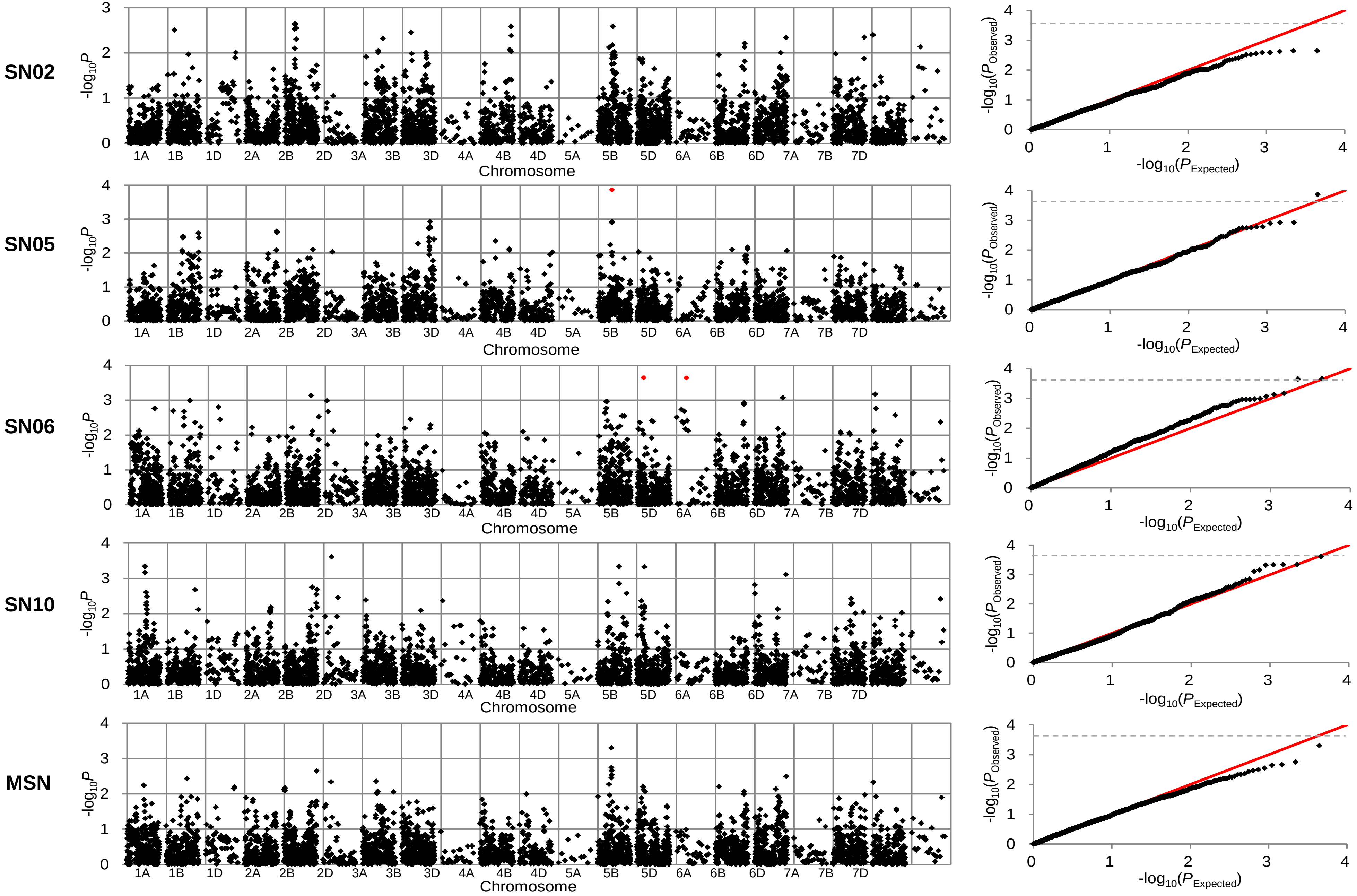

Supplementary Fig. S13 Manhattan and quantail-quantail plots for SN associated with SNPs in different environments on each chromosome, respectively. The red dots mean significant association signals in Manhattan figures.

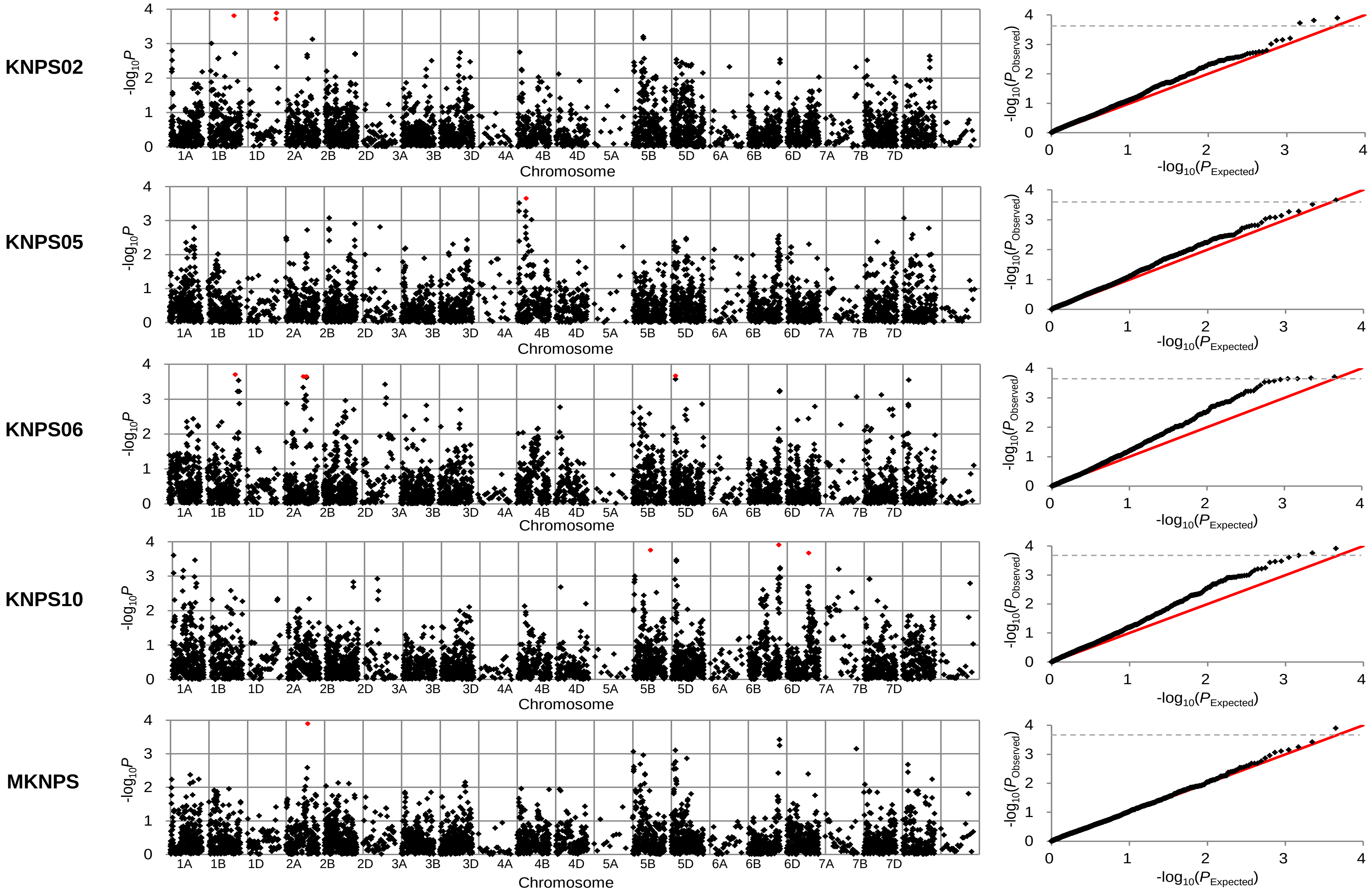

Supplementary Fig. S14 Manhattan and quantail-quantail plots for KNPS associated with SNPs in different environments on each chromosome, respectively. The red dots mean significant association signals in Manhattan figures.

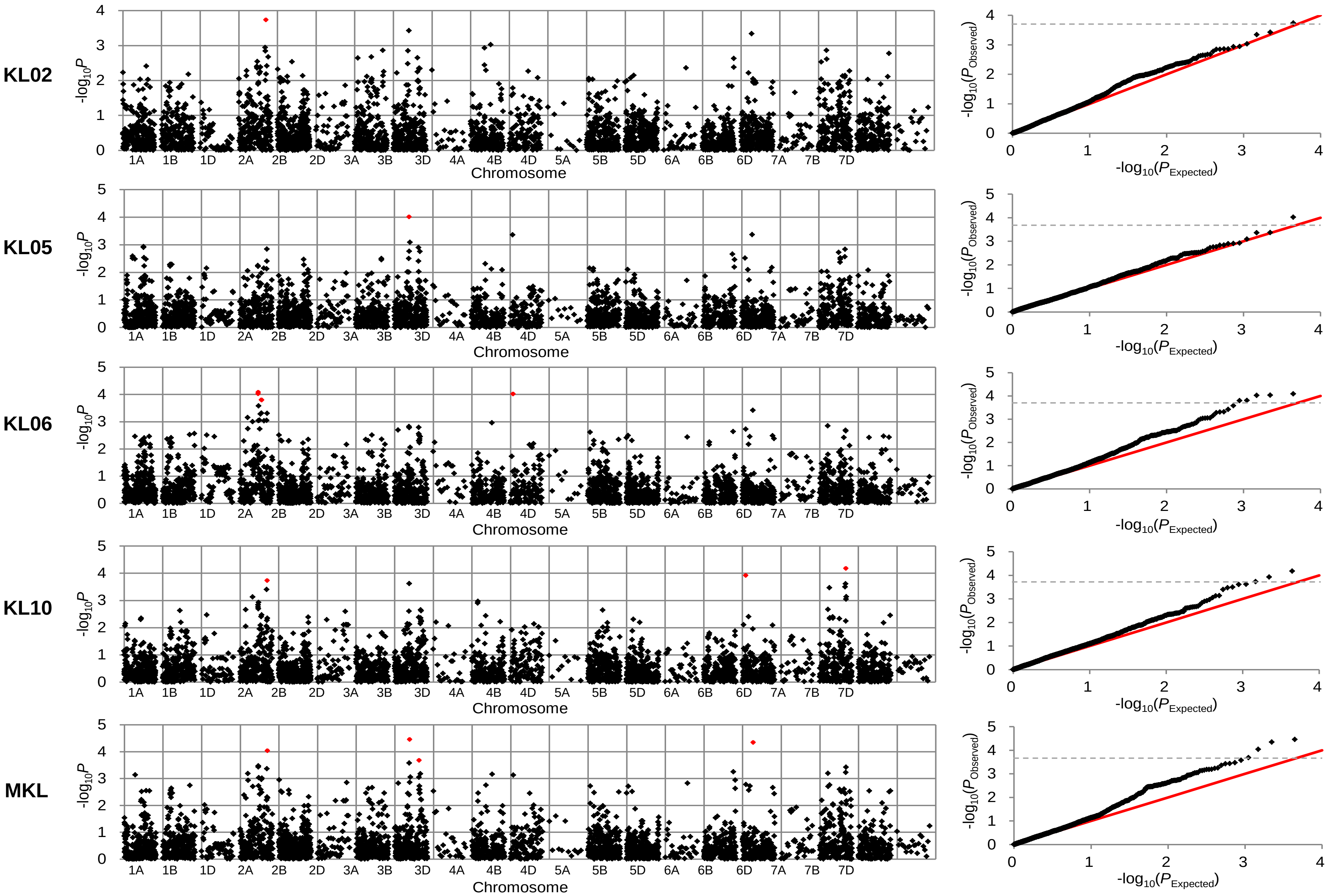

Supplementary Fig. S15 Manhattan and quantail-quantail plots for KL associated with SNPs in different environments on each chromosome, respectively. The red dots mean significant association signals in Manhattan figures.

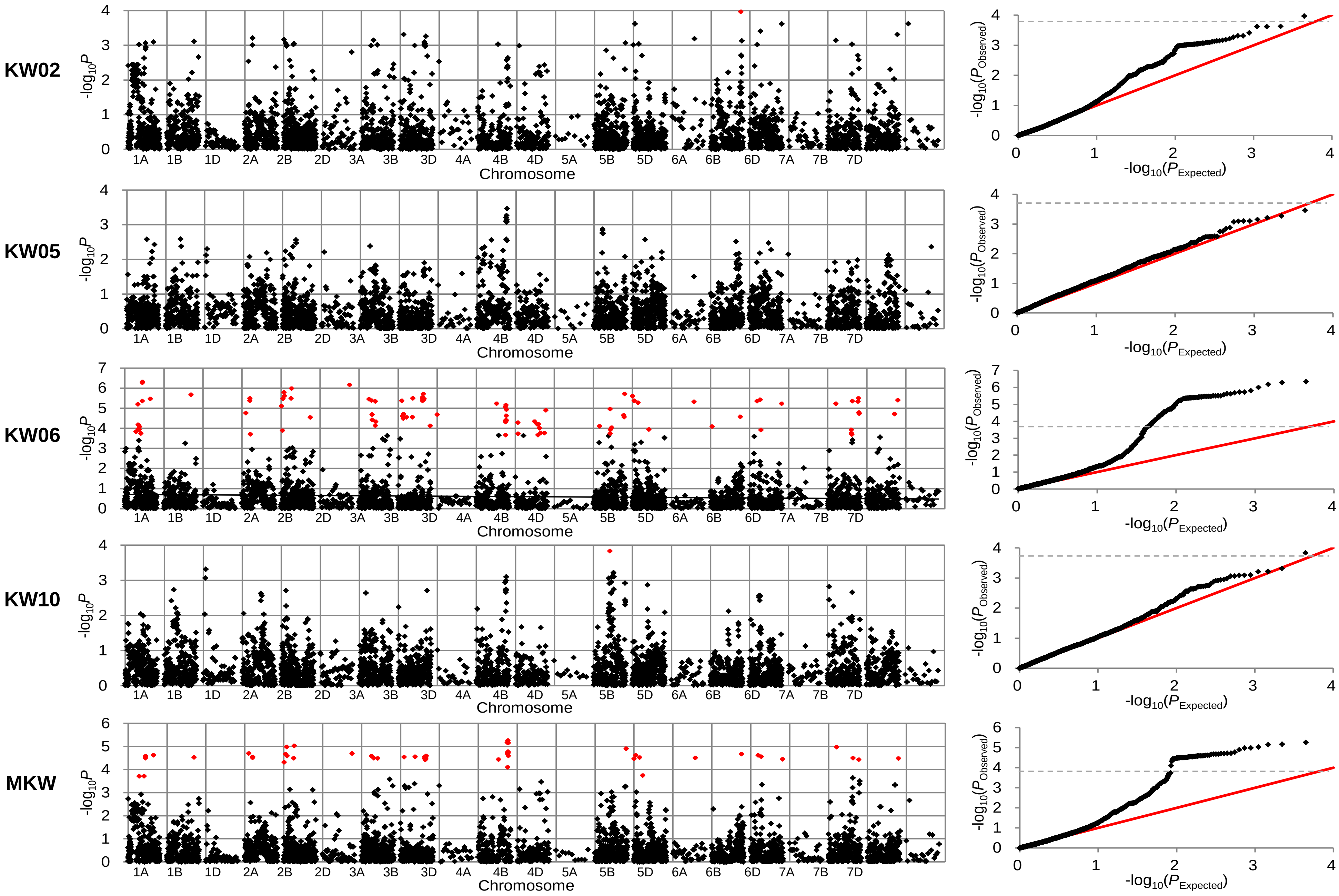

Supplementary Fig. S16 Manhattan and quantail-quantail plots for KW associated with SNPs in different environments on each chromosome, respectively. The red dots mean significant association signals in Manhattan figures.

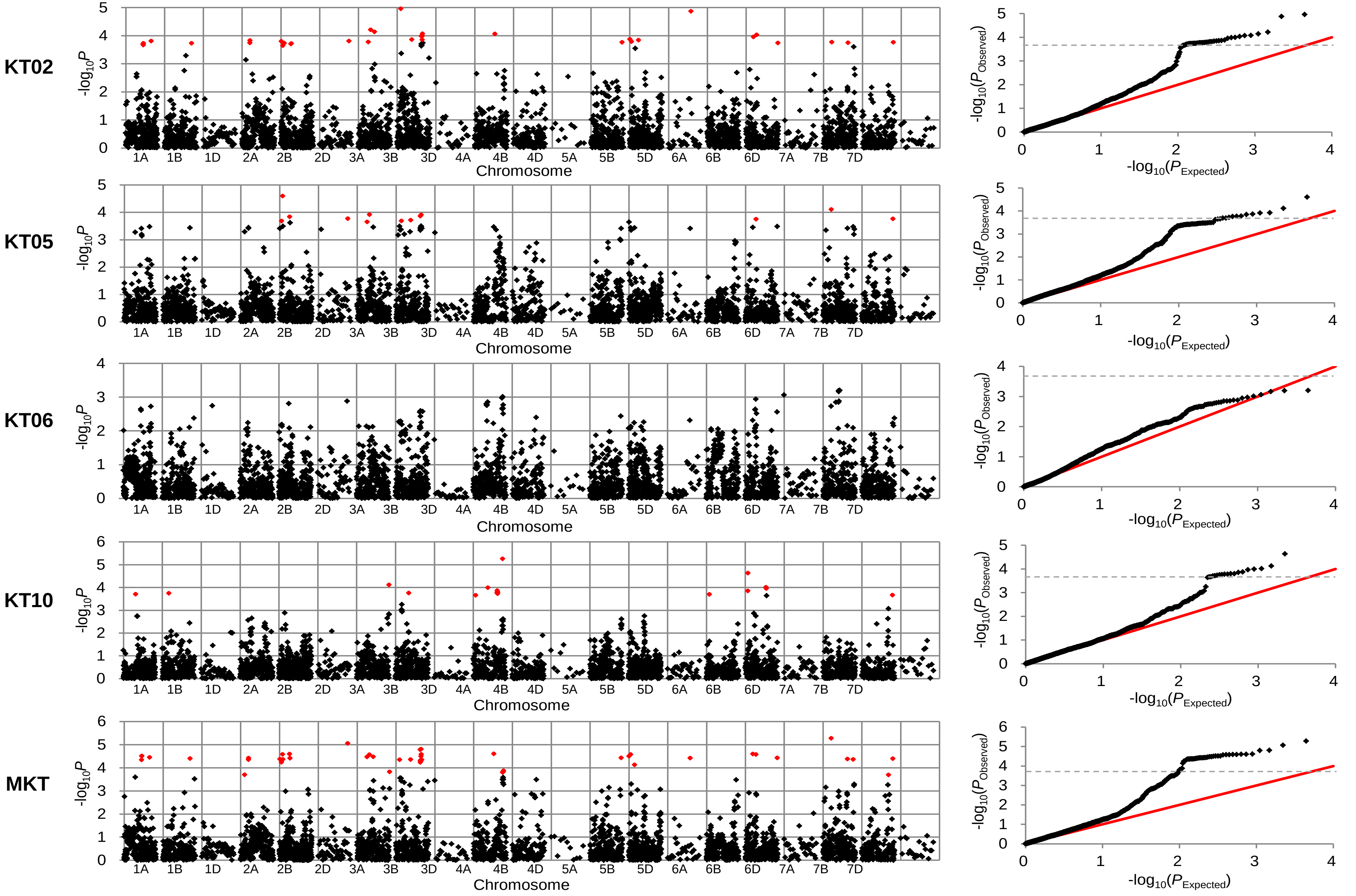

Supplementary Fig. S17 Manhattan and quantail-quantail plots for KT associated with SNPs in different environments on each chromosome, respectively. The red dots mean significant association signals in Manhattan figures.

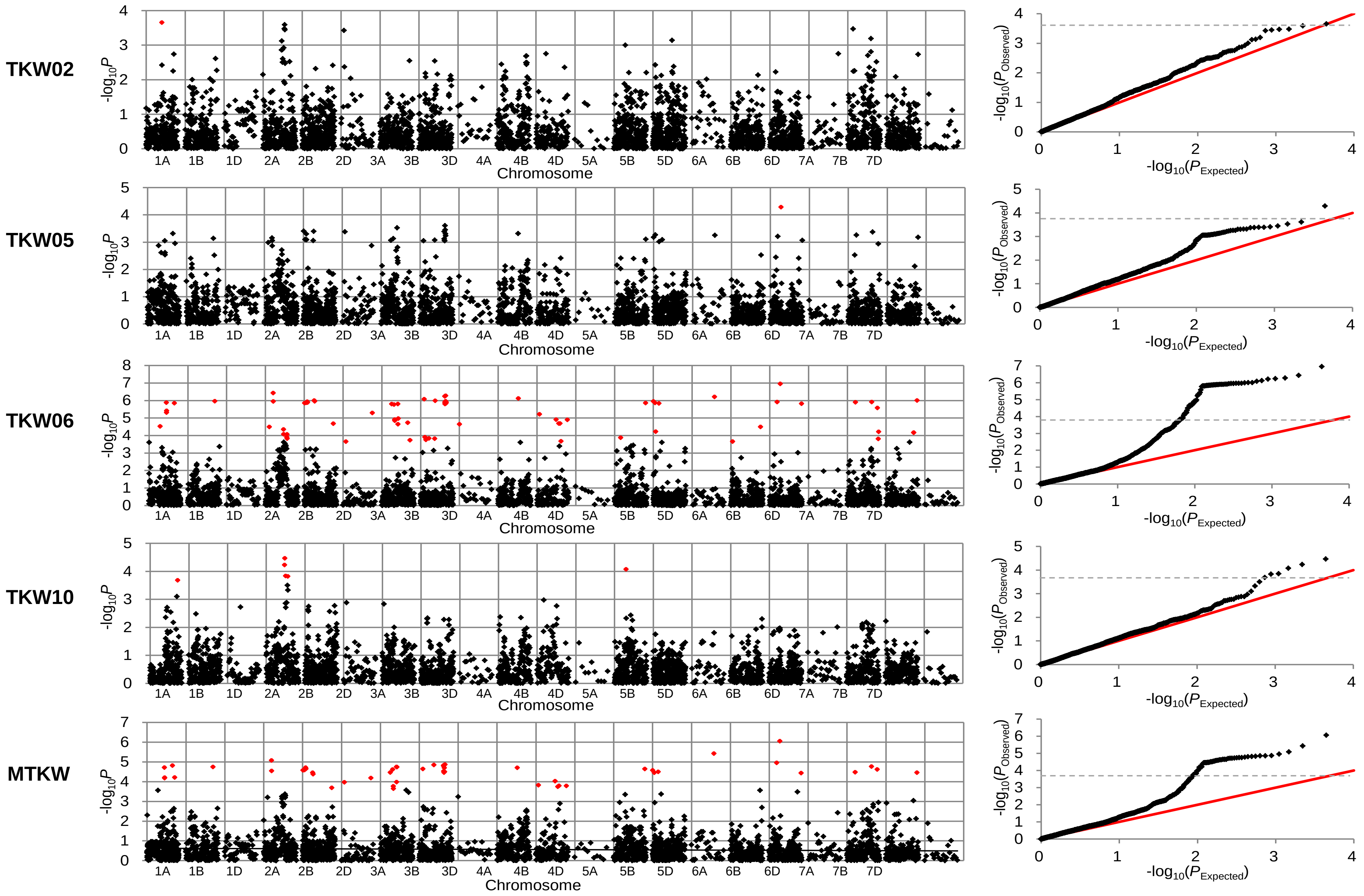

Supplementary Fig. S18 Manhattan and quantail-quantail plots for TKW associated with SNPs in different environments on each chromosome, respectively. The red dots mean significant association signals in Manhattan figures.

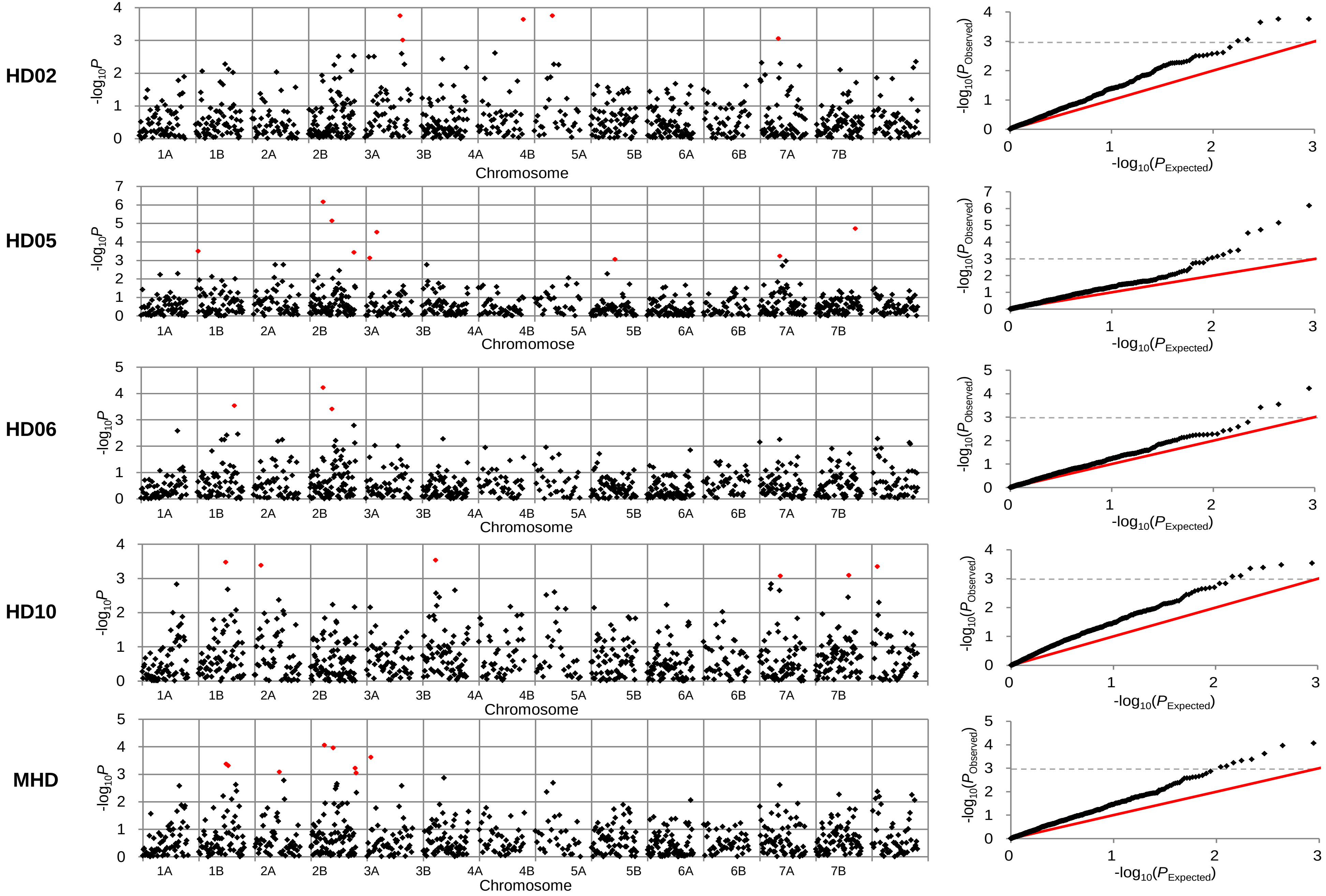

Supplementary Fig. S19 Manhattan and quantail-quantail plots for HD associated with haplotypes in different environments in the A and the B genome chromosomes, respectively. The red dots mean significant association signals in Manhattan figures.

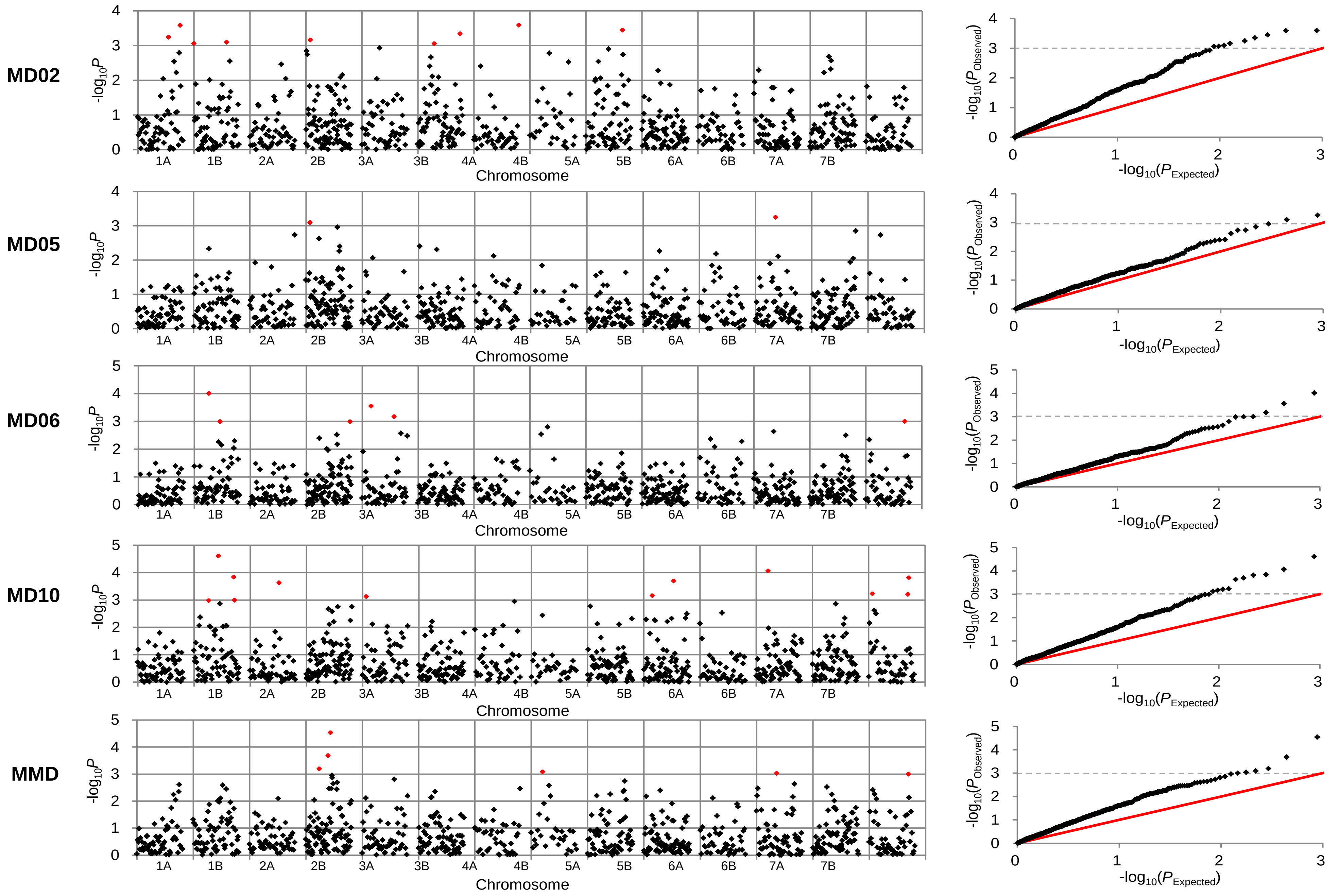

Supplementary Fig. S20 Manhattan and quantail-quantail plots for MD associated with haplotypes in different environments in the A and the B genome chromosomes, respectively. The red dots mean significant association signals in Manhattan figures.

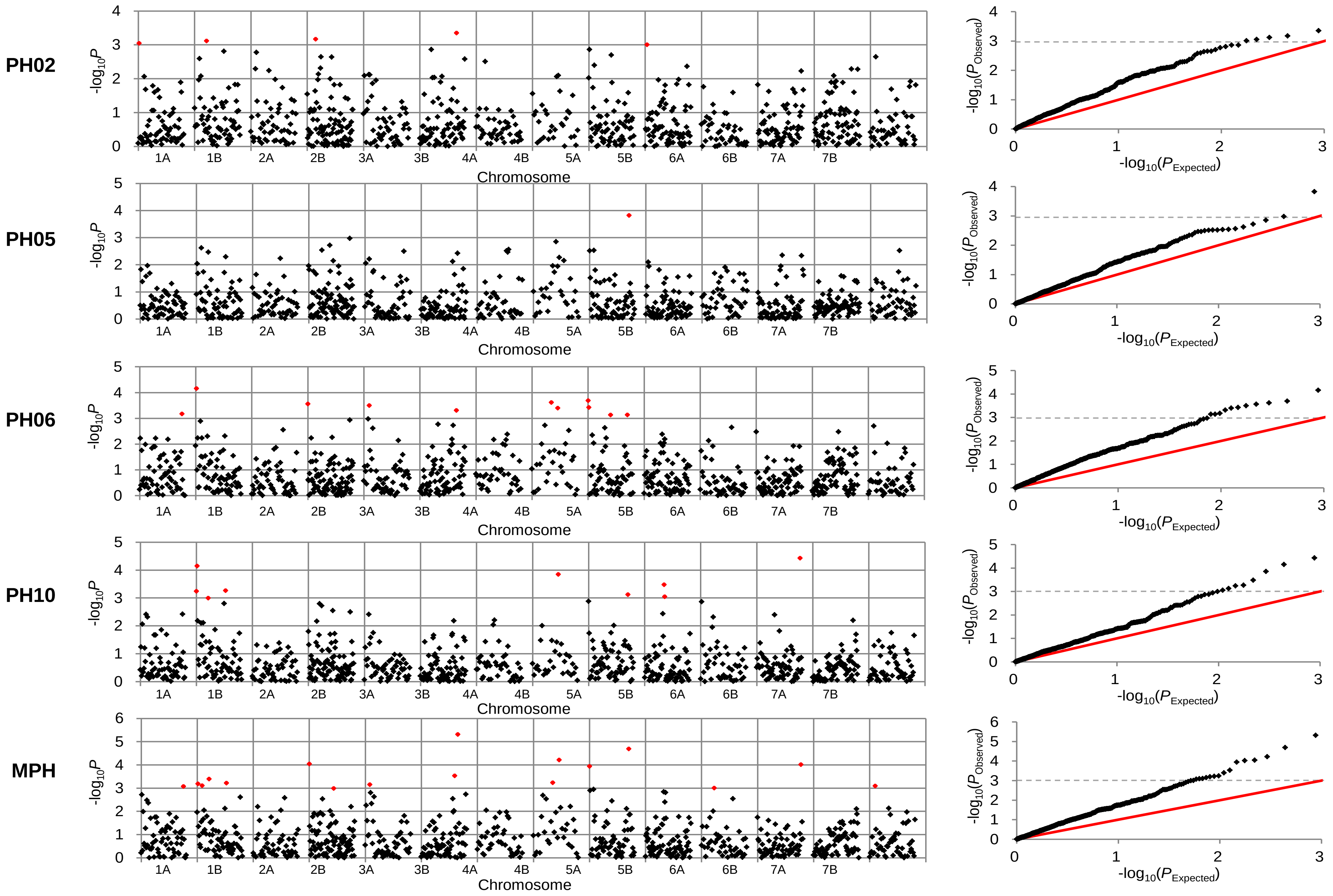

Supplementary Fig. S21 Manhattan and quantail-quantail plots for PH associated with haplotypes in different environments in the A and the B genome chromosomes, respectively. The red dots mean significant association signals in Manhattan figures.

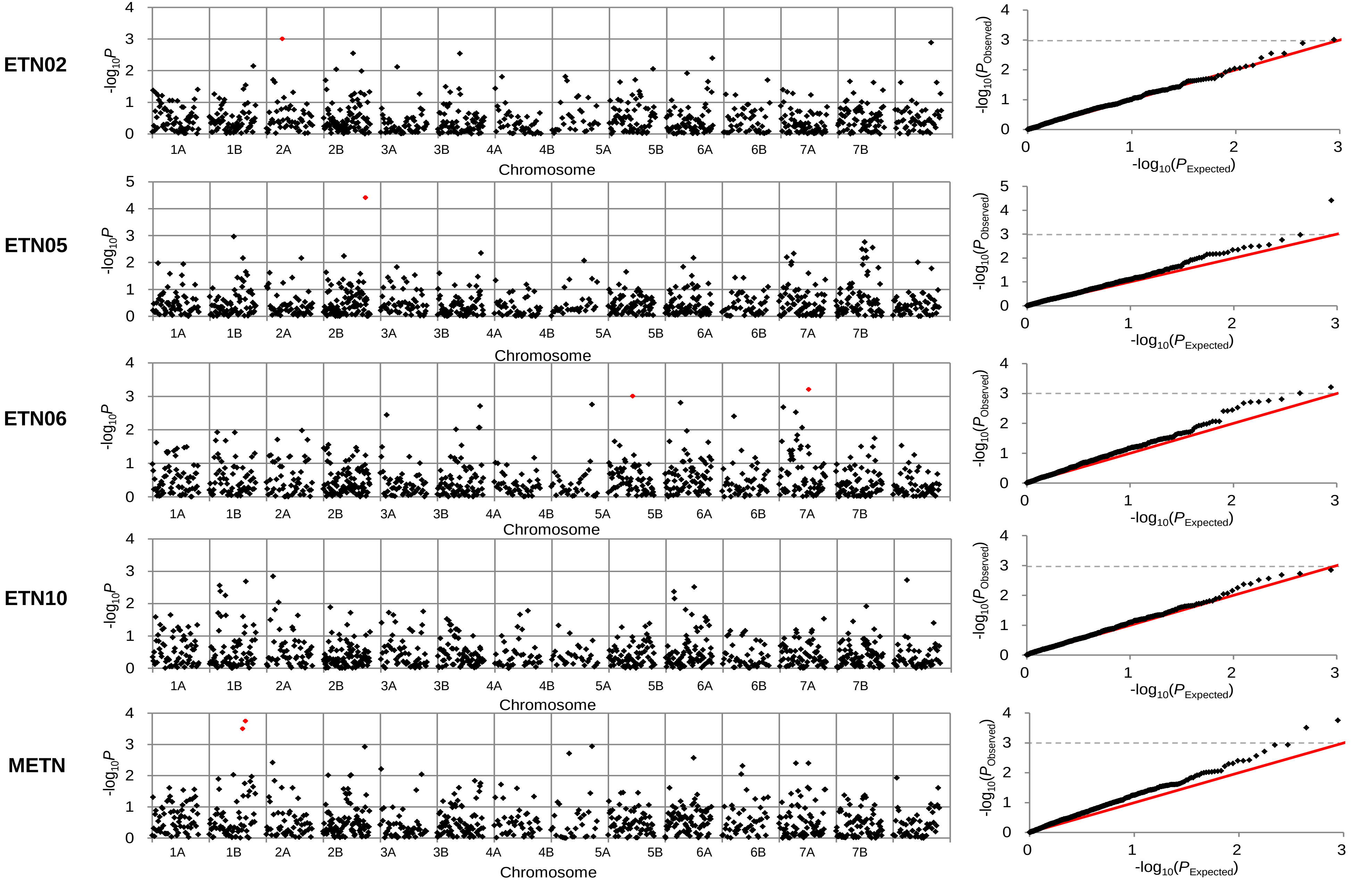

Supplementary Fig. S22 Manhattan and quantail-quantail plots for ETN associated with haplotypes in different environments in the A and the B genome chromosomes, respectively. The red dots mean significant association signals in Manhattan figures.

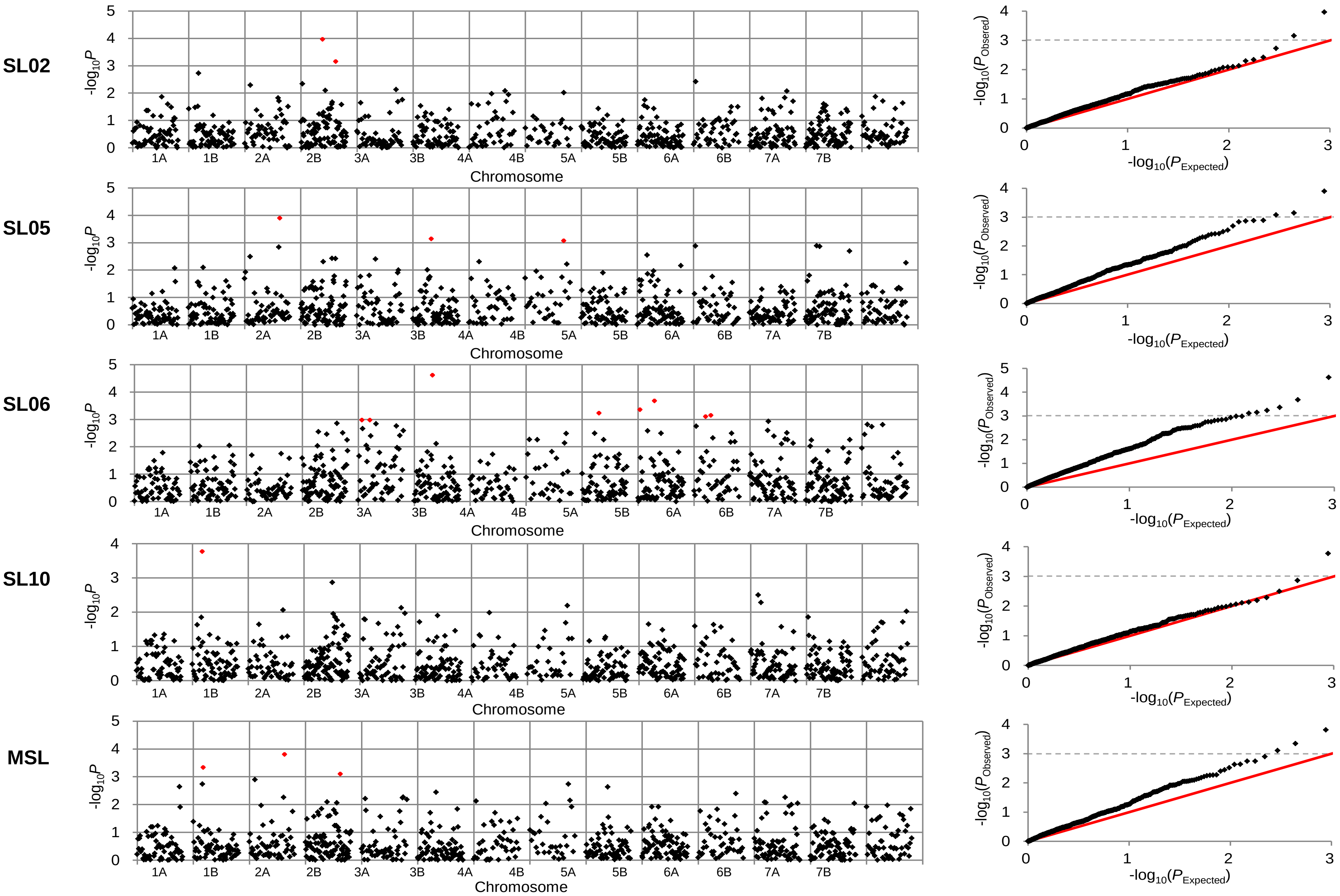

Supplementary Fig. S23 Manhattan and quantail-quantail plots for SL associated with haplotypes in different environments in the A and the B genome chromosomes, respectively. The red dots mean significant association signals in Manhattan figures.

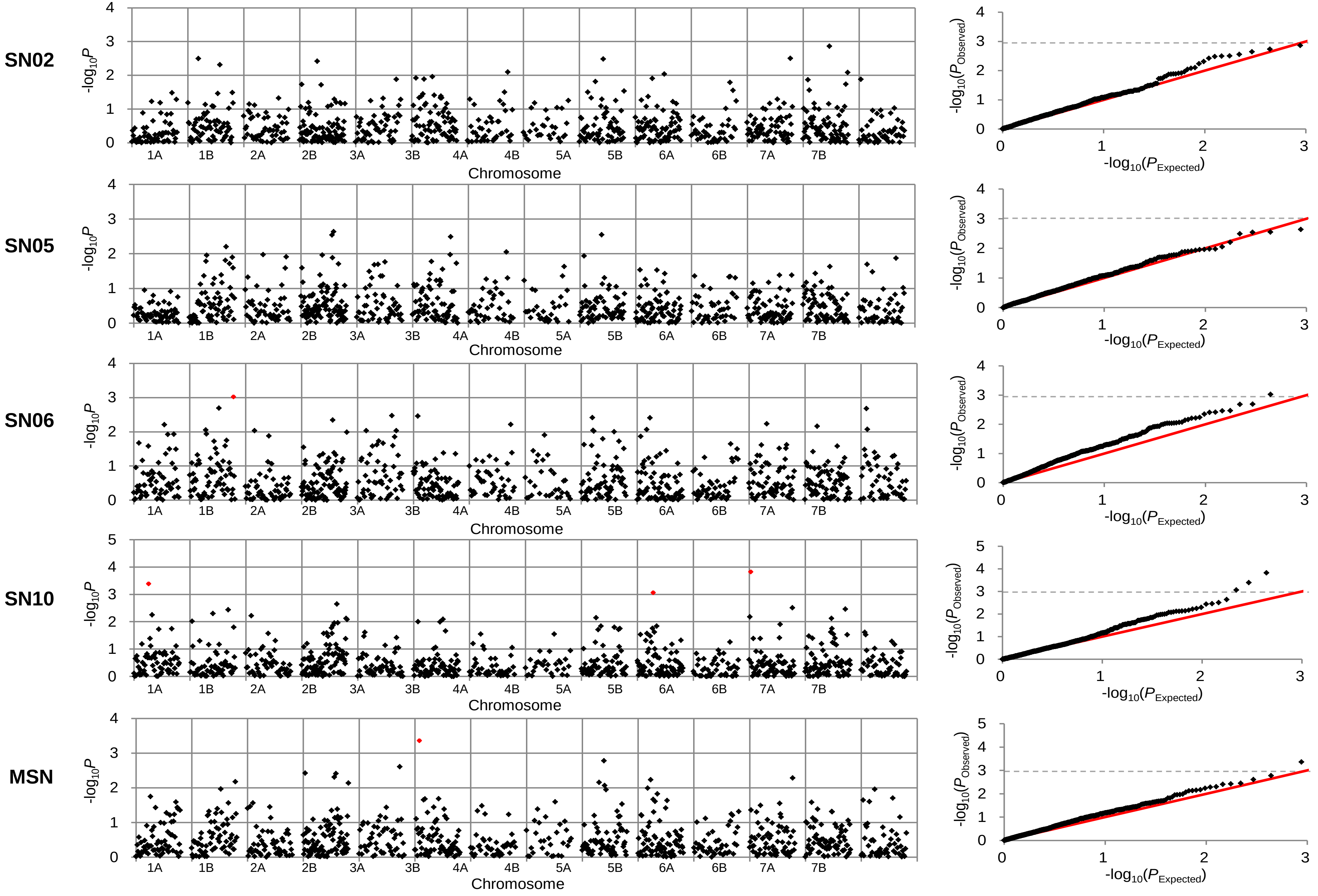

Supplementary Fig. S24 Manhattan and quantail-quantail plots for SN associated with haplotypes in different environments in the A and the B genome chromosomes, respectively. The red dots mean significant association signals in Manhattan figures.

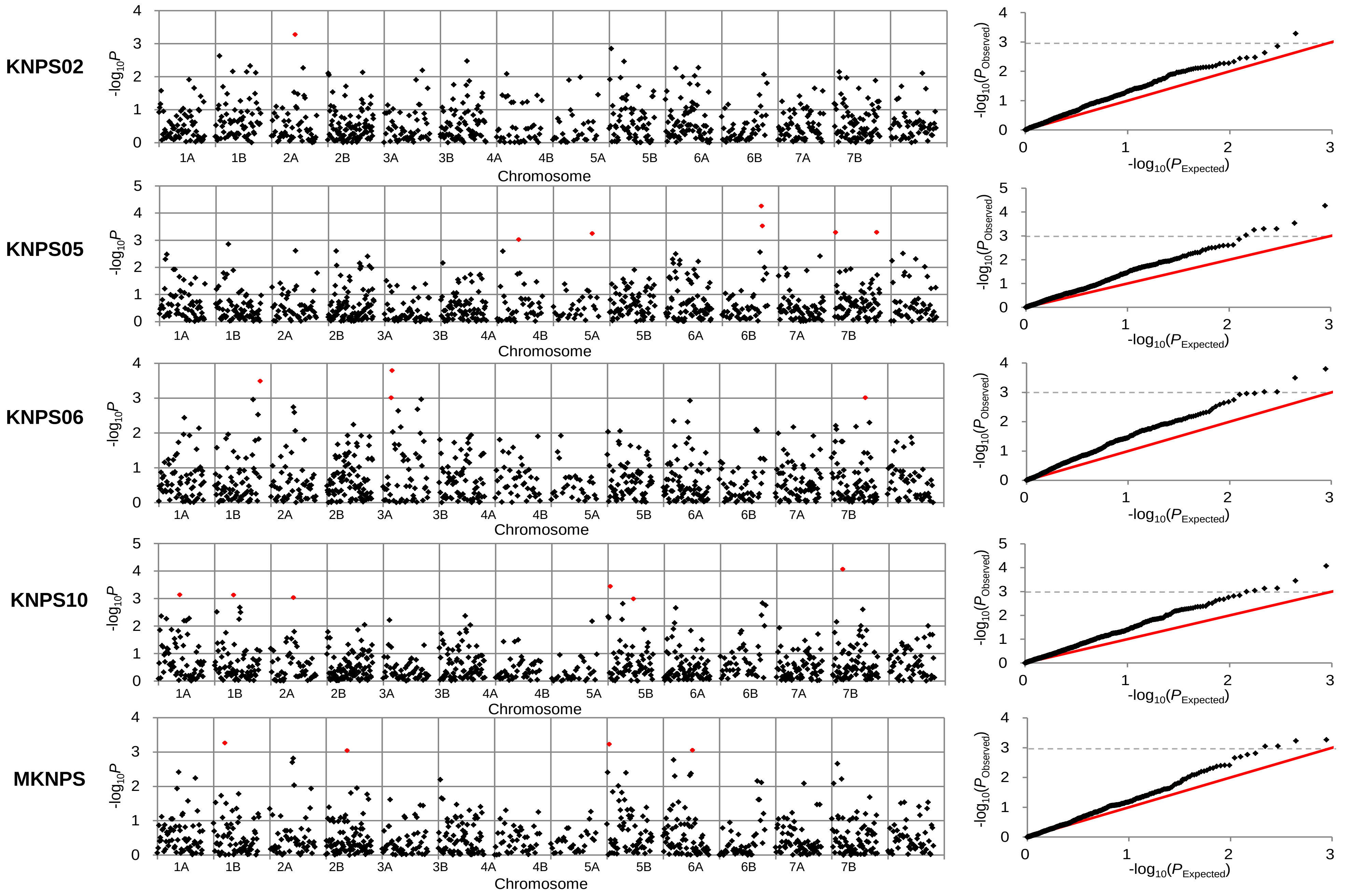

Supplementary Fig. S25 Manhattan and quantail-quantail plots for KNPS associated with haplotypes in different environments in the A and the B genome chromosomes, respectively. The red dots mean significant association signals in Manhattan figures.

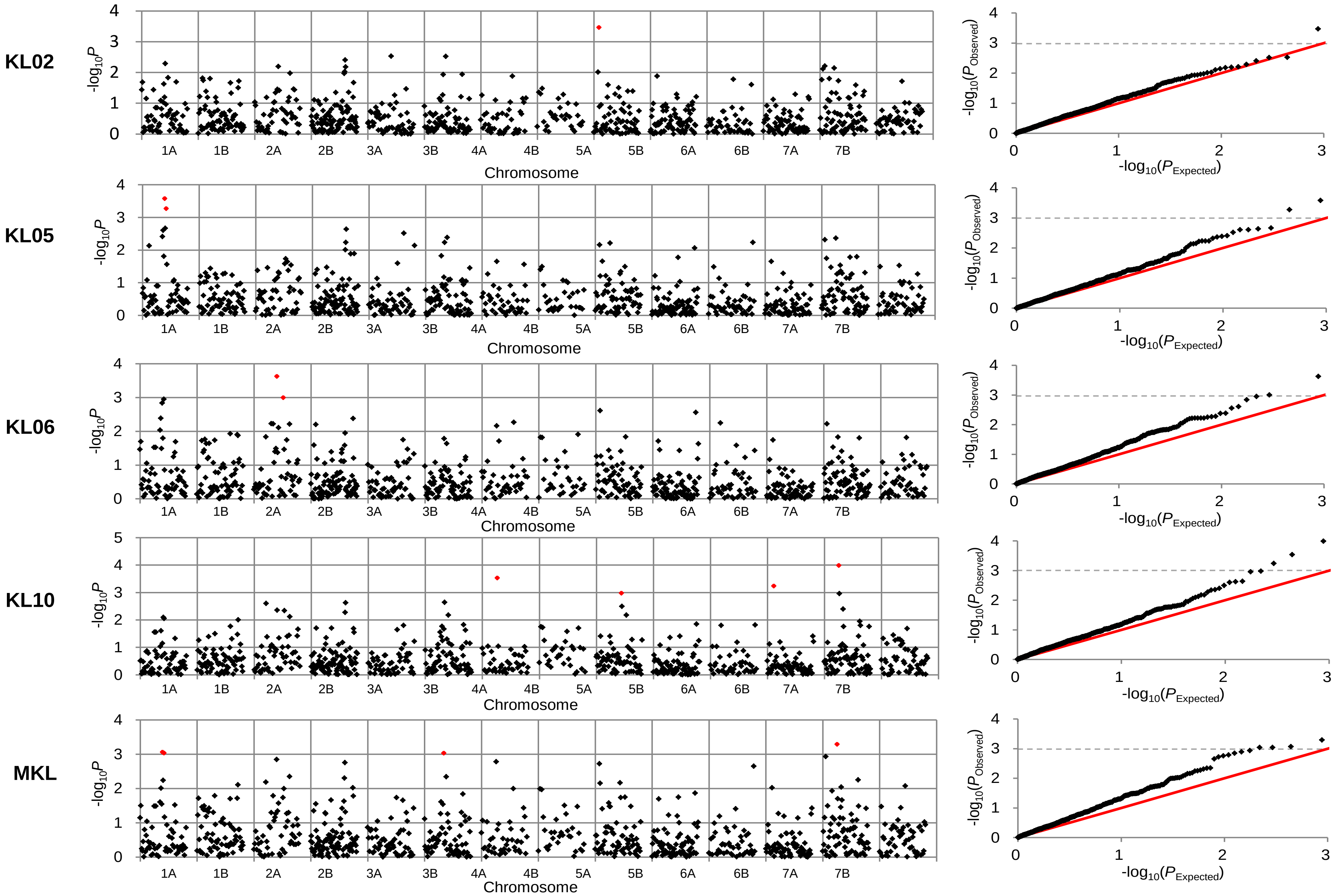

Supplementary Fig. S26 Manhattan and quantail-quantail plots for KL associated with haplotypes in different environments in the A and the B genome chromosomes, respectively. The red dots mean significant association signals in Manhattan figures.

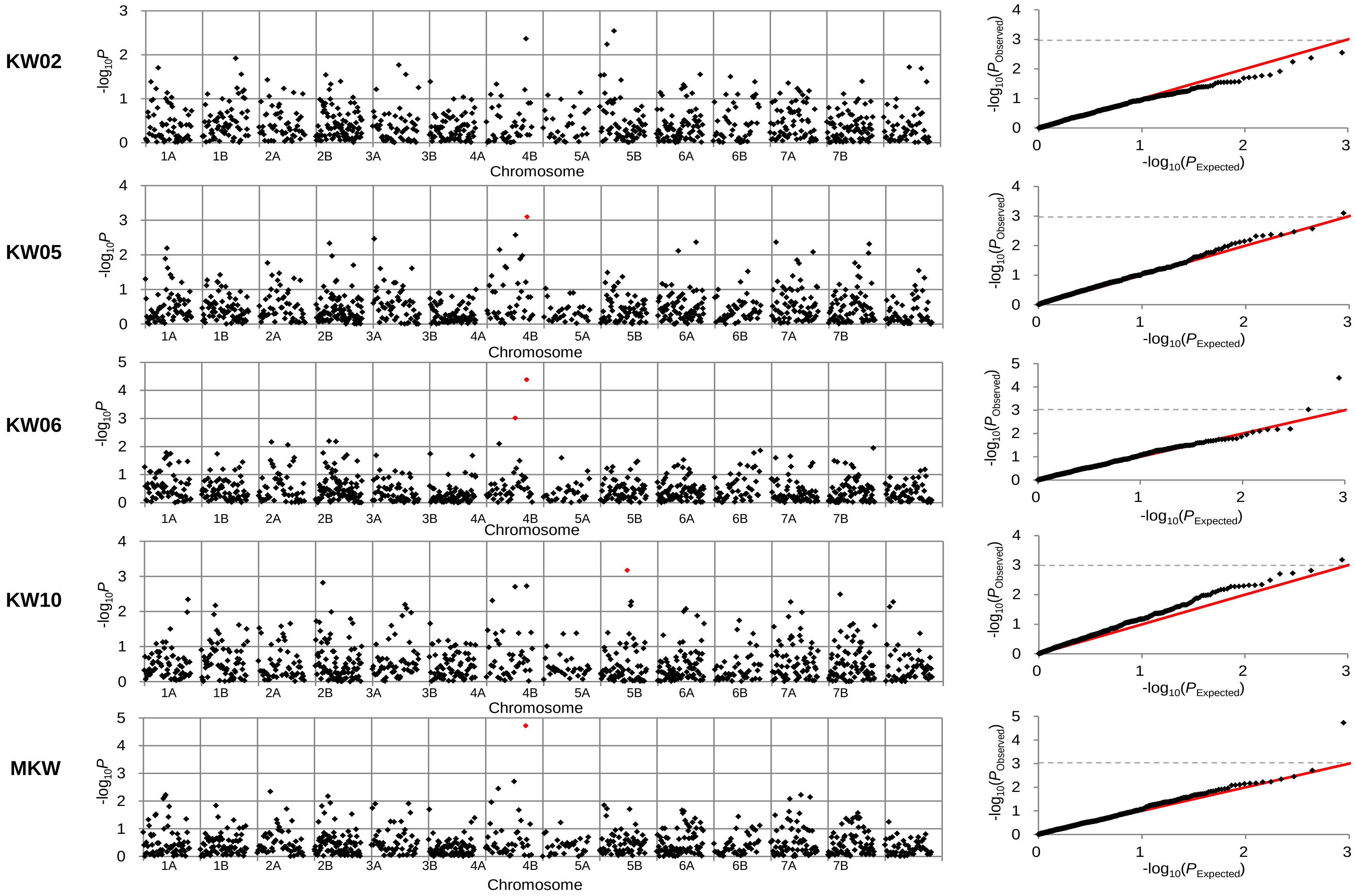

Supplementary Fig. S27 Manhattan and quantail-quantail plots for KW associated with haplotypes in different environments in the A and the B genome chromosomes, respectively. The red dots mean significant association signals in Manhattan figures.

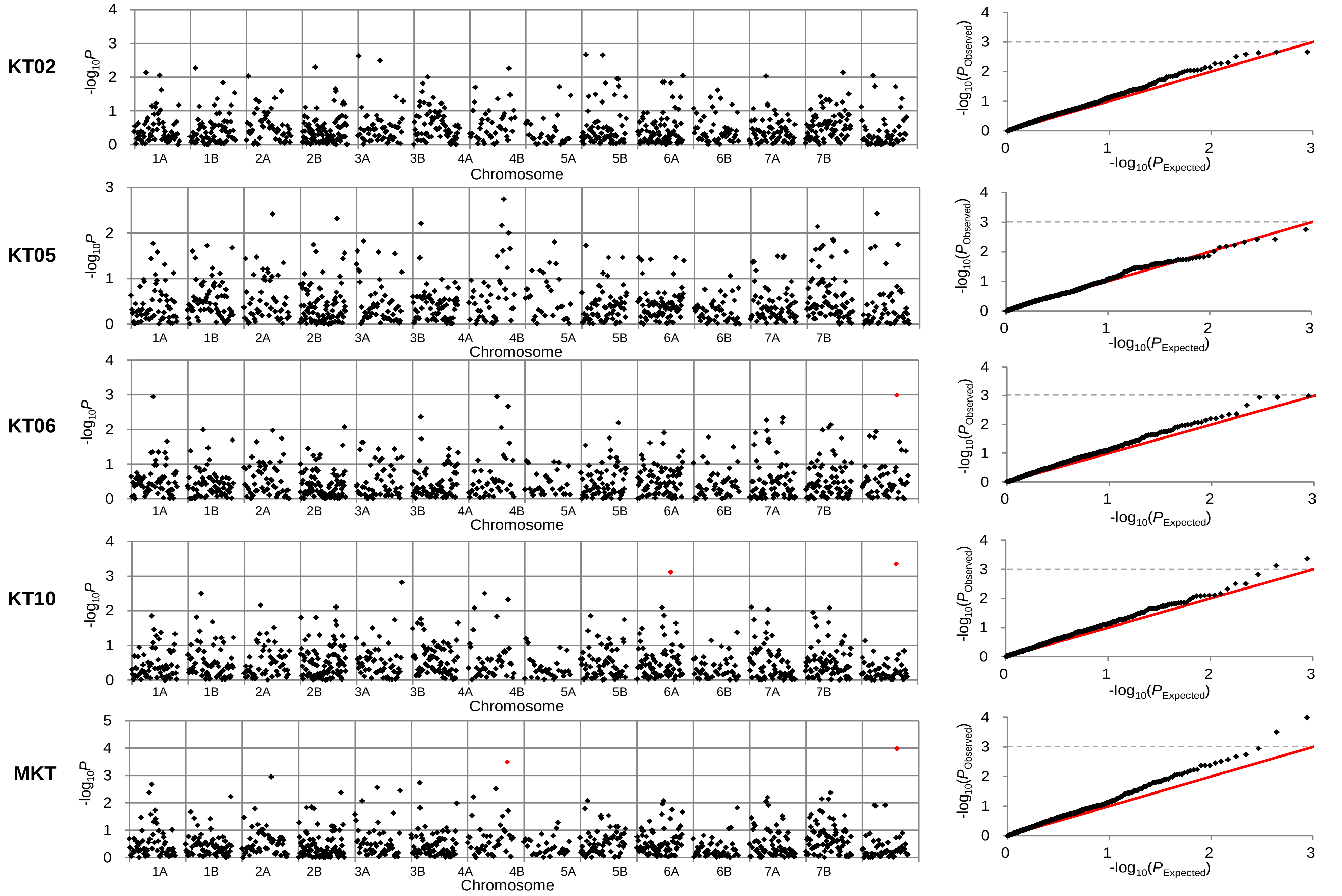

Supplementary Fig. S28 Manhattan and quantail-quantail plots for KT associated with haplotypes in different environments in the A and the B genome chromosomes, respectively. The red dots mean significant association signals in Manhattan figures.

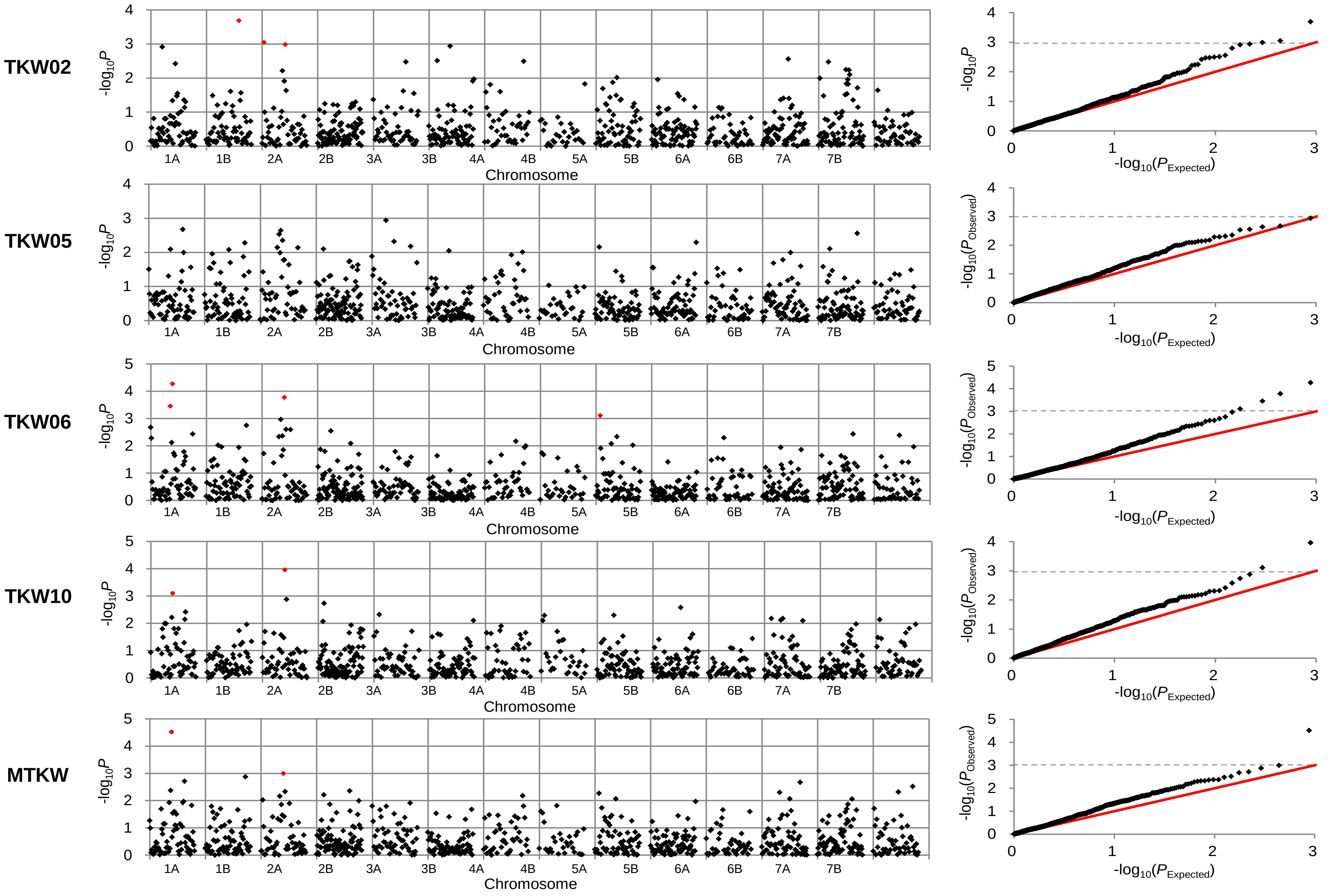

Supplementary Fig. S29 Manhattan and quantail-quantail plots for TKW associated with haplotypes in different environments in the A and the B genome chromosomes, respectively. The red dots mean significant association signals in Manhattan figures.

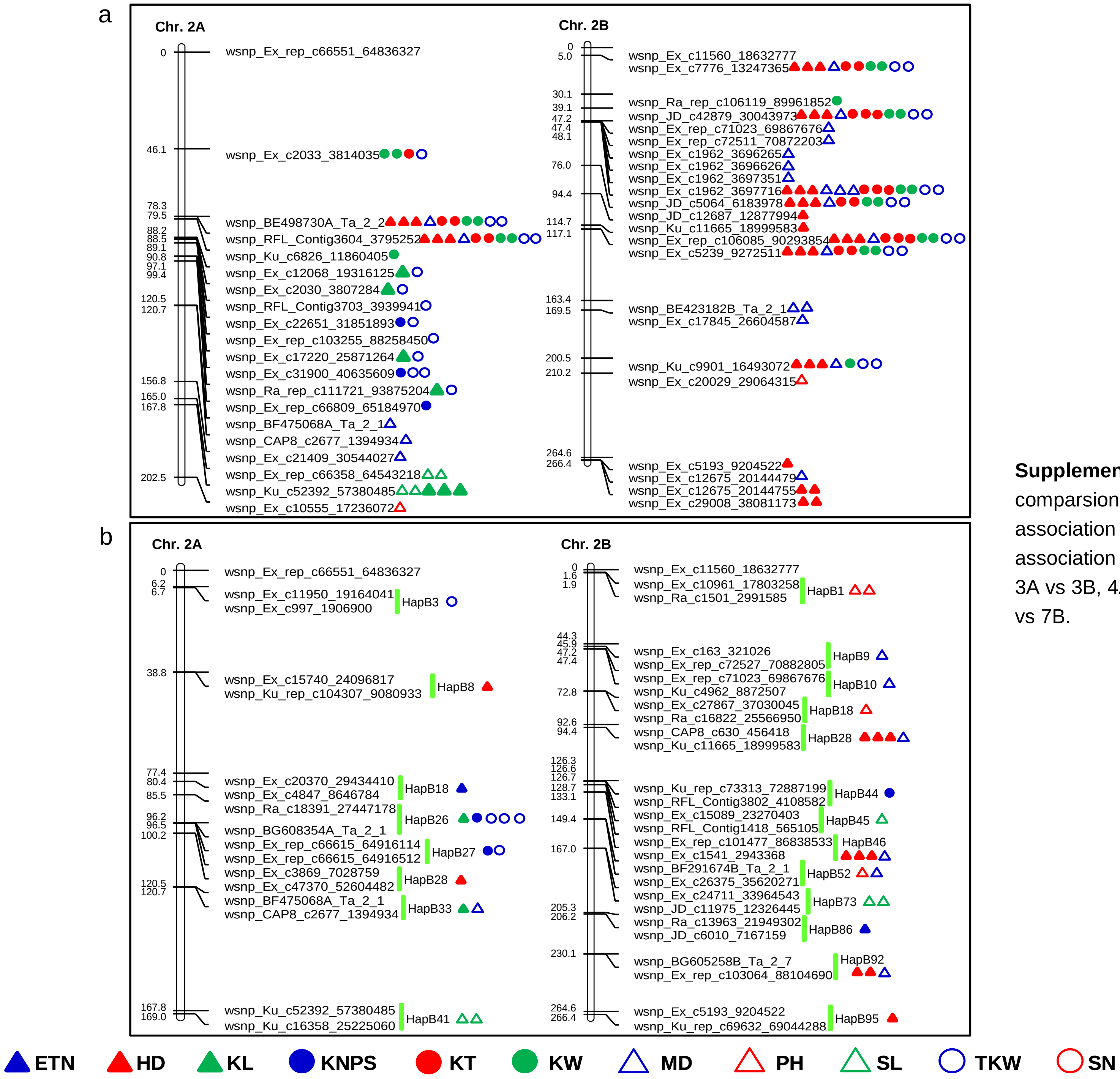

Supplementary Fig. S30 Effectiveness comparsion of single SNP-based association (a) with haplotype-based association (b) on chromosomes 2A vs 2B, 3A vs 3B, 4A vs 4B, 5A vs 5B, 6A vs 6B, 7A vs 7B.

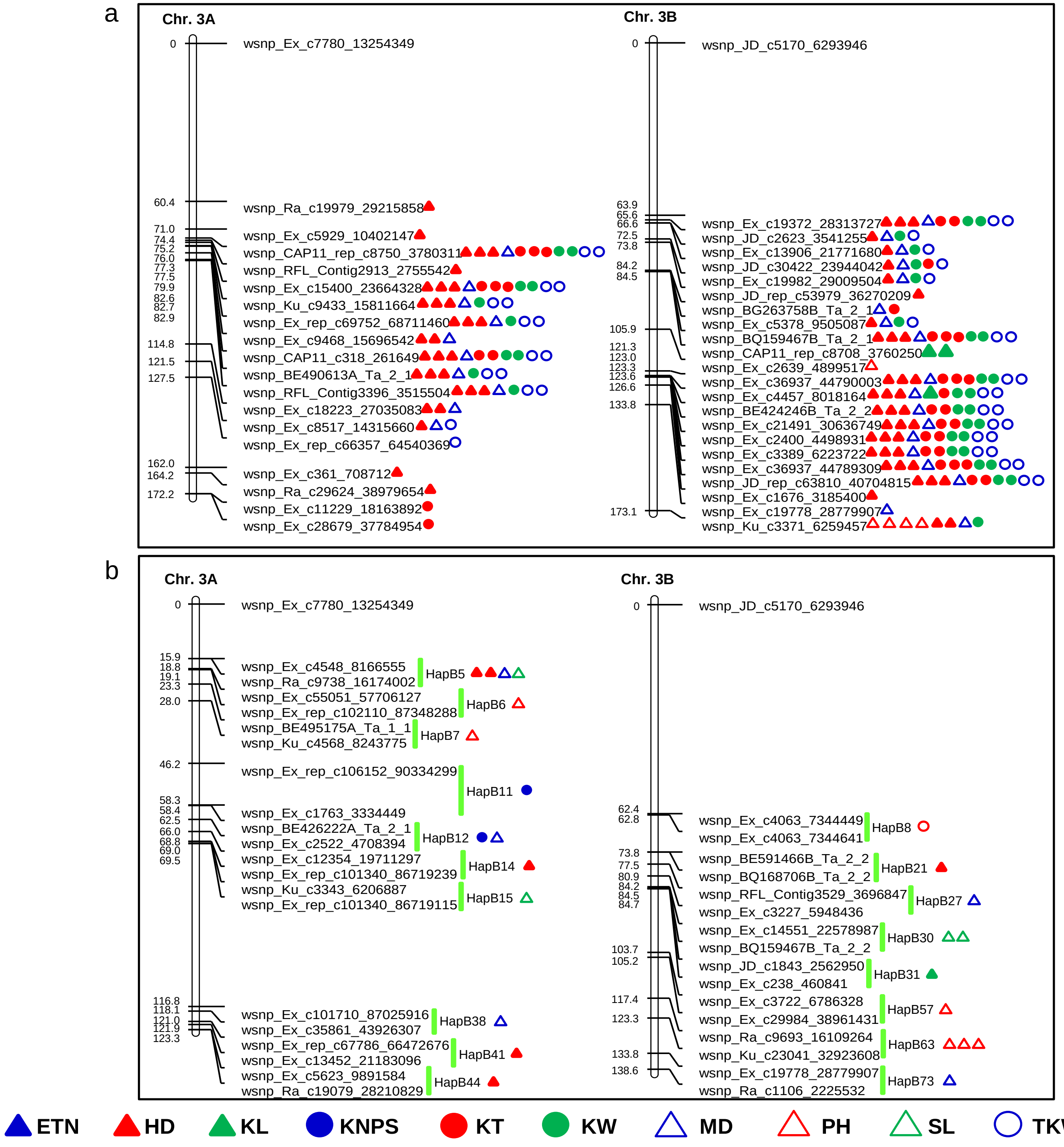

Supplementary Fig. S30_continued Effectiveness comparsion of single SNP-based association (a) with haplotype-based association (b) on chromosomes 2A vs 2B, 3A vs 3B, 4A vs 4B, 5A vs 5B, 6A vs 6B, 7A vs 7B.

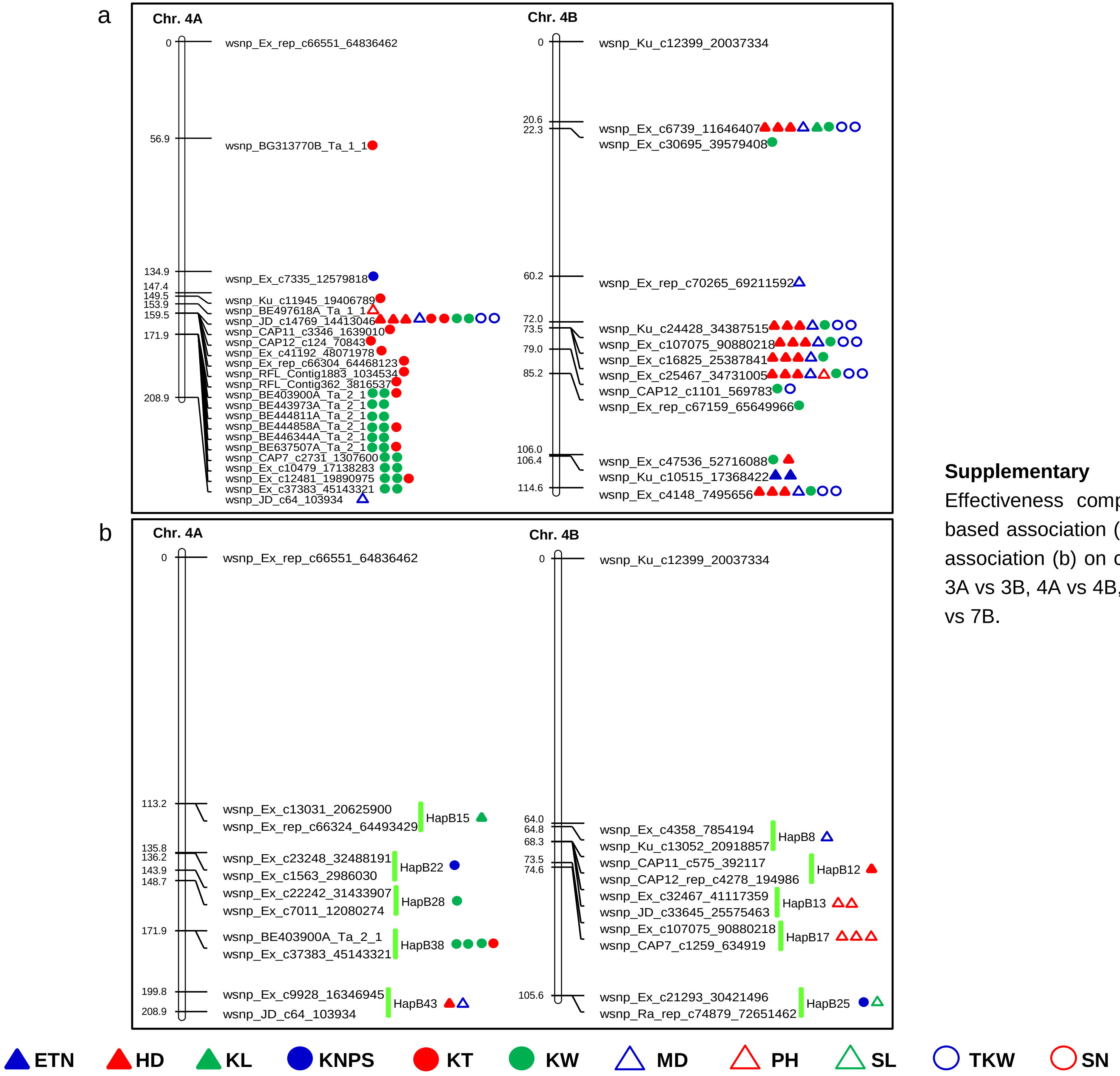

Supplementary Fig. S30_continued Effectiveness comparsion of single SNP-based association (a) with haplotype-based association (b) on chromosomes 2A vs 2B, 3A vs 3B, 4A vs 4B, 5A vs 5B, 6A vs 6B, 7A vs 7B.

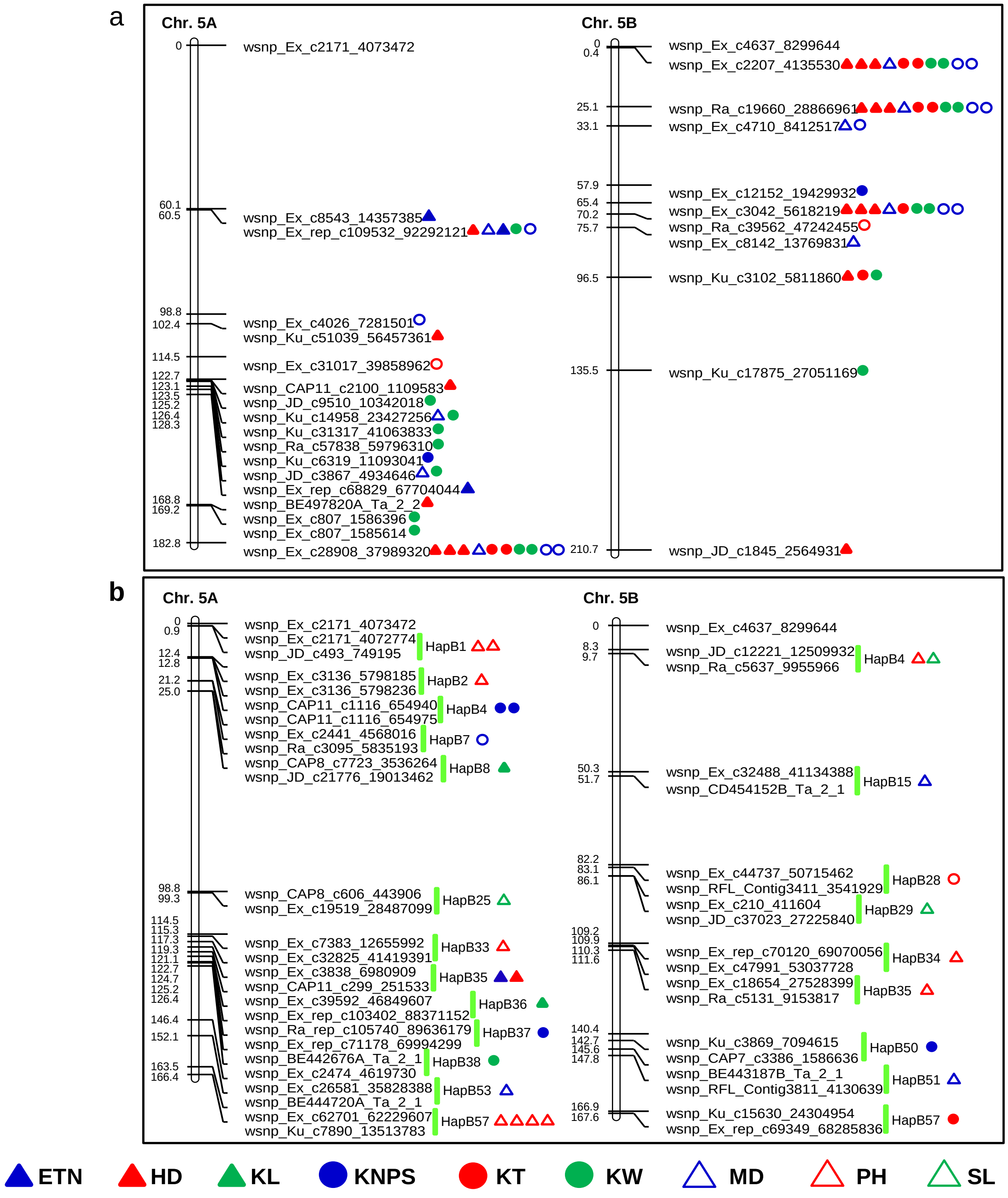

Supplementary Fig. S30_continued Effectiveness comparsion of single SNP-based association (a) with haplotype-based association (b) on chromosomes 2A vs 2B, 3A vs 3B, 4A vs 4B, 5A vs 5B, 6A vs 6B, 7A vs 7B.

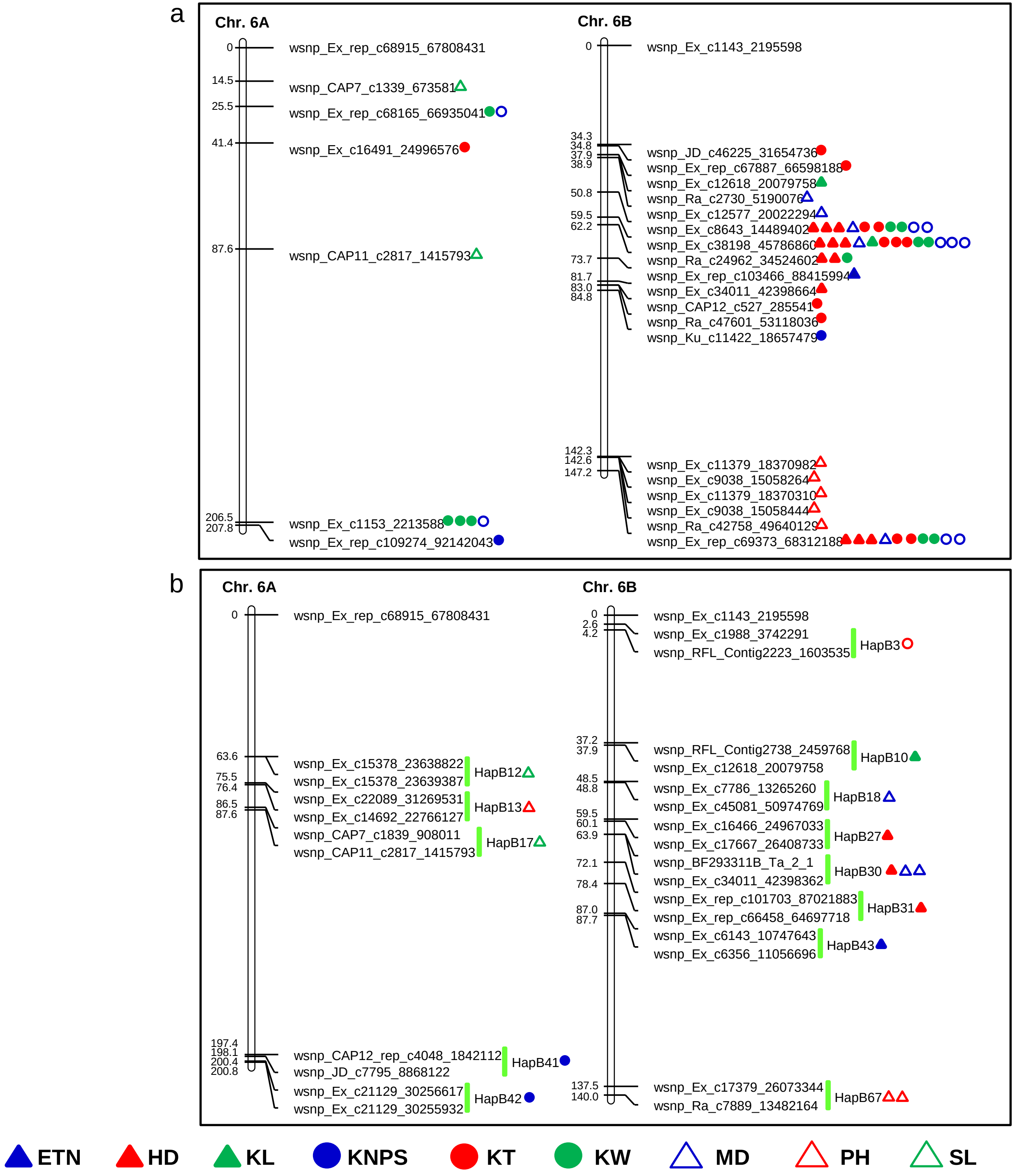

Supplementary Fig. S30_continued Effectiveness comparsion of single SNP-based association (a) with haplotype-based association (b) on chromosomes 2A vs 2B, 3A vs 3B, 4A vs 4B, 5A vs 5B, 6A vs 6B, 7A vs 7B.

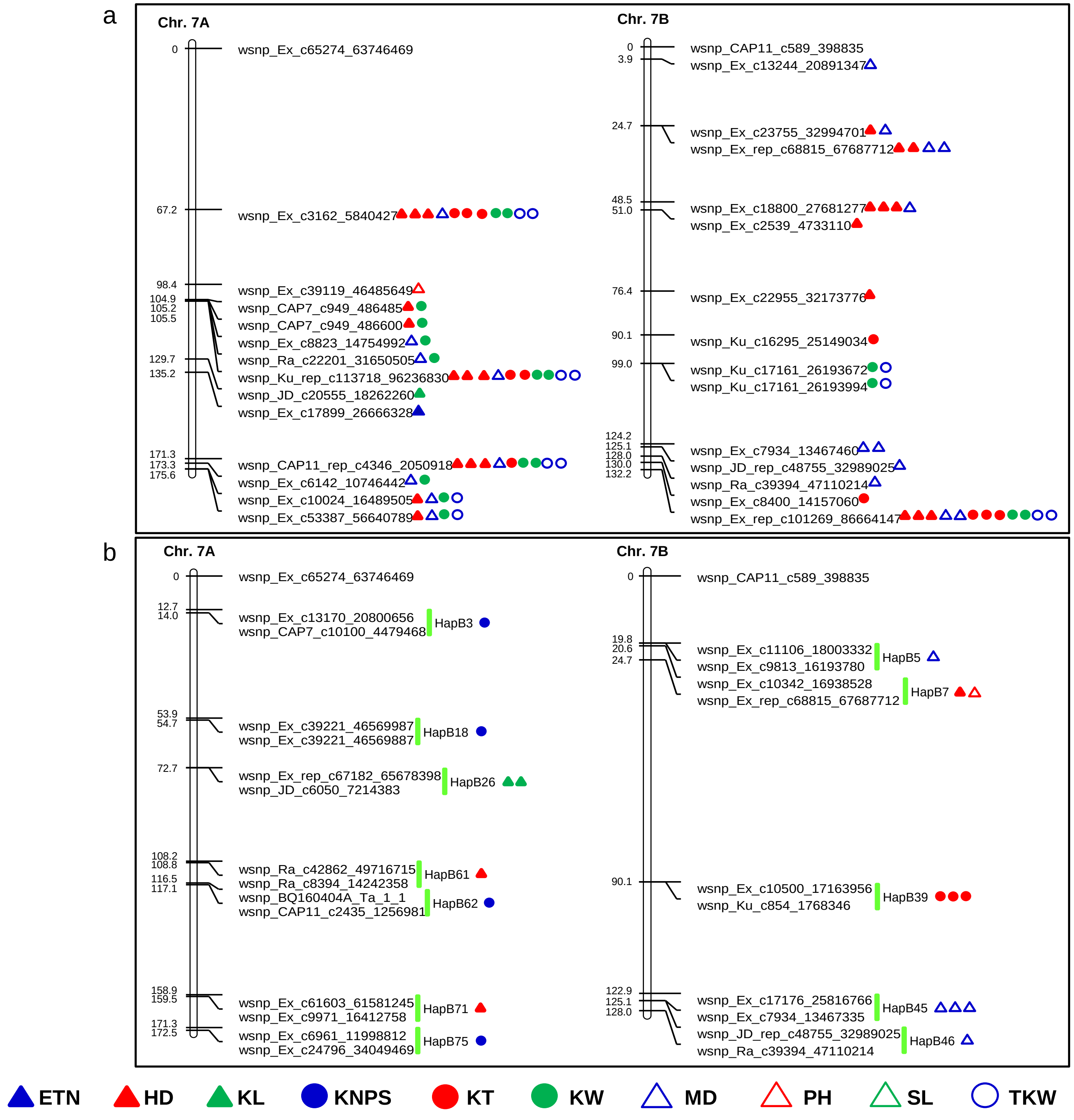

Supplementary Fig. S30_continued Effectiveness comparsion of single SNP-based association (a) with haplotype-based association (b) on chromosomes 2A vs 2B, 3A vs 3B, 4A vs 4B, 5A vs 5B, 6A vs 6B, 7A vs 7B.

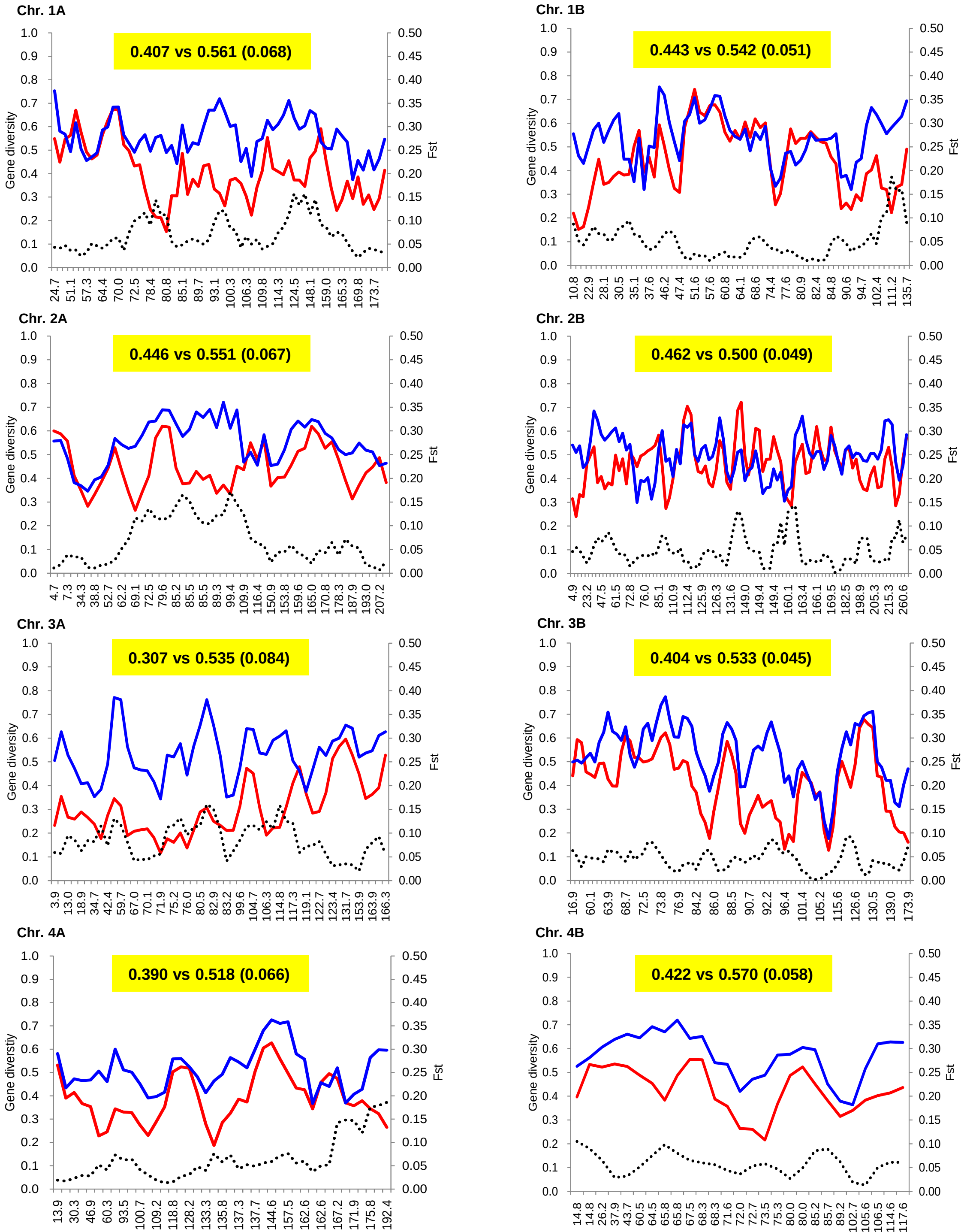

Supplementary Fig. S31 Genetic differentiation (*Fst*) between landraces and modern cultivars and gene diversity along each chromosome in the A and the B genomes revealed by sliding window of three haplotype blocks with step of one block. It indicates that crossing breeding promotes gene exchange and recombination between populations, hence increases gene diversity on most genomic regions. Mean gene diversity in landraces and modern cultivars were given at the top of the each figure that labelled by yellow color, and the *Fst* values between the modern cultivars and the landraces were given in brackets.

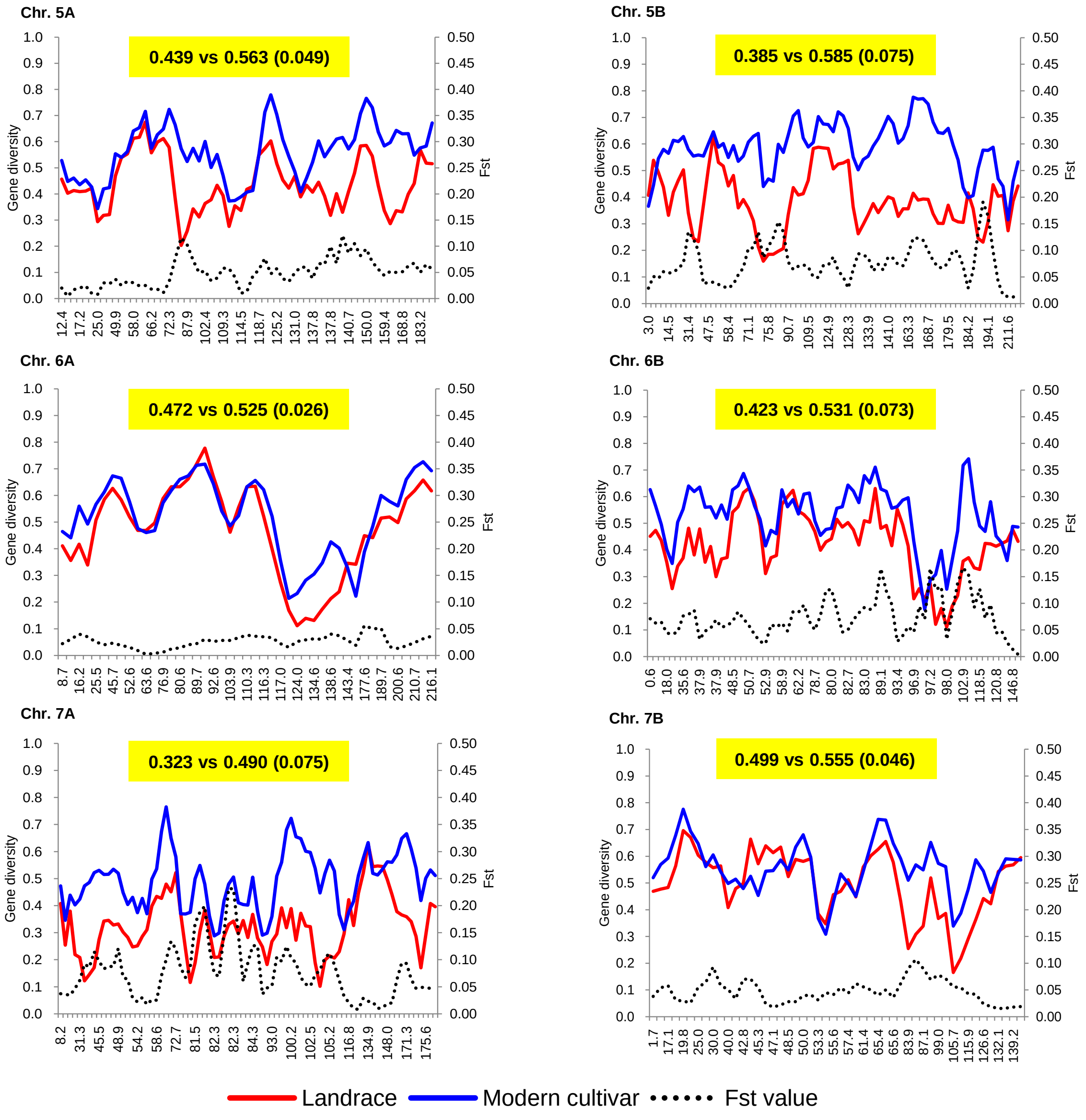

Supplementary Fig. S31_Continued Genetic differentiation (*Fst*) between landraces and modern cultivars and gene diversity along each chromosome in the A and the B genomes revealed by sliding window of three haplotype blocks with step of one block. It indicates that crossing breeding promotes gene exchange and recombination between populations, hence increases gene diversity on most genomic regions. Mean gene diversity in landraces and modern cultivars were given at the top of the each figure that labelled by yellow color, and the *Fst* values between the modern cultivars and the landraces were given in brackets.

Supplementary Table S2. Genome and chromosomes distribution characters of polymorphic SNPs with only one chromosome locus of the 9K CHIP in Chinese wheat mini core collections.

	1	2	3	4	5	6	7	Total
A	419	304	340	315	357	342	343	2420
B	295	529	373	140	472	351	236	2396
D	78	80	31	27	47	43	34	340
Total	792	913	744	482	876	736	613	5156

Supplementary Table S3. Gene diversity comparison among diploid, tetraploid and hexaploid wheats based on A- and B-genome specific SNP markers.

Genome	Homologous group	No.of SNP	**Diploid**		**Tetraploid**		**Landrace**		**Modern cultivar**	
			Mean±S.E	Range	Mean±S.E	Range	Mean±S.E	Range	Mean±S.E	Range
A	1	419	0.067±0.011	0-1	0.322±0.010	0-1	0.186±0.008	0-0.5	0.344±0.007	0-0.5
	2	304	0.073±0.013	0-1	0.357±0.012	0-1	0.279±0.008	0-0.5	0.396±0.007	0.010-0.5
	3	340	0.079±0.012	0-1	0.292±0.012	0-1	0.166±0.007	0-0.5	0.353±0.008	0-0.5
	4	315	0.061±0.013	0-1	0.274±0.013	0-1	0.212±0.008	0-0.5	0.306±0.010	0-0.5
	5	357	0.090±0.013	0-1	0.283±0.013	0-1	0.254±0.008	0-0.5	0.371±0.007	0.019-0.5
	6	342	0.110±0.013	0-1	0.350±0.012	0-1	0.328±0.008	0-0.5	0.352±0.008	0.019-0.5
	7	343	0.069±0.013	0-1	0.350±0.011	0-1	0.187±0.008	0-0.5	0.306±0.009	0-0.5
	**Total**	**2420**	**0.079±0.005**	**0-1**	**0.318±0.004**	**0-1**	**0.229±0.003**	**0-0.5**	**0.347±0.003**	**0-0.5**
B	1	295	0.122±0.017	0-1	0.313±0.013	0-1	0.253±0.010	0-0.5	0.346±0.009	0-0.5
	2	529	0.109±0.012	0-1	0.367±0.009	0-1	0.247±0.007	0-0.5	0.298±0.007	0-0.5
	3	373	0.133±0.016	0-1	0.318±0.011	0-1	0.256±0.008	0-0.5	0.352±0.008	0-0.5
	4	140	0.089±0.021	0-1	0.313±0.018	0-1	0.230±0.013	0-0.5	0.358±0.011	0.038-0.5
	5	472	0.122±0.013	0-1	0.278±0.010	0-1	0.213±0.007	0-0.5	0.381±0.006	0-0.5
	6	351	0.107±0.015	0-1	0.336±0.012	0-1	0.266±0.009	0-0.5	0.370±0.008	0-0.5
	7	236	0.127±0.019	0-1	0.347±0.011	0-1	0.287±0.011	0-0.5	0.363±0.009	0-0.5
	**Total**	**2396**	**0.117±0.006**	**0-1**	**0.326±0.004**	**0-1**	**0.248±0.003**	**0-0.5**	**0.349±0.003**	**0-0.5**

Supplementary Table S5. Comparison of haplotype block number and length between the A and the B genomes.

Genome	No. of HapBs			HapB length		
	Mean±S.E	Range	*P*	Mean±S.E	Range	*P*
A	58.0±5.0	43-81	0.341	1.23±0.10	0-12.1	0.025
B	67.4±8.1	29-96		0.96±0.08	0-13.6	

HapB: haplotype block

Supplementary Table S7. Summary of single SNP markers-based association results for yield-related traits on each chromosome.

Chr.	Associated markers	Associated signals	Associated traits	Traits name	Mean R^2	R^2 range	Favored alleles	% of FA
1A	18	67	6	HD、MD、PH、TKW、KW、KT	5.08	0.03-28.15	44	65.67
1B	10	23	8	HD、MD、PH、KNPS、ETN、TKW、KW、KT	7.91	0.45-20.42	19	82.61
1D	2	2	1	KNPS	9.93	9.69-10.16	2	100.00
2A	19	52	9	HD、MD、PH、KNPS、SL、TKW、KL、KW、KT	4.11	0.002-24.10	31	59.62
2B	22	90	6	HD、MD、PH、TKW、KW、KT	3.90	0.002-18.80	56	62.22
2D	3	14	5	HD、MD、TKW、KW、KT	5.66	0.22-16.82	11	78.57
3A	18	77	5	HD、MD、TKW、KW、KT	6.52	0.002-35.17	55	71.43
3B	22	140	7	HD、MD、PH、TKW、KL、KW、KT	4.76	0.01-22.13	85	60.71
3D1	1	4	4	HD、MD、TKW、KW	3.29	1.08-6.31	3	75.00
4A	22	46	7	HD、MD、PH、KNPS、TKW、KW、KT	7.60	0.06-20.98	33	71.74
4B	12	50	7	HD、MD、PH、ETN、TKW、KL、KW	4.61	0.02-17.72	36	72.00
5A	17	32	8	HD、MD、KNPS、ETN、SN、TKW、KW、KT	4.47	0.0003-19.20	12	37.50
5B	10	39	7	HD、MD、KNPS、SN、TKW、KW、KT	2.01	0.001-14.76	14	35.90
5D3cult	5	22	7	HD、MD、PH、SN、TKW、KW、KT	6.00	0.02-14.16	12	54.55
6A	6	10	5	KNPS、SL、TKW、KW、KT	4.47	0.002-12.79	6	60.00
6B	19	51	9	HD、MD、PH、KNPS、ETN、TKW、KL、KW、KT	4.27	0.0001-15.88	35	68.63
6D2	1	1	1	PH	4.06	4.06	1	100.00
7A	13	51	8	HD、MD、PH、ETN、TKW、KL、KW、KT	5.59	0.02-15.93	42	82.35
7B	14	35	5	HD、MD、TKW、KW、KT	4.86	0.01-14.71	24	68.57
Total/Mean	234	806	11	HD、MD、PH、SN、SL、KNPS、ETN、TKW、KL、KW、KT	4.99	0.0001-35.17	521	64.64

Supplementary Table S8. Summary of single SNP markers-based association results for each yield-related trait.

Chr.	Associated markers	Associated signals	Associated chr.	Chr. name	Mean R^2	R^2 range	Favored alleles	% of FA
ETN	7	8	5	1B、4B、5A、6B、7A	3.67	0.17-9.15	4	50.00
HD	97	212	16	1A、1B、2A、2B、2D、3A、3B、3D1、4A、4B、5A、5B、5D3、6B、7A、7B	2.76	0.001-20.98	102	48.11
KL	11	14	5	2A、3B、4B、6B、7A	4.85	0.002-10.37	8	57.14
KNPS	12	12	8	1B、1D、2A、4A、5A、5B、6A、6B	9.30	5.25-12.79	12	100.00
KT	67	116	15	1A、1B、2A、2B、2D、3A、3B、4A、5A、5B、5D3、6A、6B、7A、7B	3.74	0.0001-15.30	73	62.93
KW	104	159	17	1A、1B、2A、2B、2D、3A、3B、3D1、4A、4B、5A、5B、5D3、6A、6B、7A、7B	6.39	0.0003-21.46	121	76.10
MD	111	120	16	1A、1B、2A、2B、2D、3A、3B、3D1、4A、4B、5A、5B、5D3、6B、7A、7B	4.17	0.003-24.10	74	61.67
PH	18	21	11	1A、1B、2A、2B、3B、4A、4B、5D3、6B、6D2、7A	6.70	0.36-17.71	16	76.19
SL	4	6	2	2A、6A	12.49	3.37-16.92	6	100.00
TKW	81	135	17	1A、1B、2A、2B、2D、3A、3B、3D1、4A、4B、5A、5B、5D3、6A、6B、7A、7B	7.77	0.004-35.17	102	75.56
SN	3	3	3	5A、5B、5D3	5.01	2.03-8.79	3	100.00
Total/Mean	515	806	19	1A、1B、1D、2A、2B、2D、3A、3B、3D1、4A、4B、5A、5B、5D3、6A、6B、6D2、7A、7B	4.99	0.0001-35.17	521	64.64

Supplementary Table S10. Summary of haplotype-based association results of 11 yield-related traits on each chromosome.

Chr.	Associated blocks	Associated signals	Associated traits	Traits name	Mean R^2	R^2 range
1A	8	15	6	MD、PH、KNPS、SN、TKW、KL	20.01	2.93-34.26
1B	19	32	8	HD、MD、PH、KNPS、ETN、SN、SL、TKW	12.79	1.24-23.54
2A	8	15	7	HD、MD、KNPS、ETN、SL、TKW、KL	17.60	6.22-30.48
2B	13	24	6	HD、MD、PH、KNPS、ETN、SL	12.11	4.56-27.30
3A	10	14	5	HD、MD、PH、KNPS、SL	8.33	0.41-19.38
3B	8	11	6	HD、MD、PH、SN、SL、KL	19.71	1.61-37.42
4A	5	9	6	HD、MD、KNPS、KL、KW、KT	13.71	8.66-28.02
4B	5	9	5	HD、MD、PH、KNPS、SL	12.51	5.04-21.14
5A	13	19	8	HD、MD、PH、KNPS、ETN、SL、KL、KW	16.11	0.76-33.70
5B	9	10	6	MD、PH、KNPS、SL、SN、KT	11.40	3.33-22.30
6A	5	5	3	PH、KNPS、SL	5.06	4.01-6.21
6B	8	11	6	HD、MD、PH、ETN、SN、KL	12.89	4.73-27.36
7A	7	8	3	HD、KNPS、KL	15.03	4.01-37.62
7B	5	10	4	HD、MD、PH、KT	13.77	8.82-24.81
Total/Mean	123	192	11	HD、MD、PH、SN、SL、KNPS、ETN、TKW、KL、KW、KT	13.95	0.41-37.62

Supplementary Table S11. Summary of haplotype-based association results for each yield-related trait.

Chr.	Associated blocks	Associated signals	Associated chr.	Chr. name	Mean R^2	R^2 range
ETN	6	6	5	1B、2A、2B、5A、6B	10.42	1.35-18.39
HD	24	31	11	1B、2A、2B、3A、3B、4A、4B、5A、6B、7A、7B	12.38	0.42-27.36
KL	10	13	7	1A、2A、3B、4A、5A、6B、7A	22.32	8.13-37.62
KNPS	20	21	11	1A、1B、2A、2B、3A、4A、4B、5A、5B、6A、7A	11.66	4.01-20.70
KT	3	5	3	4A、5B、7B	11.52	8.82-16.25
KW	3	5	2	4A、5A	12.20	2.67-28.02
MD	31	35	12	1A、1B、2A、2B、3A、3B、4A、4B、5A、5B、6B、7B	12.32	0.41-25.49
PH	26	43	11	1A、1B、2B、3A、3B、4B、5A、5B、6A、6B、7B	16.44	0.76-37.42
SL	13	17	9	1B、2A、2B、3A、3B、4B、5A、5B、6A	9.14	4.01-21.41
TKW	7	11	6	1A、1B、2D、3B、5B、6B	22.73	1.24-34.26
SN	5	5	5	1A、1B、1D、2A、5A	7.09	2.93-9.54
Total/Mean	148	192	14	1A、1B、2A、2B、3A、3B、4A、4B、5A、5B、6A、6B、7A、7B	13.95	0.41-37.62
